# Non‐Canonical Function of DDX21 in Fetal Hematopoiesis Antagonizes KDM5A‐Mediated Repression of Ribosomal and Hematopoietic Genes

**DOI:** 10.1002/advs.78524

**Published:** 2026-11-09

**Authors:** Adrian On‐Wah Leung, Chang Li, Xianyin Huang, Zhuming Fan, Yuning Chen, Jiaxing Liu, Hong Man Lin, Xiaoming Cui, Fujia Zhang, Ruoxi Niu, Kam Tong Leung, Boxian Huang, Hoi‐Hung Cheung

**Affiliations:** ^1^ School of Biomedical Sciences Faculty of Medicine The Chinese University of Hong Kong Hong Kong SAR China; ^2^ Centre For Regenerative Medicine and Health Hong Kong Institute of Science and Innovation Chinese Academy of Sciences Beijing China; ^3^ Institute of High Energy Physics Chinese Academy of Sciences Beijing China; ^4^ Department of Paediatrics Faculty of Medicine The Chinese University of Hong Kong Hong Kong SAR China; ^5^ State Key Laboratory of Reproductive Medicine Nanjing Medical University Nanjing China; ^6^ Key Laboratory for Regenerative Medicine School of Biomedical Sciences Faculty of Medicine Ministry of Education The Chinese University of Hong Kong Hong Kong SAR China

**Keywords:** anemia, DDX21, hematopoiesis, HSPC, KDM5A, ribosomopathy

## Abstract

Ribosome biogenesis is crucial for meeting the elevated biosynthetic demands of rapidly expanding fetal hematopoietic stem and progenitor cells (HSPCs) during fetal development. Ddx21 is a DEAD‐box RNA helicase required for ribosome biogenesis, but its role in fetal hematopoiesis remains undefined. Here, we show that hematopoietic‐specific deletion of *Ddx21* in mice results in severe fetal anemia, HSPC depletion, and loss of reconstitution capacity. Mechanistically, Ddx21 directly binds the histone demethylase Kdm5a via its helicase D2 domain in an RNA‐dependent manner. Ddx21 and Kdm5a co‐occupy active promoters of hematopoietic genes (*Gata1*, *cKit*) and rDNA. Unexpectedly, Ddx21 loss does not displace Kdm5a from chromatin but reduces H3K4me3 at these loci, repressing both hematopoietic and ribosomal gene transcription. Although p53 activation drives apoptosis, p53 inhibition only partially rescues hematopoietic failure. Notably, combined pharmacological inhibition of Kdm5a and p53 synergistically restores H3K4me3, protein synthesis, and HSPC colony formation. Our findings reveal a non‐canonical function of Ddx21 in epigenetic regulation by counteracting Kdm5a‐mediated repression to sustain active transcription. The DDX21‐KDM5A complex represents a previously unrecognized chromatin modulator balancing ribosome biogenesis and epigenetic repression, with implications for understanding the pathogenesis of ribosomopathies and developing therapeutic strategies targeting the epigenome‐ribosome interface.

## Graphical abstract

This study demonstrates a mechanism by which RNA helicase DDX21 coordinates the transcription of hematopoietic and ribosomal genes by interacting with the H3K4me3 demethylase KDM5A. The DDX21‐KDM5A complex co‐occupies active genes in hematopoietic progenitors, thereby ensuring their rapid proliferation during fetal hematopoiesis. Loss of DDX21 causes hematopoietic stem cell depletion and consequently hematopoietic failure, resembling the pathogenesis of ribosomopathies.

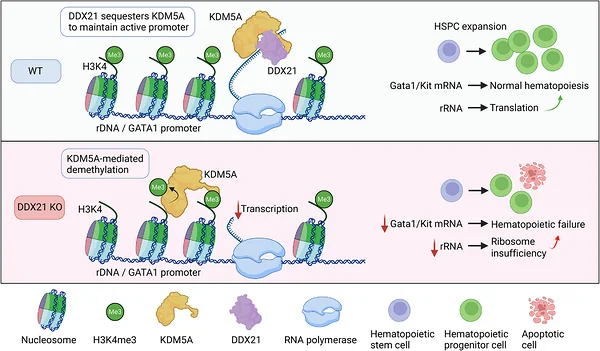

## 1 Introduction

Hematopoiesis is a dynamic process essential for lifelong production of different blood cells. During fetal development, hematopoietic stem and progenitor cells (HSPCs) undergo rapid expansion to meet the escalating demand for fetal blood [1, 2]. This phase relies heavily on robust protein synthesis driven by ribosome biogenesis, a tightly coordinated process involving rDNA transcription, ribosomal protein (RP) synthesis, and subsequent ribosome assembly [3]. Perturbations to ribosome biogenesis disrupt cellular homeostasis, leading to ribosomopathies such as Diamond‐Blackfan anemia (DBA) characterized by hematopoietic failure [4, 5, 6]. While mutations in structural RP genes (e.g., *RPS19* and *RPL5*) are well documented for causing ribosomopathies, the roles of a wide range of non‐structural ribosome biogenesis factors [7] in regulating hematopoiesis have not been fully defined. As ribosome biogenesis is proposed to influence stem cell fate decisions and balance self‐renewal and differentiation [8, 9, 10, 11], the loss of ribosome biogenesis factors is thought to disturb the hematopoietic system. For instance, germline mutations of *DDX41*, a ribosome assembly factor for processing snoRNA, cause ineffective hematopoiesis and myelodysplasia [12, 13]. However, some ribosome biogenesis factors (e.g., *HectD1*) are more tolerated when deleted at the fetal stage but present abnormalities in the adult HSPCs [10]. These studies suggest a diverse, context‐specific role for ribosome biogenesis factors in regulating hematopoiesis.

DDX21, a DEAD‐box RNA helicase, is a key ribosome biogenesis factor implicated in rDNA transcription, rRNA processing, and ribosomal subunit maturation [14, 15, 16]. Depletion of Ddx21 in preimplantation mouse embryos results in developmental aplasia [17]. Moreover, DDX21 mislocalization and impaired ribosome biogenesis are observed in neural crest cells in disease models of ribosomopathies, such as Treacher Collins syndrome and DBA [18]. In zebrafish, ddx21 is required for Vegfc‐driven developmental lymphangiogenesis by balancing endothelial cell ribosome biogenesis and p53 function [19]. In epidermal progenitors, DDX21 loss causes splicing alterations but does not enhance apoptosis [20]. These studies highlight the tissue‐specific, non‐redundant requirement of DDX21 for the proliferation and differentiation of progenitors during development. Recent work has shown that Ddx21 is required for adult HSC homeostasis by preventing nucleolar stress and p53 activation [21]. However, its function during the rapid expansion of fetal HSPCs, which have especially high biosynthetic demands, remains undefined.

Here, we investigate the loss‐of‐function of *Ddx21* during fetal hematopoiesis. Using conditional knockout (cKO) mice, we demonstrate that deletion of *Ddx21* in hematopoietic cells results in severe anemia, liver hypoplasia, and HSPC depletion in the fetus. Proteomic and genomic analyses reveal that Ddx21 interacts with the histone demethylase Kdm5a to preserve an active chromatin state at not only ribosomal but also hematopoietic genes, thus maintaining an active transcriptional output. Loss of Ddx21 downregulates H3K4me3, a histone substrate of Kdm5a, leading to epigenetic repression of *Gata1*, *cKit*, and rDNA, and consequently hematopoietic defects. Our findings suggest a non‐canonical function of the RNA helicase in the epigenetic control of hematopoietic genes and rDNA transcription via the Ddx21‐Kdm5a complex, offering insights into the pathogenesis of ribosomopathies and highlighting therapeutic potential by targeting the epigenome‐ribosome interface.

## 2 Results

### 2.1 Knockout of Ddx21 in Hematopoietic Cells Results in Defective Hematopoiesis and Fetal Anemia

As germline deletion of *Ddx21* in mice is embryonically lethal, we generated floxed *Ddx21* mice (*Ddx21^f/f^*) for tissue‐specific cKO (Figure S1A). By crossing with *Vav1‐Cre* mice, *Vav1‐Cre;Ddx21^f/f^* (*Ddx21^cKO^*) showed complete deletion of *Ddx21* in fetal hematopoietic cells (Figure S1B–E). Notably, viable *Ddx21^cKO^* embryos declined dramatically after E16.5 (Figure 1A). *Ddx21^cKO^* embryos were pallid, with significantly smaller fetal liver (FL) compared to stage‐matched wild‐type (WT) littermates (Figure 1B and Figure S1F). Increased cell death was evident in the FL starting from E12.5, accompanied by reduced cellularity (Figure 1C,D). Hemoglobin content per FL was also markedly decreased, indicating severe fetal anemia (Figure 1E). To investigate whether hematopoiesis was disrupted, we used flow cytometry (gating strategy shown in Figure S2) to analyze lineage‐depleted (Lin^–^) cells that contain Lin^–^Sca1^+^cKit^+^ (LSK) HSPCs and the committed Lin^–^Sca1^–^cKit^+^ (LS^–^K) progenitors. In *Ddx21^cKO^*, both LSK and LS^–^K cells, represented as a percentage of total live FL cells, were found to be dramatically decreased at E13.5 and were nearly undetectable by E18.5, a stark contrast to the robust expansion in *Ddx21^WT^* (Figure 1F). Further analysis of the LSK subpopulations, including hematopoietic stem cells (HSC), multipotent progenitors MPP1 (balanced), MPP2 (megakaryocyte/erythrocyte‐biased), and MPP3&4 (myelocyte/lymphocyte‐biased), defined by SLAM antigens CD150/CD48 [22], revealed significant depletion in both E13.5 and E16.5 FLs (Figure 1G). Likewise, LS^–^K progenitors containing committed CMP (common myeloid progenitor), GMP (granulocyte‐monocyte progenitor), and MEP (megakaryocyte‐erythroid progenitor) were also reduced at E16.5 (Figure 1G). Absolute cell counts confirmed these reductions in *Ddx21^cKO^* FL (Figure S3A and Table S1). The profound loss of HSPCs, especially erythroid‐biased MPP2 and MEP, likely underlies the anemia phenotype and embryonic lethality.

**FIGURE 1:**
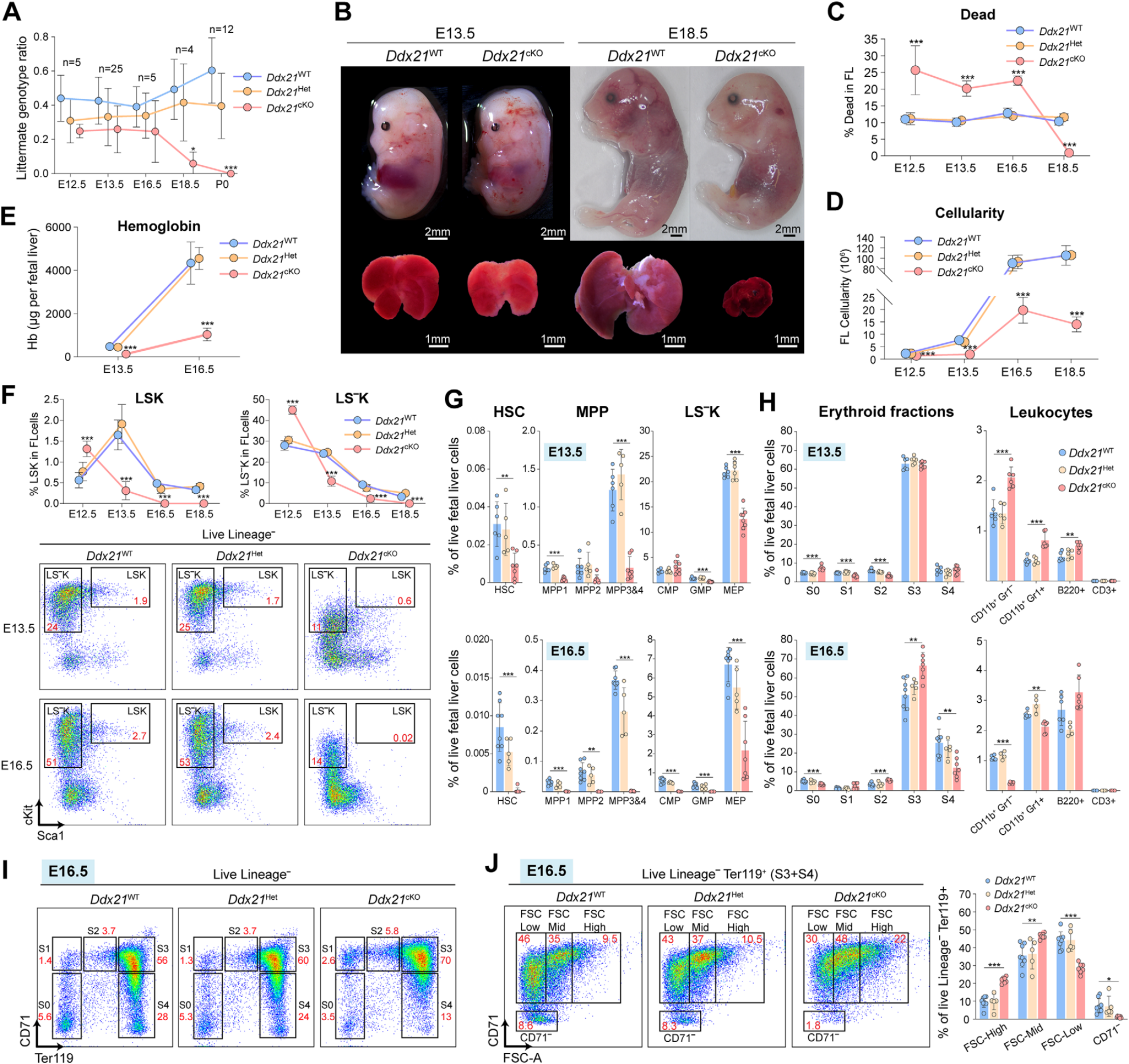
*Ddx21* knockout in fetal hematopoietic cells results in severe hematopoietic defect and embryonic lethality. (A) Littermate genotype ratio of *Ddx21^f/f^* or *Ddx21^f/+^* (*Ddx21^WT^*), *Vav1‐Cre; Ddx21^f/+^* (*Ddx21^Het^*) and *Vav1‐Cre; Ddx21^f/f^* (*Ddx21^cKO^*) embryos generated from mating pair: *Vav1‐Cre; Ddx21^f/+^* x *Ddx21^f/f^*. n represents the number of litters analyzed. Longitudinal differences for each genotype were compared; p‐values relative to E12.5 baseline are plotted. (B) Representative gross morphology of whole embryo and fetal liver (FL) of *Ddx21^WT^* and *Ddx21^cKO^* embryos at E13.5 (n = 8) and E18.5 (n = 3). (C‐J) Physical properties and population percentages of *Ddx21^WT^*, *Ddx21^Het^* and *Ddx21^cKO^* fetal livers from E13.5 to E18.5. p‐value compared against *Ddx21^WT^*. Number in flow gates represents average percentage of parent gate. (C) Percentage of dead cells (n = 3‐14). (D) Cellularity (n = 3‐14). (E) Hemoglobin content of whole fetal liver (n = 5‐8). (F) Lineage^–^Sca1^+^cKit^+^ (LSK) and Lineage^–^Sca1^–^cKit^+^ (LS^–^K) percentages over live FL cells. Representative cKit/Sca1 flow plots of LSK and LS^–^K populations in live Lineage^–^ cells (n = 3‐8). (G) LSK and LS^–^K populations in total live FL cells (n = 5‐8). (H) Erythroid and leukocyte populations in total live FL cells (n = 5‐8). (I) Representative CD71/Ter119 flow plots of E16.5 erythroid fractions in live Lineage^–^ cells (n = 5‐8). (J) Representative CD71/FSC‐A flow plots and quantification of E16.5 live Lineage^–^ Ter119^+^ erythroid cells. ^*^ *p* < 0.05, ^**^ *p* < 0.01, ^***^ *p* < 0.001.

### 2.2 Effect of Ddx21 Knockout on Erythroid Maturation

To further investigate the impact of Ddx21 loss on erythropoiesis, the predominant hematopoietic event in the fetus, we analyzed the maturation of FL erythroblasts by gating Lin^–^ cells and subdivided the erythroid fractions (S0‐S4) based on CD71/Ter119 expression [23] (Figure S2). At E16.5, when fetal erythrocytes start to mature, the absolute and relative numbers of early committed erythroblasts (S1, Ter119^–^CD71^high^) and late immature erythroblasts (S2 & S3, Ter119^+^CD71^high^) were increased in *Ddx21* ^*cKO*^, at the expense of the number of mature erythrocytes (S4, Ter119^+^CD71^low^) (Figure 1H,I and Figure S3B). Among the late erythroid populations (S3+S4), forward scatter (FSC)‐based cell size gating revealed a shift toward the less mature (larger size, FSC‐^high^) erythroblasts in *Ddx21^cKO^* (22% in *Ddx21^cKO^* vs 9.5% in *Ddx21^WT^*). As a result, terminally differentiated reticulocytes/erythrocytes (Ter119^+^CD71^–^FSC^low^) were decreased (Figure 1J and Figure S3C). These observations indicate a maturation delay or differentiation blockade during terminal erythropoiesis.

### 2.3 Knockout of Ddx21 Abolishes Hematopoietic Reconstitution

The FL contains definitive HSPCs that can reconstitute the bone marrow (BM) and differentiate into multiple lineages upon transplantation into myeloablated recipient mice, a gold standard for assessing the self‐renewal and hematopoietic reconstitution capabilities of HSPCs [24]. We performed competitive bone marrow transplantation (BMT) by injecting 1.5 × 10^6^ live FL cells from *Ddx21* ^*WT*^ or *Ddx21^cKO^* E13.5 embryos (CD45.2^+^) with 3 × 10^5^ competitive WT BM cells (CD45.1^+^) into lethally irradiated adult recipients (CD45.1^+^;CD45.2^+^) (Figure 2A and Figure S4A). As anticipated, mice injected with *Ddx21^WT^* FL showed high peripheral blood (PB) chimerism (47% CD45.2^+^) at 1 month post‐transplantation, whereas *Ddx21* ^*cKO*^ was less than 1% (Figure 2B). Similarly, chimerism in BM was significantly lower for *Ddx21^cKO^*, and the HSPC fractions (LSK and LS^–^K) were virtually undetectable, indicating a failure to engraft and reconstitute the BM (Figure 2C).

**FIGURE 2:**
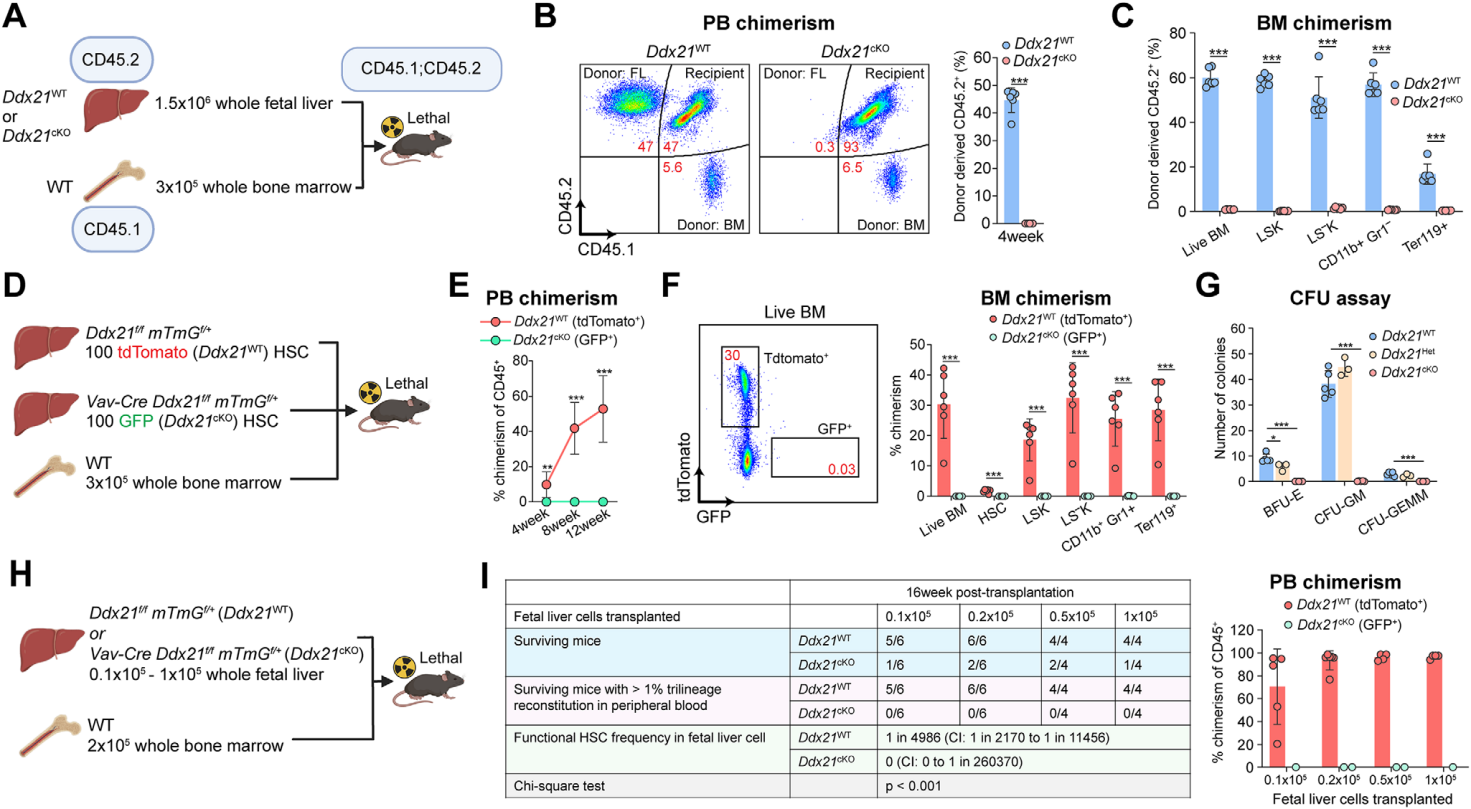
Ddx21‐deficient HSPCs fail to reconstitute hematopoiesis. (A‐C) Transplantation of *Ddx21* ^*WT*^ and *Ddx21^cKO^* fetal liver cells from E13.5 littermate embryos (n = 6). (A) Schematic diagram of fetal liver cell transplantation. (B) Peripheral blood (PB) CD45.2^+^ chimerism among CD45^+^ cells at 4 weeks post‐transplantation. Representative flow plots of live CD45^+^ cells were shown. Number in flow gates represents average percentage of parent gate. (C) Bone marrow (BM) CD45.2^+^ chimerism at 4 weeks post‐transplantation. (D‐F) Competitive transplantation of *Ddx21^WT^* and *Ddx21^cKO^* fetal liver HSC (live, LSK, CD48^–^, CD150^+^) from E13.5 littermate embryos carrying *mTmG^f/+^* allele (n = 6). (D) Schematic diagram of competitive HSC transplantation. (E) PB chimerism among CD45^+^ cells was monitored over 12 weeks post‐transplantation. (F) BM chimerism at 12 weeks post‐transplantation. Representative flow plot of live BM cells was shown. Number in flow gates represents average percentage of parent gate. (G) CFU assay of E13.5 FACS‐sorted fetal liver Lineage^–^cKit^+^ (LK) cells of *Ddx21* ^*WT*^, *Ddx21^Het^* and *Ddx21^cKO^* embryos using MethoCult^TM^ M3434 (n = 3‐5). (H‐I) Limiting transplantation of *Ddx21^WT^* and *Ddx21^cKO^* fetal liver cells from E13.5 littermate embryos for determining HSC frequency (n = 4‐6). (H) Schematic diagram of limiting fetal liver cell transplantation. (I) Surviving mice and PB CD45 chimerism at 16 weeks post‐transplantation. HSC frequency calculated with Extreme Limiting Dilution Analysis (ELDA). Number in flow gates represents average percentage in parent gate. ^*^ *p* < 0.05, ^**^ *p* < 0.01, ^***^ *p* < 0.001.

To account for the difference in HSPC numbers between *Ddx21* ^*WT*^ and *Ddx21^cKO^* FL that could confound the transplantation results, a competitive co‐transplantation experiment was performed using equal numbers of HSCs. We transplanted 100 tdTomato^+^ live HSCs (LSK CD150^+^ CD48^–^) of *Ddx21* ^*WT*^ (*Ddx21^f/f^;mTmG^f/+^*) and 100 GFP^+^ HSCs of *Ddx21^cKO^* (*Vav1‐Cre;Ddx21^f/f^;mTmG^f/+^*) with 3 × 10^5^ WT BM cells into myeloablated adult mice (Figure 2D and Figure S4B). PB chimerism was monitored every 4 weeks for 12 weeks. Similar to FL transplantation, *Ddx21^cKO^* HSCs contributed to less than 0.1% chimerism in both PB and BM at 12 weeks post‐transplantation, highlighting an essential role for Ddx21 in HSC reconstitution (Figure 2E,F). The loss of hematopoietic function was further supported by a hematopoietic colony‐forming unit (CFU) assay using E13.5 FL cells, which demonstrated a complete failure to generate BFU‐E (burst‐forming unit‐erythroid), CFU‐GM (colony‐forming unit‐granulocyte, macrophage), and CFU‐GEMM (CFU‐granulocyte, erythrocyte, macrophage, and megakaryocyte) (Figure 2G and Figure S4C). Additionally, we performed a limiting dilution transplantation assay to quantify the absolute number of functional HSCs in *Ddx21^WT^* and *Ddx21^cKO^* FL (Figure 2H). While *Ddx21^WT^* FL cells yielded a functional HSC frequency of 1 in 4986 cells (95% confidence interval [CI]: 1 in 11456 to 1 in 2170 cells), *Ddx21^cKO^* FL cells showed a profound functional collapse with no positive reconstitution at any cell doses tested (Figure 2I). These results collectively demonstrate a cell‐autonomous function of Ddx21 in safeguarding HPSCs, potentially through regulating HSPC expansion during fetal hematopoiesis.

### 2.4 Activation of p53 and Apoptosis in Ddx21‐Defecient HSPCs

DDX21 loss is known to induce p53‐dependent cell cycle arrest in mutant zebrafish and cultured cancer cells [19, 25], providing a rationale accounting for our observed hematopoietic failure. However, as these molecular events were reported to be cell type‐ and species‐specific [18], the regulatory axis in HSPCs remained elusive. We thus performed RNA‐seq on Lin^–^cKit^+^ (LK) HSPCs and observed a pronounced upregulation of p53 target genes, including *Cdkn1a*, *Fas*, and *Mdm2*, in *Ddx21^cKO^* cells (Figure 3A). Both Kyoto Encyclopedia of Genes and Genomes (KEGG) pathway enrichment and Gene Set Enrichment Analysis (GSEA) revealed significant enrichment of the p53 signaling pathway upon *Ddx21* deletion (Figure 3B,C). Further analysis of p53 downstream targets revealed a concerted upregulation of apoptotic signaling genes and inhibition of cell cycle progression genes in *Ddx21* ^*cKO*^ HSPCs (Figure 3D).

**FIGURE 3:**
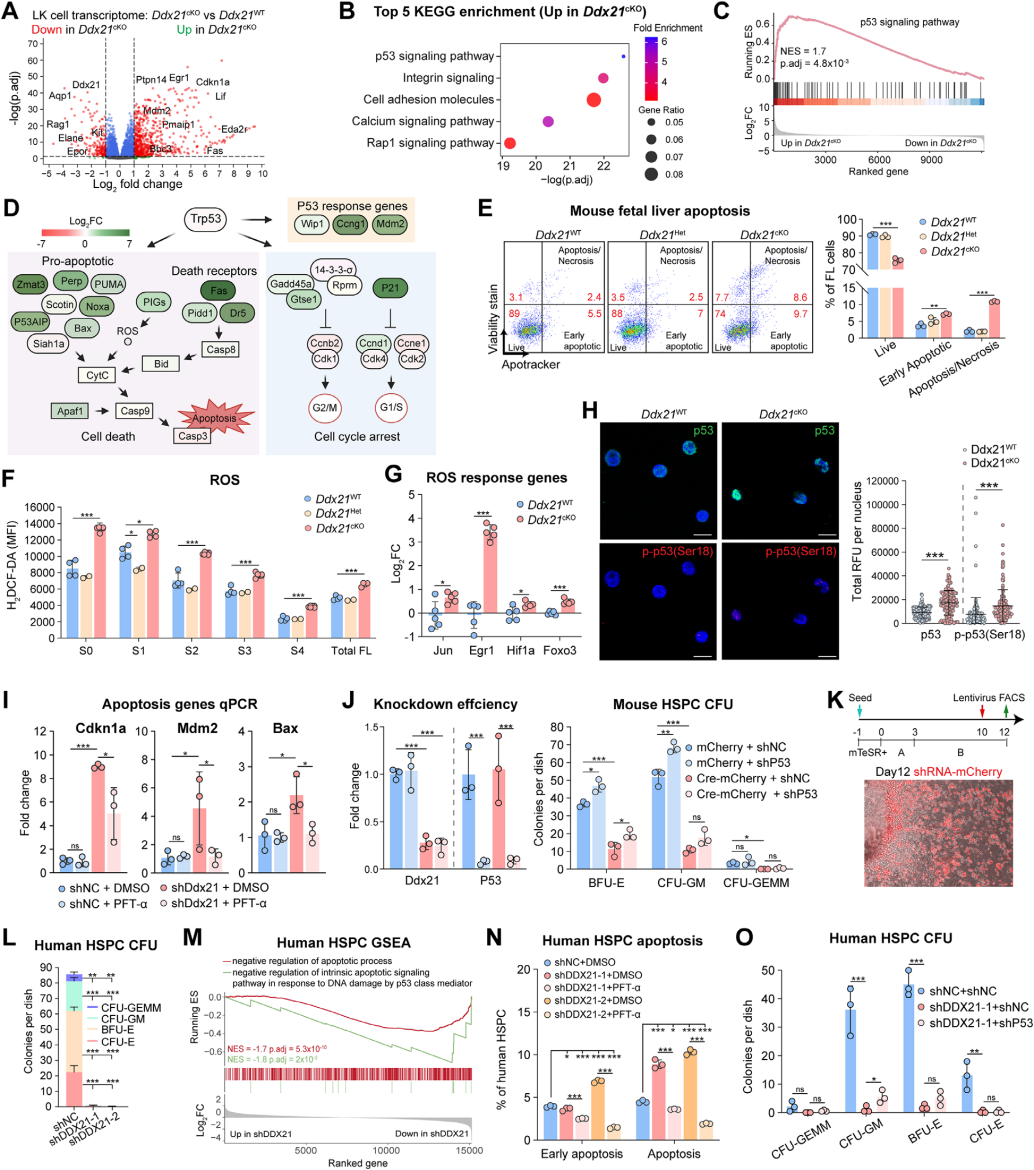
p53 inhibition ameliorates p53‐driven apoptosis but only slightly enhances BFU‐E formation. (A‐D) Transcriptomic analysis between *Ddx21* ^*WT*^ and *Ddx21^cKO^* E13.5 fetal liver Lineage^–^cKit^+^ (LK) cells (n = 5). (A) Volcano plot. (B) Top 5 KEGG enrichment in differential expressed genes (DEG) (Fold change >2, *p.adj* < 0.05) up‐regulated in *Ddx21^cKO^*. (C) KEGG “p53 signaling pathway” GSEA analysis. (D) Expression changes of KEGG “p53 signaling pathway” genes. (E) Flow cytometry apoptosis analysis of E13.5 fetal liver cells. Number in flow gates represents average percentage of total cells (n = 3). (F) Flow cytometry analysis of ROS by 2’,7’‐dichlorodihydrofluorescein diacetate (H_2_DCF‐DA) staining of E16.5 fetal liver erythroid fractions. Geometric mean fluorescence index was reported (n = 3‐4). (G) ROS response gene expression changes from the E13.5 LK cell transcriptome (n = 5). (H) Immunofluorescence staining of p53 and p‐p53(Ser18) in E13.5 live LK cells (n = 126‐152). (I) RT‐qPCR analysis of apoptotic genes in cultured E13.5 LK cells after 3 days of shRNA knockdown and 15 µM Pifithrin‐α (PFT‐α) incubation (n = 3). (J) Knockdown efficiency and CFU assay (MethoCult M3434) of cultured E13.5 *Ddx21^f/f^* fetal liver LK cells after 3 days of lentivral transduction (n = 3). (K‐O) Analysis of human HSPCs (live CD34^+^ CD45^+^ CD43^+^) differentiated from human embryonic stem cells (hESCs) with 2 days of shRNA lentiviral transduction and PFT‐α/DMSO incubation. (K) Schematic diagram of the differentiation. Representative day12 brightfield image overlayed with shRNA‐mCherry fluorscence was shown. (L) CFU assay using MethoCult SF H4636 after shDDX21 knockdown (n = 3). (M) GSEA analysis on transcriptome comparing between shNC and shDDX21 human HSPC (n = 3). (N) Flow cytometry apoptosis analysis using Apotracker and fixable viability stain (n = 3). (O) CFU assay using MethoCult SF H4636 after shDDX21 and shP53 knockdown (n = 3). ^*^ *p* < 0.05, ^**^ *p* < 0.01, ^***^ *p* < 0.001.

To examine the apoptotic event, E13.5 FL cells were analyzed by flow cytometry using Apotracker (an Annexin‐V equivalent dye) and dead cell probe FVS (fixable viability stain). In line with the transcriptome, a significant increase in early apoptosis (Apotracker^+^ FVS^–^) and late apoptosis/necrosis (Apotracker^+^ FVS^+^) was observed (Figure 3E). Studies have demonstrated that high levels of reactive oxygen species (ROS) trigger p53 phosphorylation at Ser18, a molecular marker indicating p53 activation [26]. We measured ROS levels in sorted live cells from E16.5 *Ddx21* ^*cKO*^ FL and found elevated ROS, especially in the erythroid progenitors (Figure 3F). Consistently, ROS response genes such as *Jun*, *Egr1*, *Hif1a*, and *Foxo3* were upregulated (Figure 3G). Additionally, immunofluorescence staining revealed elevated nuclear p53 and phospho‐p53 (Ser18) signals in *Ddx21^cKO^* LK cells, confirming p53 activation (Figure 3H).

### 2.5 Inhibition of p53 Alleviates Cell Death but Not Hematopoietic Function

To determine whether p53 mediates the hematopoietic defects caused by Ddx21 loss, we inhibited p53 with Pifithrin‐α (PFT‐α) and successfully suppressed p53 target genes such as *Cdkn1a*/p21, *Mdm2*, and *Bax* in Ddx21 knockdown HSPCs (Figure 3I). Next, we tested whether p53 inhibition in HSPCs lacking Ddx21 (mediated by p53‐shRNA and Cre‐mCherry lentiviruses) was sufficient to rescue the hematopoietic defect. Despite a high efficiency of p53 knockdown in LK cells, the CFU assay showed only partial rescue of BFU‐E colonies (Figure 3J).

To recapitulate the findings in human hematopoietic cells, we differentiated human HSPCs (CD45^+^CD34^+^CD43^+^) from pluripotent stem cells and silenced DDX21 by two independent shRNAs (shDDX21‐1 and shDDX21‐2) (Figure 3K and Figure S5A,B). Consistent with the mouse *Ddx21* ^*cKO*^ phenotype, DDX21 knockdown in human HSPCs abolished hematopoietic CFU colony formation (Figure 3L). GSEA of DDX21 knockdown versus non‐targeting control (shNC) transcriptomes also highlighted the activation of apoptosis (Figure 3M). In line with mouse p53 inhibition, PFT‐α suppressed apoptosis in DDX21‐silenced human HSPCs (Figure 3N). Similarly, p53 knockdown could only partially restore hematopoietic colony‐forming capabilities in DDX21‐deficient human HSPCs (Figure 3O). Together, these results indicate that p53‐dependent apoptosis is only a partial cause of hematopoietic failure in DDX21‐deficient cells.

### 2.6 Ddx21 Loss Downregulates Ribosome Biogenesis, Protein Translation, and Cell Cycle Arrest in Hematopoietic Progenitors

DDX21 has been implicated in the transcription of rDNA and RP genes [14, 27]. To examine the impact of Ddx21 loss on ribosome biosynthesis, GSEA analysis of the mouse HSPC transcriptome revealed enrichment of the “ribosome biogenesis” gene ontology (GO) term (Figure 4A). The significantly differentially expressed genes (DEGs) (*p.adj* < 0.05) in the “ribosome biogenesis” GO term list were mostly downregulated in *Ddx21* ^*cKO*^ (Figure 4B). We also analyzed the same gene set for human HSPCs, and revealed 43 overlapping DEGs between the two species (Figure 4C and Figure S5C). As the GO term “structural constituent of ribosome” was also enriched by GSEA (Figure 4A), we analyzed the genes encoding RP and rRNA. In *Ddx21* ^*cKO*^ HSPCs, approximately 17% of all expressed RP genes were downregulated (Figure 4D). Consistently, we observed decreased rRNA levels for pre‐rRNA and 18S (Figure 4E). However, the expression of rRNA could not be restored by blocking p53 with PFT‐α (Figure 4F and Figure S5D). The overall downregulation of RP genes and rRNA in *Ddx21^cKO^* hematopoietic progenitors suggests a potential change in the protein translation capacity. By the O‐propargyl‐puromycin (OP‐Puro) incorporation assay, we found a significant reduction in OP‐Puro incorporation in the S0 & S1 erythroid progenitors (Figure 4G). Reduction in protein synthesis was also demonstrated by OP‐Puro incorporation assay in DDX21 knockdown human HSPCs (Figure S5E) and polysome profiling in K562 cells (Figure S6A).

**FIGURE 4:**
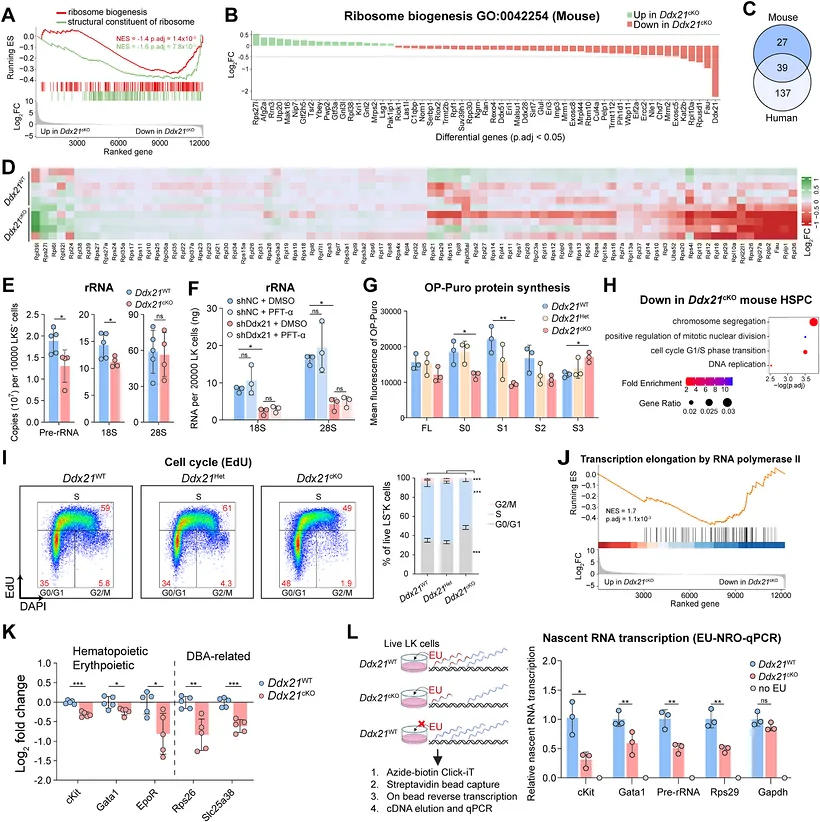
Ddx21 loss inhibits protein synthesis and RNA transcription in hematopoietic progenitors. Mouse HSPC transcriptome analysis comparing between *Ddx21* ^*WT*^ and *Ddx21^cKO^* E13.5 fetal liver LK cells. Human HSPC transcriptome analysis compares shNC and shDDX21 HSPCs. (A) GSEA analysis of ribosome‐related GO terms of mouse transcriptome. (B) Expression changes of differential genes (*p.adj* < 0.05) of mouse transcriptome present in “ribosome biogenesis” GO term. (C) Venn diagram of mouse HSPC and human HSPC differential genes in “ribosome biogenesis” GO term. (D) Heatmap of all expressed ribosomal protein genes in mouse HSPC transcriptome. (E) Absolute quantification of rRNA level in E13.5 LK cells by RT‐qPCR (n = 6). (F) Tapestation quantification of rRNA level of cultured E13.5 LK cells after 3 days of knockdown and 15 µM pifithrin‐α (PFT‐α) incubation (n = 3). (G) O‐propargyl‐puromycin (OP‐Puro) nascent protein synthesis assay of E13.5 fetal liver erythroid fractions after 30 min of 10 µM OP‐Puro incorporation (n = 3). (H) GO enrichment of cell cycle related terms in mouse HSPC transcriptome. (I) EdU/DAPI cell cycle analysis of E13.5 fetal liver live LS^–^K cells 0.5hr after EdU intraperitoneal injection to pregnant mouse. Numbers in red represent average percentage of parent gate (n = 3‐4). (J) GSEA analysis of “transcription elongation by RNA polymerase II” in mouse HSPC transcriptome. (K) Log_2_ fold change (FC) of erythropoietic genes and Diamond‐Blackfan anemia (DBA) genes in mouse HPSC transcriptome. (L) Nascent nuclear run‐on RNA transcription assay by RT‐qPCR on 5‐ethynyluridine (EU) incorporated RNA. E13.5 fetal liver LK cells were cultured in 10 µM EU for 1hr. Nascent RNA was normalized with total RNA (Gapdh RNA before Streptavidin bead capture) to calculate relative nascent RNA transcription (n = 3). ^*^ *p* < 0.05, ^**^ *p* < 0.01, ^***^ *p* < 0.001.

In addition to apoptosis, the lack of hematopoietic expansion in *Ddx21* ^*cKO*^ progenitors may be partly attributed to cell cycle arrest, which is reported to be induced by ribosomal stress [28]. Transcriptome analysis revealed significant enrichment of “chromosome segregation” and “mitotic cell cycle phase transition” GO terms in both human and murine HSPCs (Figure 4H and Figure S5F). To examine the changes in cell cycle and DNA replication in vivo, we injected EdU into pregnant mice at E13.5 and harvested FL cells for cell cycle analysis. Both LS^–^K progenitors and erythroid progenitors (S0‐S3) showed an increase in the G0/G1 phase and concomitant decreases in the S and G2/M phases, indicating a blockade at the G1‐S transition (Figure 4I and Figure S6B).

### 2.7 Transcriptional Suppression in Ddx21 Knockout HSPC

Apart from ribosome biogenesis and cell cycle control, we also observed negative enrichment of the GO term “transcription elongation by RNA polymerase II” (Figure 4J). We asked whether Ddx21 coordinates transcription of hematopoiesis‐related genes. From RNA‐seq, we found diminished expression of key hematopoietic or erythropoietic genes *cKit*, *Gata1*, and *EpoR* (Figure 4K). Flow cytometry analysis confirmed the downregulation of cKit protein (Figure S6C), which is a critical stem cell marker for regulating HSPC proliferation [29]. Interestingly, we also found decreased expression of DBA‐related genes such as *Rps26* and *Scl25a38* (Figure 4K). To test whether the downregulation of hematopoietic and ribosomal genes is due to transcriptional inhibition, we measured nascent RNA synthesis by nuclear run‐on (NRO) 5‐ethynyluridine (EU) incorporation assay in cultured LK cells. EU‐NRO‐qPCR revealed a consistent downregulation of nascent RNA synthesis for *cKit*, *Gata1*, *Rps29*, and pre‐rRNA (but not *Gapdh*) in the *Ddx21^cKO^* cells (Figure 4L).

### 2.8 Ddx21 Interacts With Histone Modification Enzyme Kdm5a

Although p53 inhibition could rescue apoptosis in Ddx21‐deficient HSPCs, we found that it failed to rescue rRNA expression and hematopoietic colony formation. The reason for this intriguing phenomenon is unclear. To dissect the molecular network with which DDX21 interacts, we performed BioID‐MS proteomic profiling in K562 cells to identify proteins interacting with DDX21 (Figure 5A and Figure S7A–C). DDX21 was found to interact with different categories of proteins involved in ribonucleoprotein complex biogenesis, ribosome biogenesis, rRNA metabolic process, and RNA splicing, consistent with previously reported [16, 20, 27, 30] (Figure 5B). Interestingly, we found a group of histone‐binding proteins enriched in the DDX21 interactome, such as H3K4me3 demethylase KDM5A and histone deacetylases HDAC1/2 (Figure 5C and Figure S7D). Knockout of *Ddx21* in mouse HSPC had minimal effect on Kdm5a and Hdac1 protein levels (Figure S8A). Forward and reverse co‐immunoprecipitation (co‐IP) experiments demonstrated the interactions of DDX21 with KDM5A, HDAC1/2, but not SIN3A (Figure 5D–G and Figure S8C–F). Disruption of the ATPase/helicase activity of DDX21 did not abolish its interaction with KDM5A and HDAC1, as both helicase‐dead (DDX21^DEV^) and ATPase‐dead (DDX21^DAT^ and DDX21^SAT^) DDX21 mutants could still co‐immunoprecipitate both proteins (Figure 5H). Additionally, endogenous co‐IP revealed that DDX21 immunoprecipitates were highly enriched for KDM5A, whereas HDAC1 and SIN3A were substantially lower (Figure 5I). To confirm direct protein‐protein interactions independent of cellular context, we performed pulldown assays with purified recombinant proteins (Figure S8G). The results showed that DDX21 directly binds KDM5A and HDAC1 (Figure 5J). Furthermore, we investigated in situ protein‐protein interactions in freshly isolated mouse LK cells by proximity ligation assay (PLA). We observed a robust PLA signal for Ddx21‐Kdm5a. In contrast, the signals for Ddx21‐Hdac1/2 were considerably weaker, and the Ddx21‐Sin3a interaction was undetectable (Figure 5K and Figure S8H). Therefore, we focused our study on KDM5A.

**FIGURE 5:**
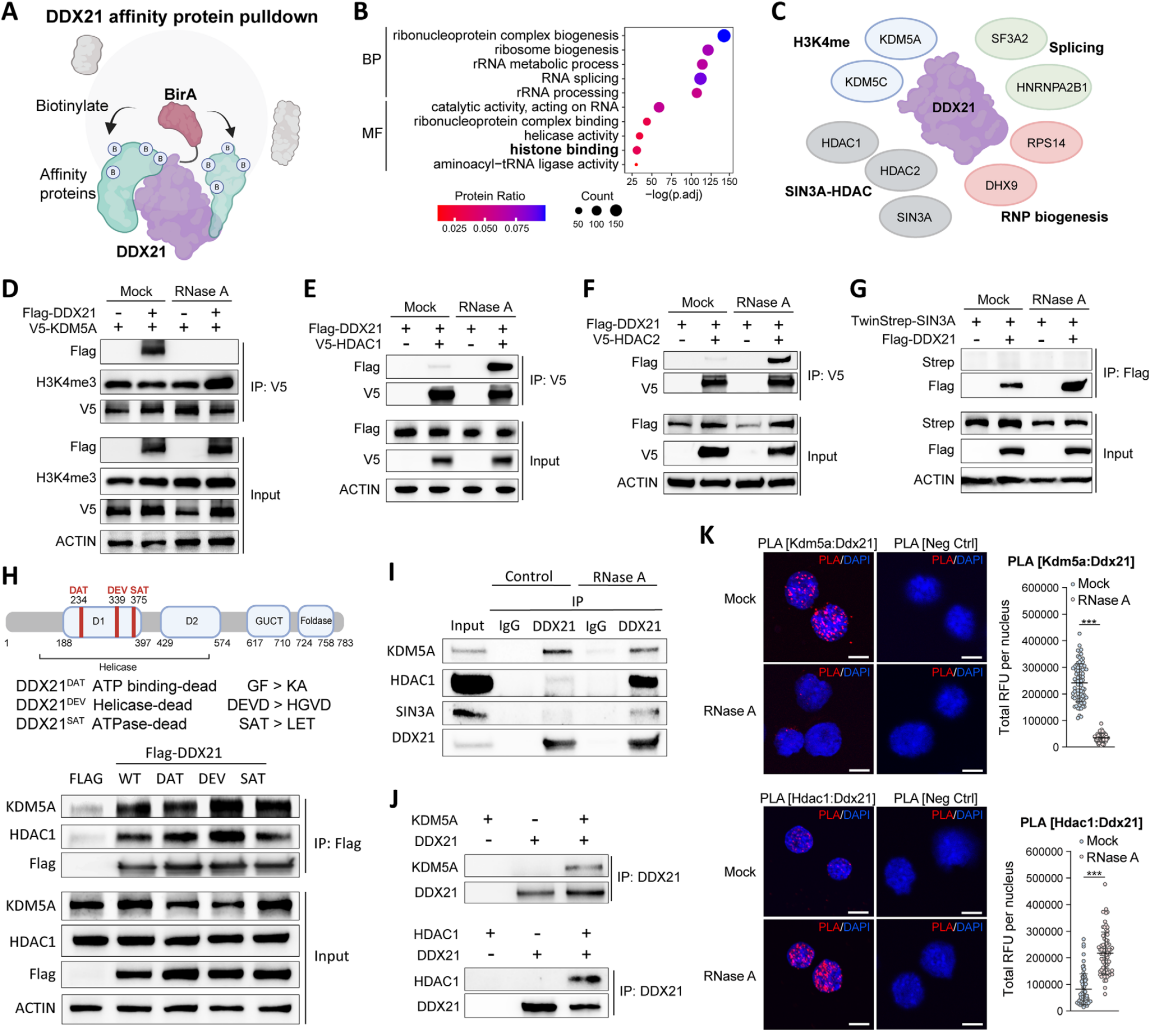
DDX21 interacts with histone demethylase KDM5A in an RNA‐dependent manner. (A‐C) Analysis of DDX21 proximity protein interactome in K562 cells overexpressed with DDX21‐BirA fusion protein. Biotinylated proteins were detected by mass spectrometry (n = 3). Proteins biotinylated by DDX21‐BirA were compared with control NLS‐BirA to obtain background subtracted interactome. (A) Schematic diagram of the BioID assay. (B) Top 5 enriched GO terms of biological processes (BP) and molecular function (MF) of the interactome. (C) Proteins related to histone modifications were enriched in the interactome. (D‐G) Co‐immunoprecipitation (Co‐IP) experiments in 293T overexpressing tagged‐proteins or tag‐only vector control (‐). (D) Co‐IP of H3K4me3 and Flag‐DDX21 by V5‐KDM5A. (E) Co‐IP of Flag‐DDX21 by V5‐HDAC1. (F) Co‐IP of Flag‐DDX21 by V5‐HDAC2. (G) Co‐IP of Twin‐strep‐SIN3A by Flag‐DDX21. (H) Co‐IP of endogenous KDM5A and HDAC1 by exogenous Flag‐DDX21 mutants in 293F. (I) Co‐IP of endogenous KDM5A, HDAC1, and SIN3A by endogenous DDX21 in K562 cells. (J) Equimolar recombinant protein pulldown assay of KDM5A or HDAC1 by DDX21. (K) Proximity ligation assay (PLA) staining between Ddx21 and Kdm5a or Hdac1 in mock or RNase A‐treated E13.5 fetal liver LK cells. Scale bar = 5 µm (n = 65‐95). ^*^ *p* < 0.05, ^**^ *p* < 0.01, ^***^ *p* < 0.001.

We next examined the effect of depleting RNA on the protein‐protein interactions. RNase A treatment did not change the protein expression of Ddx21 and its interaction partners (Figure S8B). However, depletion of RNA reduced Ddx21‐Kdm5a PLA signal (Figure 5K), consistent with their inability to co‐immunoprecipitate in the absence of RNA (Figure 5D and Figure S8C). Given that Ddx21 is predominantly recruited to RNA‐rich chromatin (e.g., nucleoli) under normal conditions, removal of chromatin‐associated RNA may disrupt Ddx21‐Kdm5a complex formation. Accordingly, RNA depletion enhanced KDM5A binding to H3K4me3 (Figure 5D). From these results, we propose a model in which chromatin‐associated RNA promotes Ddx21‐mediated recruitment and complex Kdm5a at active chromatin regions, thereby limiting Kdm5a access to H3K4me3. Upon RNA depletion, this interaction is impaired, favoring Kdm5a binding to H3K4me3‐marked sites.

### 2.9 Ddx21 And Kdm5a Co‐Occupy Active Promoters

To test the above hypothesis, we performed CUT&Tag to map the genomic binding sites of Ddx21 and Kdm5a, and the associated epigenetic marks for active (H3K4me3), repressive (H3K27me3), and enhancer‐associated (H3K27ac, H3K4me1) chromatin in fetal HSPC. Despite being an epigenetic repressor, global occupancy of Kdm5a is highly correlated with open chromatin (ATAC, R = 0.9) and active marks (H3K4me3, R = 0.64), but not repressive chromatin (H3K27me3, R = 0.13) (Figure 6A). Interestingly, Ddx21‐bound peaks highly overlapped with Kdm5a peaks (Figure 6B). Most H3K4me3^+^ peaks were co‐occupied by both Ddx21 and Kdm5a, and are predominantly located in promoters (82% of Ddx21^+^ Kdm5a^+^ H3K4me3^+^ peaks are promoter regions) (Figure 6C). By contrast, Ddx21 and Kdm5a co‐occupied regions without H3K4me3 were relatively scarce and are largely non‐promoters. H3K4me3^+^ regions with either Kdm5a or Ddx21 binding were also minor, highlighting the high co‐occurrence of Kdm5a and Ddx21 in promoters. On this basis, we focused our subsequent analyses on promoter regions. The promoter‐associated Kdm5a peaks were highly correlated with Ddx21 (R = 0.85), H3K4me3 (R = 0.91), and RNA level (R = 0.72) (Figure 6D). These analyses suggest that Ddx21 and Kdm5a co‐occupy active promoters and potentially modulate their transcription.

**FIGURE 6:**
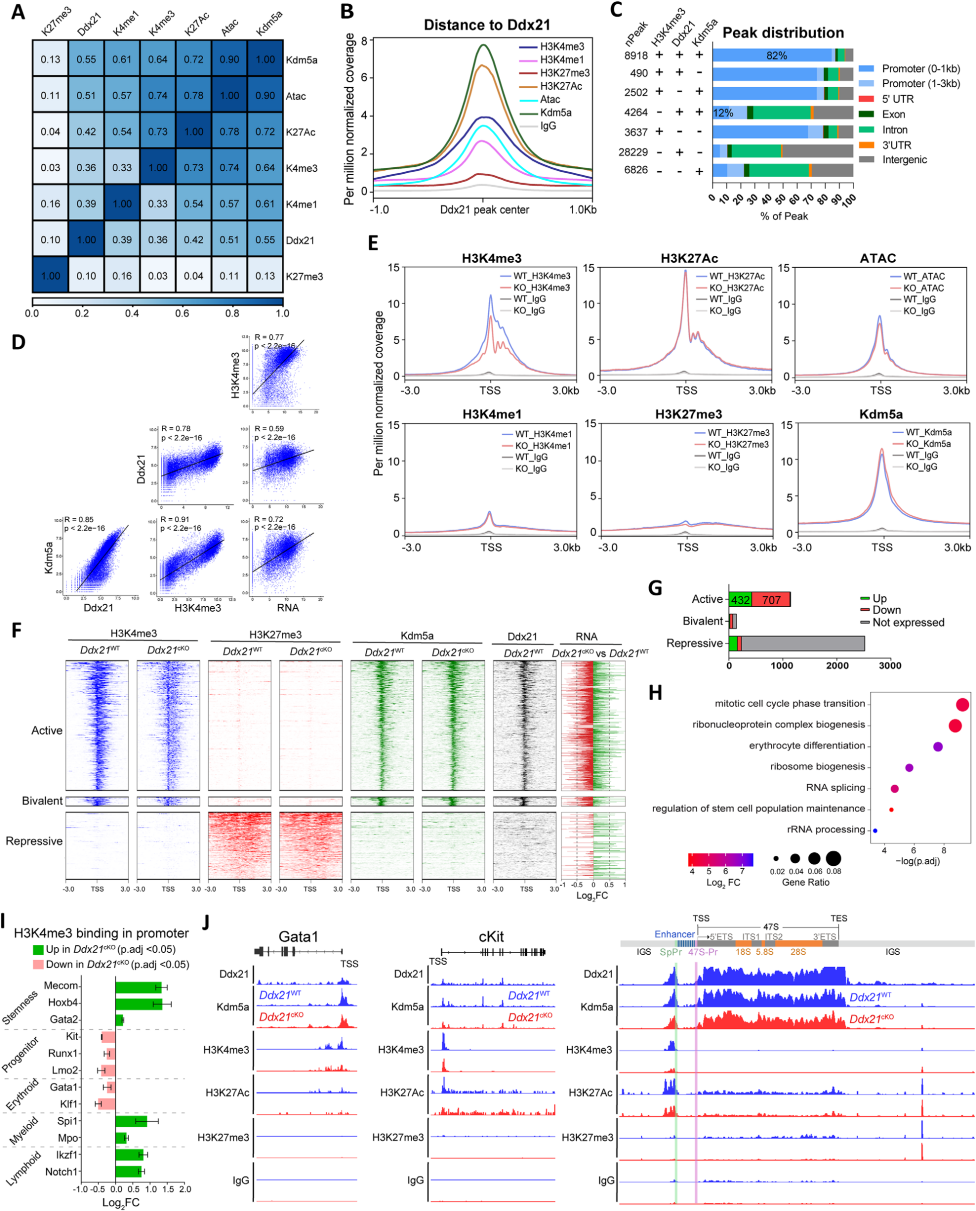
Ddx21 and Kdm5a co‐occupy active promoters to preserve a transcriptionally active state. Epigenetic profiling and analysis of H3K4me3, H3K27me3, H3K4me1, H3K27Ac, Ddx21, and Kdm5a by CUT&Tag‐seq, and open chromatin by ATAC‐seq in E13.5 fetal liver Lin^–^cKit^+^ (LK) cells of *Ddx21* ^*WT*^ and *Ddx21^cKO^* embryos (n = 2, biological replicates). Signals of the same target were normalized, scaled to per‐million, and merged between replicates. RNA expression levels were extracted from the E13.5 fetal liver LK cell transcriptome (n = 5). Promoters were defined as TSS±1500 bp. (A) Pearson correlation of all signals (bigWig) between histone marks, Ddx21, Kdm5a, and ATAC in *Ddx21^WT^* cells. (B) Profile plot of histone marks, Kdm5a, and ATAC signal relative to Ddx21 binding in *Ddx21^WT^* cells. (C) Peak number (nPeak) and distribution of H3K4me3, Ddx21, and Kdm5a co‐binding peaks in *Ddx21* ^*WT*^ cells. (D) Pearson correlation of log_2_(normalized counts+1) in all promoters of *Ddx21* ^*WT*^ cells between Kdm5a, Ddx21, H3K4me3, and RNA. (E) Profile plots of H3K4me3, H3K4me1, H3K27me3, H3K27Ac, Kdm5a, and ATAC signals along with IgG at TSS±3 kb, comparing *Ddx21^WT^* and *Ddx21^cKO^* cells. (F) Heatmap of H3K4me3, H3K27me3, Kdm5a and Ddx21 in active (H3K4me3^+^, H3K27me3^–^), bivalent (H3K4me3^+^, H3K27me3^+^) and repressive (H3K4me3^–^, H3K27me3^+^) promoters in *Ddx21* ^*WT*^ and *Ddx21^cKO^* cells. Differential gene expression from the transcriptome (RNA) was also shown. (G) Number of up‐regulated, down‐regulated, and non‐expressed genes in the active, bivalent, and repressive promoters using a log_2_FC > 0.5 cutoff. (H) GO enrichment of significantly downregulated genes (log_2_FC < 0.5, *p.adj* < 0.05) in *Ddx21^cKO^* from the active promoter cluster. (I) Significant differential H3K4me3 binding (*p.adj* < 0.05) in stemness, progenitor, erythroid, myeloid, and lymphoid marker gene promoters calculated by DiffBind. (J) Ddx21, Kdm5a, and histone mark occupancy at *Gata1*, *cKit*, and rDNA tandem repeat. IGS: intergenic spacer. SpPr: spacer promoter. 47S‐Pr: 47S rRNA promoter. ETS: external transcribed spacer. ITS: internal transcribed spacer. TSS: transcription start site. TES: transcription end site.

### 2.10 Reduction of TSS H3K4me3 Mark and Gene Expression Upon Ddx21 Loss

The interaction between Ddx21 and Kdm5a and their co‐occupancy at active promoters suggest an unexpected role for Ddx21 in coordinating epigenetic regulation. To delineate the epigenetic alterations following *Ddx21* knockout, we profiled the epigenetic landscapes in *Ddx21* ^*cKO*^ HSPC. As predicted, we observed significant downregulation of H3K4me3 near the transcription start site (TSS) following *Ddx21* knockout (Figure 6E and Figure S9), whereas enhancer marks H3K27ac and H3K4me1 remained largely unchanged. Surprisingly, Ddx21 loss resulted in only minimal changes to Kdm5a occupancy at TSS (Figure 6E). These observations indicate that DDX21 does not control KDM5A recruitment but rather restrains its demethylase activity while both proteins remain co‐bound at active promoters.

Indeed, Kdm5a and Ddx21 predominantly occupy the TSS of active (H3K4me3^+^) and bivalent (H3K4me3^+^ H3K27me3^+^) genes, but not repressive (H3K27me3^+^) promoters (Figure 6F). In this “active” gene cluster, *Ddx21* knockout resulted in more downregulated genes compared with upregulated genes, concordant with the decrease in H3K4me3 (Figure 6F,G). Intriguingly, we also observed a striking enrichment of both Kdm5a and Ddx21 at active enhancers (H3K4me1^+^ H3K27ac^mid/high^ ATAC^mid/high^), but not at repressive enhancers (H3K4me1^+^ H3K27Ac^low^ H3K27me3^+^), raising compelling questions regarding their role in regulating enhancer activity (Figure S10).

### 2.11 Kdm5a And Ddx21 Co‐Occupy Gata1, cKit and rDNA Promoters

Analysis of Ddx21/Kdm5a co‐occupied genes that are downregulated upon Ddx21 loss revealed biological processes (BPs) typically regulated by DDX21, such as RNP complex biogenesis, chromosome segregation, RNA splicing, and ribosome biogenesis. Notably, we also found enrichment of BPs “stem cell population maintenance” and “erythrocyte differentiation”. We next asked whether the downregulated hematopoietic progenitor genes, such as *Gata1* and *cKit*, are associated with epigenetic changes at their TSS/promoter regions. We found overlapping peaks of Ddx21 and Kdm5a near the TSS and promoter regions of *Gata1* and *cKit* (Figure 6J, left/middle panel). In line with the genome‐wide analysis, Ddx21 loss did not compromise Kdm5a occupancy, but decreased H3K4me3 in these regulatory regions (Figure 6I). The change in H3K4me3 correlates with their transcriptional downregulation (Figure 4K). By contrast, genes that specify HSC stemness (*Mecom*, *Hoxb4*, *Gata2*), myeloid (*Spi1*, *Mpo*) and lymphoid (*Ikzf1*, *Notch1*) fates generally had their promoter H3K4me3 upregulated (Figure 6I and Figure S11). Similar to *Gata1* and *cKit*, H3K4me3 at the rDNA spacer promoter (SpPr) was decreased (Figure 6J, right panel). In these example genes with upregulated promoter H3K4me3, Kdm5a occupancy was not significantly changed. We speculate that the presence of Ddx21 at active genes may hinder Kdm5a from directly catalyzing H3K4me3, despite their co‐recruitment to the promoters. To test this possibility, we next identify a truncated DDX21 protein that disables DDX21‐KDM5A complex formation.

### 2.12 DDX21 D2 Helicase Domain Is Required for KDM5A‐DDX21 Complex Formation

The DDX21 protein is composed of an N‐terminal domain, a C‐terminal domain, a GUCT domain, and two RecA‐like domains (D1 and D2) that form the helicase core. As the C‐ and N‐terminal domains are required for nuclear localization [27], truncation mutants deleting these regions were not constructed. Instead, we generated DDX21 truncation constructs lacking the D1 (DDX21^∆D1^), D2 (DDX21^∆D2^), or GUCT (DDX21^∆G^) domain (Figure 7A). Co‐IP experiments indicated that KDM5A‐DDX21 interaction was abolished in DDX21^∆D2^, but not in DDX21^∆D1^ or DDX21^∆G^ (Figure 7B). DDX21^∆D2^ also failed to co‐immunoprecipitate endogenous KDM5A protein (Figure 7C), further confirming the requirement of the D2 domain for KDM5A‐DDX21 complex formation. Additionally, we modeled protein‐protein interaction using AlphaFold3 [31]. We found that the DDX21 protein can dock into the groove of the crescent‐like KDM5A structure, where the D2 domain of DDX21 has close contact with the KDM5A PHD3 domain (Figure 7D), which is known to bind trimethylated H3K4 [32]. To test whether the co‐recruitment of DDX21 and KDM5A to chromatin requires a functional interaction involving the D2 domain, we performed ChIP‐reChIP in K562 cells with endogenous DDX21 depleted by shRNA. We first immunoprecipitated ectopically expressed Flag‐DDX21 (full‐length) or Flag‐DDX21^∆D2^, followed by a second ChIP against V5‐KDM5A. ChIP‐reChIP experiments showed that the amount of V5‐KDM5A recovered after sequential IP was substantially lower when Flag‐DDX21ΔD2 was used compared with full‐length Flag‐DDX21 (Figure 7E). Although this result is consistent with the reduced protein–protein interaction of the ΔD2 mutant (Figure 7B), we note that sequential ChIP cannot fully discriminate between loss of interaction and loss of co‐occupancy. Importantly, the first‐round Flag ChIP indicated that both full‐length DDX21 and DDX21^ΔD2^ occupy the tested promoters at similar levels, indicating that the mutant is not generally defective in chromatin binding.

**FIGURE 7:**
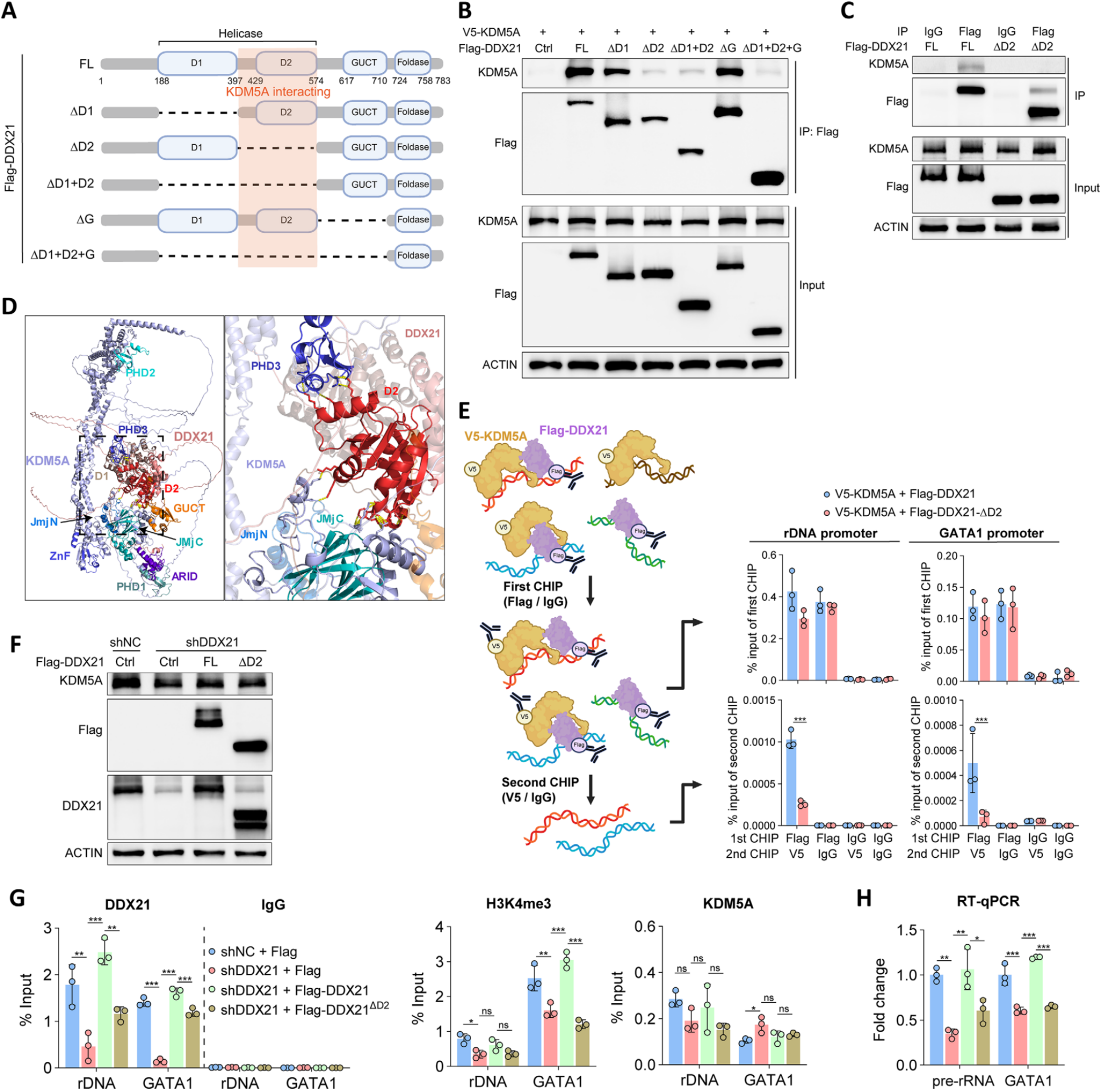
Recruitment of DDX21‐KDM5A complex at rDNA and *GATA1* promoters requires D2 helicase domain. (A) Schematic diagram of various DDX21 truncation constructs. FL: full‐length DDX21. (B) Co‐IP of exogenous V5‐KDM5A by exogenous truncated Flag‐DDX21 in 293T. (C) Co‐IP of endogenous KDM5A by exogenous full‐length or truncated Flag‐DDX21 in K562. (D) AlphaFold model prediction of DDX21‐KDM5A complex. PHD1, PHD2, PHD3, ZnF, JmjN, JmjC, and ARID domains of KDM5A, and D1, D2, and GUCT domains of DDX21 were colored. The right panel highlights bonds < 3.5Å between KDM5A and the D2 domain of DDX21. (E) ChIP‐reChIP qPCR analysis on rDNA and the *GATA1* promoter of K562 cells after endogenous DDX21 knockdown by shDDX21‐3 and overexpression of V5‐KDM5A and Flag‐DDX21‐FL/∆D2. (F‐H) ChIP‐qPCR and RT‐qPCR analysis of K562 cells after endogenous DDX21 knockdown by shDDX21‐3 and DDX21‐FL/∆D2 overexpression. (F) Western blot of total DDX21, overexpressed Flag‐DDX21, and endogenous KDM5A. Ctrl cells express the tag‐only empty construct. (G) DDX21, H3K4me3, KDM5A, and IgG ChIP analysis on rDNA and *GATA1* promoters. (H) RT‐qPCR analysis of pre‐rRNA and GATA1 mRNA expression. ^*^ *p* < 0.05, ^**^ *p* < 0.01, ^***^ *p* < 0.001.

We next performed a functional rescue experiment in which either DDX21 or DDX21^∆D2^ was expressed in K562 cells with endogenous DDX21‐depleted (Figure 7F). ChIP‐qPCR reveals restored H3K4me3 levels at *GATA1* and rDNA promoters in cells rescued by full‐length DDX21 but not DDX21^∆D2^ (Figure 7G). As a result of regaining H3K4me3, the mRNA of GATA1 and pre‐rRNA returned to a level comparable to non‐DDX21‐depleted control cells (Figure 7H). Notably, KDM5A occupancy at these promoters was not significantly changed between the DDX21 and DDX21^∆D2^ rescues (Figure 7G, right panel). Together with the ChIP‐reChIP result, these findings indicate that the DDX21 D2 domain is required for formation of a stable, catalytically inhibitory chromatin‐associated complex with KDM5A.

### 2.13 Ddx21 and Kdm5a Regulate rDNA and cKit

To test whether the demethylation activity of KDM5A modulates rDNA transcription, we treated K562 cells with KDM5A inhibitor CPI‐455. As expected, CPI‐455 treatment increased global H3K4me3 levels, accompanied by elevated rRNA levels and translational activity in DDX21‐knockdown cells (Figure S12A–C). Guided by these results, we tested whether CPI‐455 could rescue cellular defects in mouse HSPCs. The results indicated that CPI‐455 alone could also increase global H3K4me3 levels and enhance protein synthesis (Figure 8A and Figure S12F), but failed to suppress apoptosis and cell cycle arrest, in agreement with K562 results (Figure 8B,C and Figure S12D,E). As apoptosis and cell cycle arrest are p53‐dependent, we tested whether combined inhibition of p53 and Kdm5a had a beneficial effect. Remarkably, co‐treatment with CPI‐455 and PFT‐α showed a cooperative effect by further enhancing protein synthesis and, meanwhile, rescuing apoptosis and cell cycle arrest in Ddx21‐knockdown HSPC (Figure 8A–C). Next, we tested whether dual knockdown of Kdm5a and p53 could rescue the defect in HSPC colony formation (Figure S12G). By CFU assay, we found that Kdm5a knockdown alone could increase BFU‐E number, whereas dual knockdown of Kdm5a and p53 significantly increased BFU‐E but slightly increased CFU‐GM numbers (Figure 8D). Lastly, we investigated the effect of dual inhibition of Kdm5a and p53 on fetal HPSCs in vivo (Figure 8E). Injections of CPI‐455 or PFT‐α alone into pregnant mice at E11.5 & E12.5 did not increase expression of cKit or HSPC number in E13.5 FL cells by flow cytometry analysis. However, co‐injection of CPI‐455 and PFT‐α could increase cKit expression (Figure 8F). As a result, both LS^–^K and LSK HSPC populations were increased compared with vehicle control, although this temporal inhibition resulted in a partial rescue (Figure 8F). To assess whether dual inhibition of Kdm5a and p53 could rescue HSC repopulating potential, we performed a BMT assay using FL LK cells transduced with shRNAs against Kdm5a and p53 (shKdm5a‐shP53‐GFP) and Ddx21 (shDdx21‐Cherry) or scramble control (shNC‐Cherry) (Figure 8G). As expected, depletion of Ddx21 resulted in nearly null repopulation capacity (0% PB chimerism from 4 to 12 weeks following BMT) (Figure 8H). Dual knockdown of Kdm5a and p53 in Ddx21‐depleted cells could slightly rescue the repopulation defect (0.05%–0.15% PB chimerism) (Figure 8H,I). A low chimerism rate was also observed in Ddx21‐normal cells with dual Kdm5a and p53 knockdown (0.1%–0.3%). Collectively, these data proved a cooperative mode of DDX21 and KDM5A in the regulation of HSPC survival and proliferation.

**FIGURE 8:**
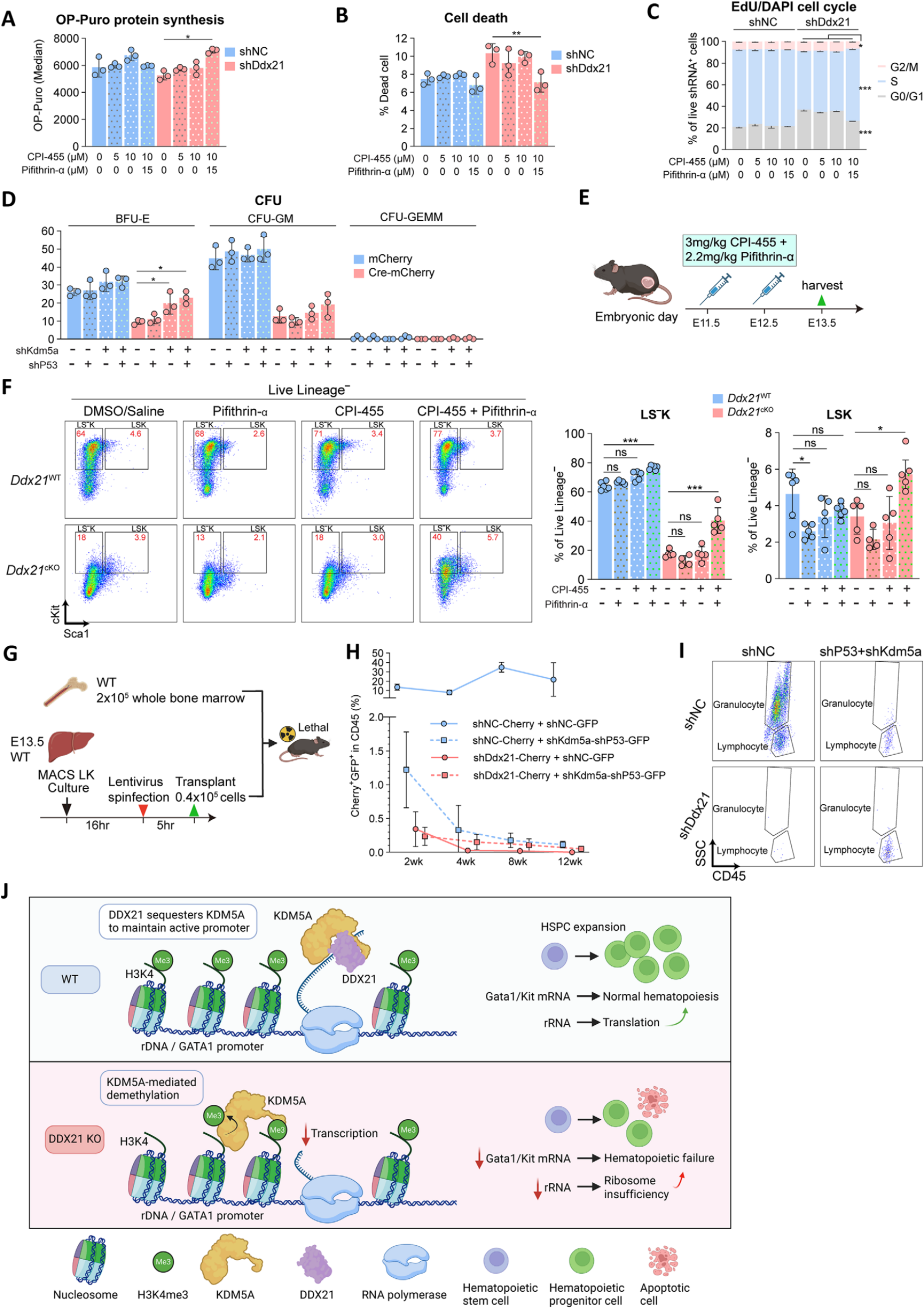
Dual inhibition of Kdm5a and p53 synergistically enhances survival and BFU‐E formation of DDX21‐deficient HSPCs. (A‐C) Flow cytometry analysis of O‐propargyl‐puromycin (OP‐Puro) nascent protein synthesis, apoptosis, and EdU/DAPI cell cycle of cultured E13.5 fetal liver LK cells after 3 days of knockdown and culture with 15 µM PFT‐α/5–10 µM CPI‐455/DMSO (n = 3). (A) Median fluorescence of OP‐Puro in live shRNA^+^ cells after 30 min of OP‐Puro incorporation. (B) Percentage of dead or pre‐apoptotic shRNA^+^ cells determined by Apotracker and fixable viability dye staining. (C) EdU/DAPI cell cycle analysis of live shRNA^+^ cells after 1hr of EdU incorporation. (D) CFU colony formation assay (Methocult^TM^ M3434) of cultured E13.5 *Ddx21* ^*f/f*^ fetal liver LK cells after 3 days of combinatorial Cre and shRNA‐mediated knockdown. Live shRNA^+^ cells were FACS‐sorted for CFU plating (n = 3). (E‐F) In vivo rescue of *Ddx21^cKO^* HSPC by injection of pifithrin‐α (PFT‐α) and/or CPI‐455 into pregnant mice carrying *Ddx21^WT^* and *Ddx21^cKO^* embryos. Control mice were injected with an equivalent DMSO/saline mix (n = 5‐6). (E) Schematic illustration of the injection. (F) Flow cytometry analysis of HSPC populations in live Lin^–^ fetal liver cells after injection. The number in flow gates represents the average percentage of the parent gate. (G‐I) In vivo rescue of transplanted shDdx21 fetal liver cells after shKdm5a and shP53 knockdown. (G) Schematic diagram of the transplantation. E13.5 WT fetal liver cells were cultured briefly for activation, followed by lentiviral spinfection, incubation for 5 hrs, and transplanted immediately afterward (n = 5). (H) Peripheral blood CD45 chimerism (mCherry^+^ and GFP^+^ from 2 weeks to 12 weeks post‐transplantation. (I) Flow cytometry plot (SSC/CD45) of peripheral blood leukocytes at 12 weeks post‐transplantation. (J) Graphic illustration of the DDX21‐KDM5A model in safeguarding hematopoietic expansion by regulating epigenetic dynamics to maintain ribosomal and hematopoietic gene output. ^*^ *p* < 0.05, ^**^ *p* < 0.01, ^***^ *p* < 0.001.

To summarize, our work demonstrated a novel non‐canonical function of DDX21 in the epigenetic regulation of ribosome biogenesis and hematopoiesis during fetal development. Mechanistically, DDX21 counteracts KDM5A to maintain an active epigenetic state for the transcription of hematopoietic genes (e.g., *cKit* and *Gata1*) and rDNA, the perturbation of which triggers ribosomal stress and impairs HSPC expansion, leading to hematopoietic failure (Figure 8J).

## 3 Discussion

Our study unveils an epigenetic role of RNA helicase DDX21 in safeguarding fetal hematopoiesis by interacting with histone modification enzyme KDM5A. We demonstrate that Ddx21 loss results in downregulation of H3K4me3 associated with active promoters, including key hematopoietic genes *Gata1* and *cKit*, and rDNA. Impaired transcription of rDNA and RP genes aligns with a canonical role of DDX21 in regulating ribosome biogenesis and proteostasis [14, 15, 19, 33]. Importantly, we identify a mechanism whereby Ddx21 counteracts Kdm5a to maintain an active chromatin state at ribosomal and hematopoietic genes, ensuring robust transcriptional output necessary for HSPC expansion. Our data suggest that DDX21 antagonizes KDM5A catalytic activity at active promoters without altering KDM5A occupancy. Structural modeling indicates that the D2 domain of DDX21 contacts the PHD3 reader domain of KDM5A, potentially impairing productive engagement with H3K4me3. Consequently, loss of DDX21 permits co‐bound KDM5A to demethylate H3K4me3 more efficiently, resulting in transcriptional repression of ribosomal and hematopoietic genes. This activity‐inhibition model reconciles the reduction in H3K4me3 with the unchanged KDM5A chromatin occupancy observed upon DDX21 depletion.

Intriguingly, KDM5A is generally regarded as a transcriptional repressor [34, 35, 36]. The co‐occupancy of Ddx21 and Kdm5a at active promoters appears enigmatic, although previous studies consistently reported the recruitment of KDM5A to H3K4me3‐rich chromatin [37, 38]. KDM5A is a classical pRB‐binding protein that regulates the cell cycle during differentiation [39]. In proliferating cells, KDM5A is recruited to highly active genes to mediate their subsequent repression upon differentiation [37]. The identification of the DDX21‐KDM5A complex occupying actively transcribed genes in HSPC supports this model. Notably, the DDX21‐KDM5A interaction appears RNA‐dependent, as RNase treatment impaired DDX21‐KDM5A interaction, suggesting that RNA scaffolds, e.g., transcription‐associated R‐loops or nascent transcripts, may stabilize their association at active chromatin. Profiling of Ddx21‐associated chromatin RNA may help to address this question. While the function of DDX21 in coordinating rDNA transcription has been reported [14, 40], the specific role of KDM5A in the epigenetic control of ribosome biogenesis was not known before, although other members of the Jumonji family (e.g., KDM2A/B) have been shown to repress rDNA transcription [41, 42]. Our data suggest that DDX21 may antagonize KDM5A at active promoters, impeding its demethylase activity and thereby preserving a high H3K4me3 level to favor active transcription. This model may explain why actively dividing progenitors, which require high rRNA and protein biosynthesis, are particularly sensitive to DDX21 loss. In the absence of Ddx21, many Kdm5a‐occupied genes show decreased expression, which is concordant with a diminished H3K4me3 level, possibly due to Kdm5a‐mediated epigenetic repression. In hematopoietic progenitors, Ddx21 loss also affects a set of hematopoietic genes, including *cKit* and *Gata1*, whose downregulation is crucial for changing HSPC survival, proliferation, and erythroid progenitor differentiation [29, 43]. It is plausible that during terminal differentiation, downregulation or relocalization of DDX21 protein from the nucleoli shuts down the expression of HSPC genes and diminishes rDNA transcription, creating a transcriptional and metabolic state to drive differentiation. Therefore, future work may focus on dissecting the dynamic transcriptional and epigenetic programs during erythropoietic terminal differentiation, which was found to be defective in our study.

The link between defective ribosome biogenesis and hematopoietic failure has been well established in ribosomopathies such as DBA and the relevant animal models [44], which manifests as fetal or neonatal anemia due to low erythroid precursors [4, 5, 45]. Our work positions DDX21 as a key non‐structural regulator whose loss phenocopies ribosomopathy‐associated anemia, underscoring the vulnerability of HSPCs to perturbations in not only ribosomal genes but also a number of RNA helicases such as *DDX41* [12, 13], *DDX18* [46], and *DDX21*, as shown by this study. The observed p53 activation and the transcriptional inhibition of DBA‐associated genes (*Rps26*, *Rps29*, *Scl25a38*) in Ddx21‐deficient HSPC align with the canonical “ribosomal stress checkpoint” model, where ribosome insufficiency triggers p53‐mediated cell cycle arrest [44, 45, 47, 48]. A recent study demonstrated that Ddx21 is also essential for adult HSC maintenance, where its loss triggers nucleolar stress, impaired ribosome biogenesis, and p53‐dependent apoptosis and cell‐cycle arrest [21]. While both fetal and adult stages share the ribosomal stress–p53 axis, our findings reveal an additional, non‐canonical mechanism operative in fetal HSPCs: Ddx21 partners with Kdm5a to preserve H3K4me3 at both rDNA and key hematopoietic genes (*Gata1*, *cKit*). These observations indicate that DDX21 employs both shared (nucleolar stress/p53) and stage‐specific (epigenetic) mechanisms to safeguard hematopoiesis. Therapeutic implications emerge from the partial rescue of HSPC defects by targeting Kdm5a and p53 using preclinical inhibitors. While p53 blockade alleviates apoptosis and, as shown in zebrafish, can rescue morphological phenotypes associated with ribosomopathy but not erythroid defects [49, 50], inhibition of p53 or Kdm5a alone fails to restore cKit expression or HSPC numbers in *Ddx21^cKO^* fetuses. Only dual inhibition yields a partial recovery of progenitor populations. Enhancing ribosomal transcription via epigenetic inhibition of KDM5A may be necessary to more effectively ameliorate hematopoietic defects. Consistent with this, dual knockdown of p53 and Kdm5a produces only modest improvement in long‐term engraftment in transplantation assays, reflecting the essential genome‐wide functions of Kdm5a (homozygous deletion of Kdm5a is perinatal lethal). This aligns with clinical observations in DBA, where glucocorticoids improve anemia but fail to address ribosomal defects [51, 52]. Targeting the DDX21‐KDM5A complex could offer a complementary strategy. However, global inhibition has inherent limitations, and future approaches may require locus‐specific modulation of KDM5A activity at critical promoters to optimize therapeutic potential.

## 4 Conclusions

In conclusion, our findings identify DDX21‐KDM5A as a new complex that primes transcription of critical genes to sustain hematopoietic development. By coupling rRNA synthesis to chromatin modification, Ddx21 ensures that fetal HSPCs meet the extraordinary biosynthetic demands of the developing fetus, especially for the predominant erythroid lineage. Disruption of this complex not only illustrates the etiology of ribosomopathies but also opens avenues for therapies targeting the epigenetic‐ribosome interface.

## 5 Materials and Methods

### 5.1 Animals and Embryo Analysis

*Ddx21* ^*f/f*^ mice were custom‐generated by Cyagen with insertion of loxP sites into introns flanking exon 3–5, creating a nonsense mutation upon Cre‐mediated recombination. B6.Cg‐Commd10^Tg(Vav1‐icre)A2Kio^/J (*Vav1‐Cre*) (JAX #008610), B6.SJL‐Ptprc^a^ Pepc^b^/BoyJ (CD45.1) (JAX #002014) and B6.129(Cg)‐Gt(ROSA)26Sor^4(ACTB‐tdTomato,‐EGFP)Luo/J^ (*mTmG^f/f^*) (JAX #007676) mice were imported from the Jackson Laboratory. All mice were maintained on a C57BL/6 background. Mice were bred and housed in pathogen‐free animal facilities with a regular light‐dark cycle. Embryo was determined as day 0.5 when a vaginal plug was observed the following day after mating. Both male and female mice were included in the study and randomly assigned to experimental groups, except for male‐only transplantation recipients. Genotyping and Ddx21‐KO efficiency PCR were performed using RapidTaq (Vazyme). Primer sequences are listed in Table S3. All animal experimental protocols were reviewed and approved by the Animal Experimentation Ethics Committee of The Chinese University of Hong Kong (Approval number: 23‐249‐GRF).

### 5.2 Fetal Liver and Bone Marrow Cell Isolation

Isolated tissues and cells were kept either on ice or at 4°C throughout harvesting, centrifugation, and cell sorting. Dissected fetal livers were washed twice with PBS and kept in fetal liver buffer (2% FBS/1 mM EDTA/10 mM Glucose/PBS) on ice. Fetal livers were triturated about 20 times with a P1000 pipette. Cell suspension was strained with a sterile 70 µm cell strainer (Beyotime) and kept in fetal liver buffer for no longer than 1 h until subsequent experiments.

For bone marrow isolation, tibia and femur were harvested. Bones were stripped clean of connective tissues and muscles; epiphyses were removed at both ends. Bone marrow was flushed with staining buffer (1% FBS/PBS) with a 25G needle and filtered with a sterile 70 µm cell strainer. After centrifugation, cells were resuspended in 100 µL RBC lysis buffer (150 mM NH_4_Cl/10 mM NaHCO_3/_1 mM EDTA) for 2 min on ice and quenched with 500 µL staining buffer per bone. After centrifugation, cells were filtered again with a 70 µm strainer.

### 5.3 Cell Line Culture, Transfection and Lentivirus Transduction

All cells were maintained in a 37°C, 5% CO_2_ humidified incubator. 293T cells were cultured in high‐glucose DMEM (Life Technologies) supplemented with 10% FBS, 1x NEAA, 2 mM glutamine, 1 mM sodium pyruvate, and 1x Antibiotic‐Antimycotic. K562 cells were cultured in IMDM (Life Technologies) supplemented with 10% FBS, 1x NEAA, and 1x Antibiotic‐Antimycotic. Human embryonic stem cells (H1) were cultured in mTESR+ medium on Geltrex (Life Technologies, A1413302) coated wells. Medium was changed every 1–2 days. H1 was maintained at <60% confluency and passaged using Dispase, followed by scraping and 2–3 rounds of trituration. Expi293F (293F) cells were cultured in SMM 293‐TIL‐N complete medium (Sino Biological) and shaken in 250 mL flasks at 150 rpm, maintained at <5 × 10^6^ cells/ml. 293T and 293F transfection was performed using polyethylenimine (PEI) with a 1:2.5 (DNA:PEI) ratio (ug). Full medium change was performed on the following day. K562 cell lentiviral transduction was performed at MOI = 5 with 8 µg/mL polybrene and incubated overnight. Full medium change was performed on the following day.

### 5.4 Fetal Liver HSPC Cell Culture

Live LK cells FACS‐sorted from fetal liver were cultured in StemPro‐34 SFM medium (Gibco) with SCF (100 ng/mL), FLT3L (50 ng/mL), TPO (50 ng/mL), IL3 (10 ng/mL), IL6 (10 ng/mL), L‐glutamine (1x), and Anti‐Anti (1x), and maintained at a density of <0.2 × 10^6^ cells/ml. Lentiviral transduction was performed on day 1, where each type of lentivirus was added at MOI = 10 with 8 µg/mL polybrene. Cells were centrifuged at 1000 g at 37°C for 1hr and incubated for 5hr in a culture hood. A full medium change was performed afterward with addition of drug/DMSO as indicated. Control lentivirus (shNC‐GFP/mCherry or mCherry‐only) was added to respective samples to ensure equivalent total MOI across all conditions. After 3 days of transduction, live shRNA+ cells were FACS‐sorted and analyzed.

### 5.5 Differentiation of CD34+ HSPC from hESC

H1 hESC was differentiated into HSPC using STEMdiff Hematopoietic Kit (STEMCELL Technologies #5310) according to the manufacturer's instructions. On day 10, 1 × 10^6^ functional titre of shRNA‐mCherry lentivirus was added to each 12‐well with 8 µg/ml polybrene and incubated for 5 h. A full medium change was performed afterward, with addition of drug or DMSO as required. After 2 days, HSPC (live CD45^+^ CD34^+^ CD43^+^ shRNA‐mCherry^+^) were FACS‐sorted or analyzed.

### 5.6 Lentiviral Packaging, Purification, and Titre Determination

Second‐generation lentivirus was generated as previously described with some modifications [53]. In brief, 22 × 10^6^ 293T cells in a 15 cm dish were transfected with 55 µg of plasmids (pMD2.G, psPAX2, and transfer plasmid in 1:1:1.5 molar ratio) and 140 µg PEI (1:2.5). Medium was replaced on day 1, and viral medium was collected on day‐2/3. Cell debris was removed by low‐speed centrifugation (1000 g x 5 min, 4°C), followed by filtering the supernatant through a 0.45 µm PES filter. Virus was concentrated by high‐speed centrifugation (20 000 g x 2hr, 4°C). The viral pellet was resuspended in DMEM, aliquoted, and kept at ‐80°C. Cre‐IRES‐mCherry lentivirus was purchased from BrainVTA. Functional lentiviral titre was calculated by serial transduction of 293T cells in 8ug/ml polybrene and calculating the percentage of fluorescence‐positive cells after 3 days of transduction. shRNA sequences were listed in Table S4.

### 5.7 Flow Cytometry and Cell Sorting

All antibody staining was carried out in staining buffer (1% FBS/PBS) for 30 min at 4°C. Cell suspension was centrifuged at 500 g x 5 min at 4°C. Each reaction consisted of 1–5 × 10^6^ cells per 50 µL staining solution. Antibody details, panels, and dilutions were listed in Supplementary Table S5 and Table S6. Flow cytometry was performed on either BD Symphony A5 SE or BD FACSDiscovery S8. Analysis was performed using FlowJo v10.8.1. Cell sorting was performed with either BD FACSDiscovery S8 or BD FACSAria Fusion in purity mode. Samples and collection tubes were kept at 4°C. Gating strategies were illustrated in Figures S2 and S4A,B.

For fetal liver HSPC analysis, HSC isolation and analysis of transplanted bone marrow, 5 × 10^6^ cells were stained with biotin‐conjugated lineage antibodies (CD3, B220, CD11b, Gr1, Ter119) and viability dye. After washing with staining buffer, cells were stained with streptavidin, cKit, Sca1, CD48, CD150, CD16/32, and CD34 antibodies. For analysis of transplantation involving CD45.1 mice, CD45.1 and CD45.2 antibodies were also included. For erythroid cell analysis, Ter119 was omitted in the lineage panel, and cells were subsequently stained with CD71 and Ter119 antibodies. For leukocytes, cells were stained with viability dye, CD11b, Gr1, CD3, and B220 antibodies.

For sorting fetal liver LK cells, fetal liver cells were stained with cKit antibody and viability dye after lineage depletion. For peripheral blood analysis of transplanted mice, leukocytes were stained with CD3, B220, CD11b antibodies, viability dye, and CD45.1/CD45.2 or CD45 antibodies depending on the transplantation experiment. Human HSPCs were analyzed or sorted after staining with CD34, CD43, CD45 antibodies, and viability dye. For apoptosis analysis, Apotracker Green (BioLegend) was co‐stained with surface markers.

### 5.8 Colony formation assay (CFU)

Mouse hematopoietic colony formation assay was performed using MethoCult GF (STEMCELL, M3434) according to the manufacturer's instructions. Colonies were counted on day 12. For fresh fetal liver cells, 20000 cells were seeded in triplicate. For lentiviral‐transduced fetal liver cells, FACS‐sorted LK cells were cultured and transduced as mentioned above. 6000 live transduced cells were FACS‐sorted and seeded in duplicates. A human hematopoietic colony formation assay was performed using MethoCult GF (STEMCELL, H4536). Colonies were counted on day 16. 3000 FACS‐sorted, lentiviral‐transduced HSPCs were seeded in duplicates.

### 5.9 Transplantation

All recipient mice were C57BL/6 males aged 2–3 months old in either WT or CD45.1 background and kept in IVC cages. They were fed 0.5 mg/ml enrofloxacin added to drinking water for 3 days prior to transplantation and 14 days after transplantation. For complete myeloablation, recipient mice were irradiated with 10 Gy gamma irradiation (total body) using a Gammacell 3000 irradiator with a 137Cs radiation source on the same day of transplantation. For each recipient, 200 µL cell suspension in HBSS was transplanted via tail vein injection using a 30G needle. The time between irradiation and injection was kept within 3–6 h. All bone marrow cells were RBC‐lysed. For fetal liver transplantation, the cell ratio between E13.5 fetal liver and bone marrow cells is 5:1. For fetal liver HSC transplantation, cell ratio between E13.5 fetal liver HSC to bone marrow is 1:3000.

For limiting dilution HSC transplantation assay, surviving mouse at 16 weeks post‐transplantation with >1% PB reconstitution in all 3 lineages (T, B and granulocytes) is considered a positive transplantation. HSC frequency was calculated using Extreme Limiting Dilution Analysis (ELDA) based on positive transplantations [54].

For transplantation of lentivirus‐transduced fetal liver LK cells, they were first MACS‐isolated, cultured, and lentivirally transduced as stated above. After 5 hr of lentiviral incubation. 0.4 × 10^5^ cells were mixed with 2 × 10^5^ WT BM cells and transplanted as above. Cell ratio between cultured LK cells and bone marrow is 1:5.

### 5.10 EdU/DAPI Cell Cycle Flow Cytometry

5‐Ethynyl‐2'‐deoxyuridine (EdU)/4',6‐Diamidino‐2‐Phenylindole, Dihydrochloride (DAPI) cell cycle analysis was performed using Click‐iT Plus EdU Alexa Fluor 647 Flow Cytometry Assay Kit (Life Technologies) according to the product manual. Cells were incubated with 10 µM EdU under culture conditions for 1hr. Pregnant mice were injected i.p. with 30 µg/g EdU, and fetal liver was harvested 30 min post‐injection. Click‐iT reaction was performed after surface panel staining, fixation, and permeabilization. Cells were subsequently stained with 1 µg/ml DAPI for 30 min at 4°C. Events were captured in linear mode with a flow rate <1000 events/sec for all samples.

### 5.11 O‐Propargyl‐Puromycin (OP‐Puro) Nascent Protein Synthesis Flow Cytometry

Nascent protein synthesis was determined using Click‐iT Plus OPP Alexa Fluor 647 Protein Synthesis Assay Kit (Life Technologies) according to the product manual. Cells were incubated with 10 µM OP‐Puro for 30 min under culture conditions. Click‐iT reaction was performed after surface panel staining, fixation, and permeabilization. 1X Click‐iT saponin‐based permeabilization and wash reagent from Click‐iT Plus EdU Alexa Fluor 647 Flow Cytometry Assay Kit was used instead of the suggested Triton X‐100‐based permeabilization buffer.

### 5.12 5‐Ethynyluridine (EU) Nascent RNA Transcription

Cells were incubated in 10 µM 5‐ethynyluridine (EU) for 1 h under culture conditions. Total RNA was extracted using FastPure Cell/Tissue Total RNA Isolation Kit V2 (Vazyme). Click‐iT reaction was performed with 3 µg RNA and 0.25 mM Biotin‐PEG4‐Azide (Beyotime) in a 300 µL total volume for 30 min at RT using the BeyoClick kit (Beyotime). Total RNA after the click‐iT reaction was extracted using the same extraction kit, eluted with 30 µL H_2_O. 50 µL streptavidin bead (Beyotime) was washed twice with TBS, followed by 0.05 M NaCl (DEPC‐treated) twice and 0.1 M NaCl (DEPC‐treated) once. Beads were resuspended in 1x bind‐and‐wash buffer (5 mM Tris‐HCl pH7.5, 0.5 mM EDTA, 1 M NaCl, 0.01% Tween‐20) and incubated with the RNA eluate for 1hr at RT with end‐to‐end rotation. Beads were washed twice in 1x bind‐and‐wash buffer and resuspended in 40 µL PrimeScript RT Master Mix (Takara). On‐bead reverse transcription was carried out at 37°C for 1 h, followed by 85°C for 5 min to inactivate the enzyme and release the cDNA. For qPCR, cDNA was diluted 10 fold, and 4 µL was used per 10 µL qPCR reaction using TB Green Premix Ex Taq II (Takara).

### 5.13 Reactive Oxygen Species (ROS) Detection Flow Cytometry

Flow cytometry ROS quantification was performed by staining with ROS dye H_2_DCFDA (Life Technologies). In brief, cells were incubated in 50 µL 10 µM H_2_DCFDA/PBS at 37°C for 20 min after surface panel staining and washing. Cells were then diluted with 250 µL staining buffer and kept on ice. They were subjected to flow cytometry immediately. Signal was captured with the BP537/32 filter “GFP/FITC channel” of the BD FACS Symphony A5 SE with blue laser excitation.

### 5.14 Peripheral Blood Leukocyte Preparation and Staining

Tail vein peripheral blood was extracted by tail nicking with a 27G needle. 20 µL blood was mixed with 0.5 µL 0.5 M EDTA (pH 8.0) and incubated with 0.5 µL TruStain FcX (BioLegend) for 10 min. RBCs were lysed by adding 200 µL RBC lysis buffer (150 mM NH_4_Cl/10 mM NaHCO_3_/1 mM EDTA) for 10 min and quenched with 1 mL PBS. Leukocytes were centrifuged at 200 g x 5 min, and stained with a surface antibody panel and analyzed by flow cytometry for chimerism rate.

### 5.15 Hemoglobin Quantification Assay

Hemoglobin content in fetal liver was measured by Hemoglobin Assay Kit (Colorimetric) (Abcam ab234046) in duplicate according to the product manual. Each reaction consisted of 0.24 × 10^6^ total fetal liver cells resuspended in 20 µL ddH_2_O. Hemoglobin standards (Sigma H2500) were dissolved in water and diluted according to the manual for standard curve construction. Readout was measured at 575 nm using a plate reader.

### 5.16 CPI‐455 and Pifithrin‐α Injection

Stock CPI‐455 (25 mM, 6.96 µg/µL) was prepared by dissolving in DMSO. Stock Pifithrin‐α (PFT‐α) (10 mM, 3.67 µg/µL) was prepared in DMSO. Both CPI‐455 and PFT‐α were freshly diluted in physiological saline (0.9% NaCl) to a final injection volume (100 µL/mouse). CPI‐455 and PFT‐α dosages were 3 mg/kg and 2.2 mg/kg, respectively. Pregnant mice were injected intraperitoneally at E11.5 and E12.5, and fetal livers were harvested at E13.5. Control mice were injected with an equivalent injection volume and ratio of saline/DMSO.

### 5.17 Raising of Polyclonal Antibody Against Mouse Ddx21

Antibody targeting mouse Ddx21 was raised against the M719‐Q851 peptide of mouse Ddx21 (Q9JIK5). The peptide has low sequence similarity with other proteins, including other Ddx family members. Rabbit Ddx21 antibody was produced and purified by ABclonal. Specificity was tested by immunofluorescence staining in *Ddx21* ^*cKO*^ fetal liver LK cells (Supplementary Figure S1E).

### 5.18 Fetal Liver Cell Cytospin, RNase A Treatment, and Immunofluorescence

RNase A treatment of cells for immunofluorescence was performed as previously described with some modifications [55]. In brief, 2 × 10^4^ fetal liver LK cells were permeabilized with 0.05% Tween‐20/PBS for 10 min on ice, washed with 1 mL PBS, centrifuged, and resuspended with 10 µL of 1 mg/ml RNase A/PBS for 30 min at RT. Cell suspension was diluted to 100 µL with PBS and centrifuged at 40 g for 3 min in a cytospin centrifuge to prepare the cytospin slides. After brief drying, cells were fixed with 4% PFA/PBS for 10 min and washed. Blocking and permeabilization were performed with 0.5% Triton X‐100/10% goat serum/PBS for 30 min at RT. Primary antibody diluted in 0.1% Tween‐20%/5% goat serum/PBS was incubated at 4°C overnight. After 3 washes in PBS, respective secondary antibodies (1:1000) and 1 µg/ml Hoechst 33258 (Life Technologies) in 0.1% Tween‐20%/5% goat serum/PBS were applied for 1hr at RT. Slides were mounted with ProLong Glass Antifade Mountant (Life Technologies). Antibodies used were listed in Table S7.

### 5.19 Proximity Ligation Assay (PLA) Staining

PLA staining on the fetal liver LK cell cytospin slides was performed using the Duolink In Situ Orange Starter Kit Mouse/Rabbit kit (Sigma) according to the product manual. In brief, cytospin slides were fixed in 4% PFA for 10 min at RT and permeabilized in 0.1% Triton X‐100/PBS for 10 min at RT. Blocking and subsequent procedures were performed following the product manual. Negative control was performed by omitting one of the antibodies (only Ddx21 Ab was added). Antibodies used were listed in Table S7.

### 5.20 Imaging and Quantification

Immunofluorescence and PLA staining were imaged and visualized with either of the following microscope/software pairs: Nikon Ti2‐E widefield fluorescence microscope with NIS‐Element Viewer, Olympus FV1200 confocal microscope with FV10‐ASW, or Leica SP8 confocal microscope with LAS X. The same imaging configurations and LUTs were applied to all images in the same batch of staining. Representative images were slightly enhanced with a “brightness/contrast” layer in Photoshop. For signal quantification, nuclear masks were made using DAPI signal in ImageJ (Threshold → Watershed → ROI). Only nuclei with proper nuclear morphology (no smear or fold) were quantified to exclude damaged nuclei due to cytospin. For every nucleus in the mask, total relative fluorescence units (RFU) were measured as: [*Area* *of* *nucleus*  × (*Nuclear* *mean* *grey* *value* − *background* *mean* *grey* *value*)] using ImageJ (Measure: area, mean grey value). Total RFU of different targets were multiplied by different constants for easier visualization.

### 5.21 Plasmids

Lentiviral packaging plasmids pMD2.G (Addgene #12259), psPAX2 (#12260), Flag‐DDX21 expression plasmid (#128803), SIN3A expression lentiviral plasmid (#142686), HDAC1‐V5 expression lentiviral plasmid (#144813), HA‐KDM5A expression plasmid (#14799), eGFP‐TwinStrepTag plasmid (#141395) and BioID2‐HA construct (#74224) were purchased from Addgene. TwinStrepTag from #141395 was subcloned to the N‐terminus of SIN3A in the expression lentiviral plasmid (#142686), creating a TwinStrepTag‐SIN3A overexpression lentiviral plasmid. Full‐length HDAC2‐V5 was cloned from H1 hESC cDNA using primer pair: 5’‐ tttaatcgatATGGCGTACAGTCAAGGAGGC3’ and 5’‐ tttaacgcgtctacgtagaatcgagaccgaggagagggttagggataggcttaccGGGGTTGCTGAGCTGTTCTG‐3’, replacing HDAC1‐V5 in the HDAC1‐V5 plasmid, creating the HDAC2‐V5 expression lentiviral plasmid. BioID2‐HA construct from #74224 was subcloned into the pLVX backbone, and then the full‐length DDX21 sequence from #128803 or the SV40 NLS sequence was subcloned to the N‐terminus of BioID2‐HA to create fusion BioID2 lentiviral plasmids. Lentiviral shRNA transfer plasmids were generated by cloning shRNA sequences into pLVX‐U6‐mCherry/GFP plasmids (homemade). shRNA sequences were listed in Table S4.

### 5.22 Purification of DDX21 Protein

The full‐length human DDX21 ORF was cloned into a pET‐N‐6xHis‐TEV (Tobacco etch virus) vector. The construct was then transformed into BL21‐CodonPlus (DE3)‐RIL competent cells for expression. After expression, the cells were harvested by centrifugation and resuspended in a buffer containing Tris (pH 8.0) and 500 mM NaCl. Cell lysis was performed using a high‐pressure homogenizer, followed by affinity purification using a HisTrap HP column (Cytiva). The purified DDX21 protein was concentrated to a final concentration of 10 mg/mL and stored at ‐80°C for subsequent experiments.

### 5.23 SDS‐PAGE Western Blot

Cell lysates were prepared by resuspending cells in lysis buffer (Beyotime, P0013C) with 1x protease inhibitor (MedChemExpress) for 10 min on ice. Supernatant was extracted after 16 000 g x 10 min at 4°C centrifugation. Protein concentration was measured using Pierce BCA Protein Assay Kits (Life Technologies). Lysate was mixed with 4x Laemmli Sample Buffer (Bio‐Rad) and incubated at 70°C for 10 min. Proteins were resolved in 8%–15% SDS‐PAGE gels (Vazyme) depending on the target protein size. After electrophoresis, gels were transferred to a 0.22 µm PVDF membrane by either an overnight 4°C methanol‐based wet transfer method with 0.1% SDS or by semi‐dry transfer using the Trans‐Blot Turbo Transfer System (Bio‐Rad). The membrane was blocked in 5% BSA/PBS for 1hr and incubated with primary antibody at 4°C overnight. After 4 washes in 0.1% Tween/PBS, secondary antibody (1:5000) was applied and incubated for 1hr at RT. After 4 washes in 0.1% Tween/PBS, the membrane was immersed in Pierce ECL Western Blotting Substrate (Life Technologies) and visualized with ChemiDoc MP Imaging System (Bio‐Rad). Antibodies used were listed in Table S7.

### 5.24 Polysome Profiling

Polysome profiling of K562 cells was performed as described previously with slight modification [56]. Briefly, 5 × 10^6^ K562 cells were lysed in 350 µL lysis buffer [20 mM Tris‐HCl, pH7.5, 140 mM KCl, 5 mM MgCl_2_, 1% Triton‐X, 100 µg/mL cycloheximide, 0.5 mM DTT, 0.2U/µL RNaseOUT, 1x cOmplete EDTA‐free Protease inhibitor] for 10 mins on ice. After centrifugation to remove debris, lysate was layered on a 7%–47% linear sucrose gradient prepared by standing sucrose layers overnight. After 170 000 x g centrifugation at 4°C for 2.5 hrs, 100 µL fractions were collected at the surface of the gradient into a 96‐well plate from top to bottom. Absorbance was measured at 254 nm; fraction RNA absorbance was quantified by subtracting background absorbance.

### 5.25 Co‐Immunoprecipitation (Co‐IP)

Cell lysates were prepared by resuspending cells in Co‐IP lysis buffer (50 mM Tris pH7.4, 150 mM NaCl, 1% Triton X‐100, 1 mM EDTA) with 1x protease inhibitor for 10 min on ice. Lysate was sonicated with a Branson SLPe Digital Sonifier (5 sec on, 25 sec off, 35 amplitude, total time 120 sec). Supernatant was extracted after 16 000 g x 10 min centrifugation at 4°C. For RNase A treatment, lysates were incubated with 300 µg/mL RNase A (Sigma‐Aldrich, 10109142001) for 10 min at RT. Each IP consisted of 1–2 mg of total protein lysate. Beads were washed twice with 1 mL wash buffer (Life Technologies, 87787) before use. All incubation and washes were performed with end‐to‐end rotation. For Flag pulldown, 20 µL Flag/HA/Streptavidin magnetic beads were incubated with lysate at 4°C overnight. For V5 pulldown, 2 µg V5 antibody was incubated with lysate at 4°C overnight, followed by incubation with 25 µL Protein A/G beads for 1hr at RT. The bead complex was subsequently washed twice with 1 mL wash buffer and once with UltraPure water (Life Technologies) for 5 min each at RT. Proteins were eluted by incubating the beads at 95°C for 10 min in 1x SDS‐PAGE Sample Loading Buffer (Beyotime, P0015A). Antibodies and beads used were listed in Table S7.

### 5.26 Recombinant Protein Pull‐Down Assay

KDM5A or HDAC1 was incubated with equimolar amounts of DDX21 along with anti‐DDX21 antibody (Proteintech, 10528‐1‐AP) and Dynabeads Protein G in 500 µL of binding buffer (25 mM Tris‐HCl, 150 mM NaCl, 1 mM EDTA, 1% NP‐40 and 5% glycerol) at 4°C for 12 h with end‐to‐end rotation. Beads were washed with 1 mL wash buffer (Life Technologies, 87787) and boiled at 95°C for 10 min; the proteins were resolved by SDS‐PAGE and Western blot. Recombinant DDX21 protein was expressed and purified as described above. KDM5A (BPS Bioscience, 50110) and HDAC1 (BPS Bioscience, 50051) were purchased.

### 5.27 AlphaFold3 Protein Structure Prediction and PyMOL Visualization

AlphaFold3 structure prediction was performed on the AlphaFold Server [31]. Protein sequences used are KDM5A (P29375) and DDX21 (Q9NR30). The best‐ranked crystallography model generated by AlphaFold3 was further analysed with PyMOL (3.1.1) to visualize interacting residues.

### 5.28 Chromatin Immunoprecipitation (ChIP)

1 × 10^7^ lentivirus‐transduced K562 cells were washed twice in PBS and fixed with 5 mL 1% PFA/PBS for 10 min with swirling. Quenching was performed by adding glycine to a final concentration of 200 mM and swirling for 5 min. Cells were centrifuged, resuspended in 3 mL hypotonic buffer (10 mM Tris‐HCl pH7.5, 1.5 mM MgCl_2_, 10 mM KCl, 1 mM EDTA pH 8.0, and 0.1% IGEPAL), and incubated for 10 min at 4°C. After centrifugation (500 g x 3 min), the pellet was resuspended in 800 µL lysis buffer (20 mM Tris‐HCl pH7.5, 140 mM NaCl, 1% Triton X‐100, 0.5% SDS, 1 mM EDTA, 0.1% sodium deoxycholate, 1x protease inhibitor) on ice and incubated for 10 min. The lysate was sonicated by a Branson SLPe Digital Sonifier (2 sec on, 2 sec off, 50 amplitude, total time 90 sec). After centrifugation at 16 000 g x 10 min at 4°C, supernatant was extracted and stored at ‐80°C. To determine fragment size, 20 µL chromatin was reverse crosslinked by incubating with 76 µL TE buffer and 4 µL 5 M NaCl at 65°C overnight, then incubated with 0.5 mg/mL RNase A at 50°C for 30 min, followed by 1 mg/ml Proteinase K digestion at 55°C for 2 h. The chromatin was extracted by standard phenol/chloroform extraction and analysed on a 1.5% agarose gel. ChIP was performed when the majority of fragments were between 200–500 bp. All incubations and washes were performed with end‐to‐end rotation. Each ChIP reaction consisted of 200 µL chromatin, equivalent to 2.5 × 10^6^ cells. Chromatin was thawed and diluted 5‐fold with ChIP dilution buffer (20 mM Tris‐HCl pH7.5, 140 mM NaCl, 1% Triton X‐100, 1 mM EDTA, 0.1% sodium deoxycholate, 1x protease inhibitor) to lower SDS concentration to 0.1%. To pre‐clear the chromatin, 1 µL Flag bead was added to every 100 µL diluted chromatin and incubated for 2 h at 4°C. Antibody (1 µg DDX21, 1 µg KDM5A, 0.25 µg H3K4me3, or 1 µg IgG) was incubated with 15 µL Protein G Dynabeads in 100 µL ChIP dilution buffer for 1 h. The antibody‐bead complex was washed twice with 1 mL ChIP dilution buffer and subsequently incubated with the pre‐cleared chromatin at 4°C overnight. Chromatin was sequentially washed with Wash Buffer 1 (20 mM Tris‐HCl pH8.0, 150 mM NaCl, 1% Triton X‐100, 0.1%SDS, 2 mM EDTA), Wash Buffer 2 (20 mM Tris‐HCl pH8.0, 500 mM NaCl, 1% Triton X‐100, 0.1%SDS, 2 mM EDTA), Wash Buffer 3 (10 mM Tris‐HCl, pH8.0, 250 mM LiCl, 1% IGEPAL, 1% sodium deoxycholate, 1 mM EDTA) and lastly twice with TE buffer. Each wash was performed with 1 mL buffer for 5 min at 4°C. Chromatin was eluted with 400 µL TES elution buffer (10 mM Tris‐HCl pH7.5, 1 mM EDTA, 1%SDS) at 65°C for 30 min. For input control, TES elution buffer was added to top up input samples to 400 µL. Eluates and inputs were reverse crosslinked in 200 mM NaCl at 65°C overnight, then incubated with 0.5 mg/ml RNase A at 50°C for 30 min, followed by 1 mg/ml Proteinase K at 55°C for 2hr. Standard phenol/chloroform extraction was performed with the addition of 0.1x volume of 3 M sodium acetate (pH 3.0) and 1 µL nucleic acid isolation enhancer (Abcam) to the extracted aqueous layer. DNA pellet was resuspended in 100 µL water, and 4 µL was used for a 10 µL qPCR reaction. Primers used are listed in Table S8.

### 5.29 Sequential Chromatin Immunoprecipitation (ChIP‐reChIP)

Cell fixation, lysis, and sonication were performed as above. For the first ChIP, each reaction consisted of 4 mL diluted chromatin (1 × 10^7^ cells equivalent), 60 µL Protein G Dynabead, and 4 µg of Flag/IgG antibody. Chromatin washing was performed as above. For elution of the first ChIP, 100 µL reCHIP elution buffer (10 mM Tris‐HCl, pH7.5, 1 mM EDTA, pH8.0, 1%SDS, 10 mM DTT) was added and incubated at 37°C for 30 min. The elution was repeated once to yield a total of 200 µL eluate. The eluate is diluted 5‐fold with reCHIP dilution buffer (20 mM Tris‐HCl, pH7.5, 175 mM NaCl, 1% Triton X‐100, 1 mM EDTA pH8.0, 0.1% sodium deoxycholate, 1x protease inhibitor). 40 µL of the diluted eluate was stored for qPCR analysis, while the remaining eluate was used for the second ChIP by containing 1.5 µg antibody and 15 µL Protein G Dynabead. Bead‐Ab complex preparation, incubation, and washes were carried out as above. Second ChIP was eluted with 100 µL TES elution buffer (10 mM Tris‐HCl pH7.5, 1 mM EDTA, 1%SDS) at 65°C for 30 min twice. Eluates and inputs were extracted as above.

### 5.30 RNA Extraction and Quantitative Reverse Transcription PCR (qPCR)

Total RNA was extracted by FastPure Cell/Tissue Total RNA Isolation Kit V2 (Vazyme) or Direct‐zol RNA Microprep kit (Zymo Research) with gDNA removal. Reverse transcription and qPCR were performed using PrimeScript RT Master Mix (Takara) and TB Green Premix Ex Taq II (Takara), respectively. cDNA was diluted 20 fold, and 2 µL was used for each 10 µL qPCR reaction. Each target was performed in triplicate on QuantStudio 7 Flex (Applied Biosystems) in “fast” mode, with other settings at default. Cycle threshold values were converted to fold‐change expression by 2^ ^−ddCT^ using Gapdh as control. qPCR primer sequences were listed in Table S8.

### 5.31 TapeStation rRNA Profiling

rRNA quantification and profiling were performed using RNA ScreenTape Analysis or High Sensitivity RNA ScreenTape (Agilent). 18S, 28S rRNA, and total RNA concentrations were determined accordingly after automatic peak assignment. Samples intended for comparison were processed with equal cell numbers for RNA extraction, performed concurrently using the identical extraction method and kit.

### 5.32 DDX21‐BioID2 Proximity Protein Pulldown and Mass Spectrometry

BioID2 detection of the protein interactome was performed as previously described with some modifications [57]. K562 cells were transduced with lentivirus carrying DDX21‐BioID2‐HA or SV40NLS‐BioID2‐HA. Nuclear localization of the fusion BioID2 protein was verified by HA immunofluorescence staining. After 5 days of transduction, cells were incubated in 50 µM D‐biotin (Sigma) for 16 h under culture conditions in triplicate. 30 million cells were lysed in 1 mL fresh lysis buffer [50 mM NaF, 100 mM NaCl, 5 mM EDTA, 50 mM HEPES, 0.2%SDS, 0.5% IGEPAL, 1 mM PMSF, 1.5 mM Na_3_VO_4_, 1 mM DTT, 1x protease inhibitor cocktail (Roche) and 1 µL of 30U/µL benzonase (Sigma E1014) for 10 min on ice. Lysate was pulse‐sonicated at 50% Amplitude (1 sec on, 1 sec off) for 5 min using a Branson SLPe (2.4 mm tip diameter). After centrifugation at 16000 g for 10 min at 4 °C, the supernatant was diluted to 0.1% SDS. Protein concentration was determined by the BCA method. 15 mg protein lysates were incubated with 1 mL Streptavidin beads (Beyotime) at 4°C overnight with end‐to‐end rotation. Beads were washed with Wash Buffer 1 twice and Wash Buffers 2–4 once, each for 5 min with end‐to‐end rotation (Wash Buffer 1: 2% SDS; Wash Buffer 2: 0.1% sodium deoxycholate, 1% Triton X‐100, 500 mM NaCl, 1 mM EDTA, 50 mM HEPES pH 7.5. Wash Buffer 3: 10 mM Tris‐HCl pH 8.0, 250 mM LiCl, 1 mM EDTA, 0.5% IGEPAL, 0.5% sodium deoxycholate. Wash Buffer 4: 50 mM Tris‐HCl, pH8.0). The bead‐protein complex was frozen and sent for 4D‐Data Independent Acquisition (DIA) label‐free quantitative LC‐MS/MS analysis (Omicsolution). Beads were washed in 1 mL 50 mM NH_4_HCO_3_. After centrifugation, beads were reduced with 2 µL of 0.5 M Tris 2‐carboxylethyl phosphine (TCEP) at 37°C for 60 min and alkylated with 4 µL of 1 M iodoacetamide (IAM) for 40 min at RT. Five‐fold volume of cold acetone was added to precipitate the proteins at −20°C overnight. After centrifugation at 12 000 g x 20 min at 4°C, the pellet was washed twice with 1 mL of pre‐chilled 90% acetone aqueous solution and resuspended with 100 µL of 100 mM triethylammonium bicarbonate (TEAB) buffer. Trypsin was added at a 1:50 trypsin‐to‐protein mass ratio and incubated at 37°C overnight, followed by desalting with Pierce C18 Spin Tips, and then dried in a speed vacuum concentrator.

The UltiMate 3000 (ThermoFisher Scientific) liquid chromatography system was connected to the timsTOF Pro 2, an ion‐mobility spectrometry quadrupole time‐of‐flight mass spectrometer (Bruker Daltonics). Samples were reconstituted in 0.1% FA and 200 ng peptide was separated by AUR3‐15075C18 column (15 cm length, 75 µm i.d, 1.7 µm particle size, 120 Å pore size, IonOpticks) with a 30 min gradient starting at 2.2% Buffer B (80% ACN with 0.1% FA) followed by a stepwise increase to 28% in 16 min, 38% in 8 min, 90% in 3 min and stayed there for 3 min. The column flow rate was maintained at 400nL/min with the column temperature of 50°C. DIA data were acquired in the diaPASEF mode. We defined 22 × 40 Th precursor isolation windows from m/z 349 to 1229. To adapt the MS1 cycle time, we set the repetitions to variable steps (2–5) in the 13‐scan diaPASEF scheme in our experiment. During PASEF MSMS scanning, the collision energy was ramped linearly as a function of the mobility from 59 eV at 1/K0 = 1.6 Vs/cm^2^ to 20 eV at 1/K0 = 0.6 Vs/cm^2^.

Proteomic spectral analysis and quantification were performed by the MS service provider using Spectronaut 19 (Biognosys) with default settings. The database used was Homo Sapiens (Ver. 2024, 20608 entries) from UniProt. Trypsin was the digestion enzyme, and specific was the digest type. Carbamidomethylation on cysteine was specified as the fixed modification. Oxidation on methionine, Acetyl on protein N‐term were specified as the variable modifications. Retention time prediction type was set to dynamic iRT. Data extraction was determined by Spectronaut based on the extensive mass calibration. Spectronaut will determine the ideal extraction window dynamically depending on iRT calibration and gradient stability. Q‐value (FDR) cutoff at the precursor, peptide, and protein level was 1%. Decoy generation was set to mutated, which is similar to scrambled but will only apply a random number of AA position swaps (min = 2, max = length/2). Normalization strategy was set to Local normalization. Peptides that passed the 1% Q‐value cutoff were used to calculate the major group quantities with the MaxLFQ method. To obtain the DDX21‐specific affinity protein list, Spectronaut output was further analysed using MSstats. By comparing proteins obtained from DDX21‐BioID2 to those of NLS‐BioID2, significant proteins (*q* < 0.05) were considered as DDX21 affinity proteins.

### 5.33 RNA‐Seq and Data Analysis

Biological triplicates (embryos or culture cells) were collected for each transcriptomic dataset. Total RNA was extracted using the Direct‐zol RNA Microprep kit (Zymo Research) with gDNA removal. Enrichment of mRNA, construction of cDNA library (ABclonal RK20350), and PE150 sequencing (NovaSeq 6000) were performed by HaploX. Reads were aligned to hg38 or mm10 using STAR aligner (2.7.11b) [–outFilterMultimapNmax 20 –alignSJoverhangMin 8 –alignSJDBoverhangMin 1 –outFilterMismatchNoverReadLmax 0.04 –alignIntronMin 20 –alignIntronMax 1 000 000 –alignMatesGapMax 1 000 000 –outFilterType BySJout –sjdbScore 1]. The count matrix was constructed by paired‐end counting of exons using subread:featureCounts (2.0.8). Batch normalization for batch1 (WT‐1, WT‐2, KO‐1, KO‐2, KO‐3) and batch2 (WT‐4, WT‐5, WT‐6, KO‐4, KO‐5) was performed by Combat‐seq (3.36.1). To remove low‐expressing genes, genes where less than half of the samples had EdgeR:CPM >1 were filtered out. Normalized counts were obtained by DEseq2:RLE (1.44.0). Gene name/ID conversion was performed using AnnotationDbi (1.66.0). Genes with Log_2_FC > 1 and *p.adj* < 0.05 were used for subsequent differential analysis. GO/KEGG enrichment and GSEA were performed using clusterProfiler (4.12.6) and pathview (1.40.0). GO term clustering and visualization were performed using Cytoscape (3.10.3). Volcano plot and heatmap were generated using EnhancedVolcano (1.22.0) and ComplexHeatmap (2.20.0), respectively. Raw and processed data files were deposited in the Gene Expression Omnibus (GEO) under accession number GSE296783.

### 5.34 CUT&Tag and Data Analysis

CUT&Tag libraries were constructed in biological duplicates using the CUTANA CUT&Tag Kit (Epicypher) according to the product manual. 3–6 × 10^4^ FACS‐sorted live fetal liver LK cells were used per library with 15–18 cycles of PCR amplification. Libraries were validated by the Agilent high‐sensitivity DNA kit. PE150 sequencing (NovaSeq X) was performed by HaploX. QC was performed with FastQC (0.12.1). Adapter and 3' polyG were removed with fastp (0.23.4) ‐g –detect_adapter_for_pe. Reads were aligned to UCSC mm10 genome using bowtie2 (2.5.4) (–local –very‐sensitive –no‐mixed –no‐discordant –phred33 –threads 8 ‐I 20 ‐X 1000). Alignments with MAPQ <30 (including unmapped and multi‐mapped regions) and unwanted regions/chromosomes (chrM, random, chrUn, chrEBV) were filtered out using samtools (1.20.0). For H3K27Ac, duplicate reads were removed with Picard (3.2.0). BAM files were converted to BEDPE using bedtools (2.31.1). Broadpeaks were called using MACS2 (2.2.9.1) with an FDR cutoff of *q* < 0.1 to 0.01, depending on the S/N ratio. Peaks from replicates were merged when there was 1 bp overlap using bedtools. Normalized and scaled per‐million bigWig tracks were generated using deepTools:bamCoverage (3.5.5) with scaling factor calculated as 1 000 000/(EdgeR: TMM scale factor on reads in 10 000 bp background bin * library size). Blacklist regions of mm10 obtained from ENCODE were filtered out. BigWig files from replicates were merged by first averaging the replicates when converting to a merged bedGraph using wiggleTools:write_bg (1.2.11), then converting back to bigWig using uscs:bedGraphToBigWig (469). Signal tracks were visualized in Interactive Genome Viewer (IGV) (2.16.1) with a consistent data range across all CUT&Tag and ATAC tracks to enable direct comparison. For analysis of rDNA, cleaned reads after fastp removal of adaptors and polyG were mapped to the mouse rDNA sequence (NCBI BK000964.3) with Bowtie2 using the same parameters. Alignments with MAPQ <30 were filtered out using samtools. Merged rDNA bigWig tracks from replicates were generated in the same manner as above. rDNA signals were visualized in IGV with published curated annotations studying hematopoietic TFs [58]. Raw and processed data files were deposited in the GEO under accession number GSE296889.

Peaks and normalized bigWig tracks were used for downstream analysis. Profile and heatmap plots in the gene body and around the TSS were generated using deepTools. Distance to the Ddx21 peak was generated by deepTools using the midpoint of the Ddx21 peak as “TSS”. Pearson correlation of bigWig among targets was generated using deepTools:plotCorrelation. To obtain co‐binding peaks among H3K4me3, Ddx21, and Kdm5a, peak intersection was performed using bedtools: intersect, where peaks were intersected if there was>1 bp overlap. Peak distribution in the genome was analyzed using ChIPseeker (1.40.0).

To perform Pearson correlation between promoter bindings and their respective RNA expression, CUT&Tag signal counts in promoters (±1500 bp of TSS) were obtained using featureCounts and multiplied by the respective scaling factor as stated above. Normalized RNA counts were mapped to promoters by matching genes to their respective promoters. All counts were transformed to Log_2_(Counts+1) and subjected to Pearson correlation using ggpubr (0.6.0).

For analysis of promoter states, promoters were defined as ±1500 bp around the TSS. A combination of the presence of histone binding peaks within the promoter and presence of RNA expression (CPM>1) were used to define promoter states: active (promoter H3K4me3+, genebody H3K27me3‐, RNA+), poised (promoter H3K4me3+, genebody H3K27me3‐, RNA‐), bivalent (promoter H3K4me3+, promoter H3K27me3+) and repressed (Promoter H3K4me3‐, genebody H3K27me3+). For analysis of enhancers, enhancers were defined as ± 3000 bp regions around H3K4me1 peaks located outside promoters. Combination of presence of histone and ATAC peaks were used to define enhancer states [59]: poised enhancer (H3K27me+, H3K4me1+), primed enhancer (H3K27me3‐, H3K4me1+, H3K27Ac^low^, ATAC^low^) and active enhancer (H3K27me3‐, H3K4me1+, H3K27Ac^mid/high^, ATAC^mid/high^).

Differential binding analysis was performed using DiffBind (3.18.0). A consensus peak list was created by only considering peaks that appear in over half of the samples. Counts in consensus peaks were obtained with summits = 1200. Differential peaks were obtained with TMM normalization. Genes with promoters (±1500 bp upstream of the TSS) having differential peak binding (FC > 0.3, *p.adj* < 0.05) were used for subsequent GO enrichment analysis. Gene expressions in these promoters are considered up (Log2FC > 0.5, *p.adj* < 0.05) or down (Log2FC < 0.5, *p.adj* < 0.05). The promoter differential peak binding results were also used to extract Log2FC values of H3K4me3 binding.

### 5.35 ATAC‐Seq and Data Analysis

ATAC‐seq libraries were constructed in biological duplicates using Hyperactive ATAC‐Seq Library Prep Kit for Illumina (Vazyme #TD711) and TruePrep Index Kit V2 for Illumina (Vazyme #TD202) according to the product manual. 6.5 × 10^4^ cells were used per library with 13 cycles of PCR amplification. Single size exclusion was performed to remove small fragments using beads provided in the kit. Libraries were validated using the Agilent High Sensitivity DNA kit. PE150 sequencing (NovaSeq X) was performed by HaploX. Downstream processing of ATAC‐seq was similar to CUT&Tag with slight differences. Duplicates were removed with Picard, and peaks were extended with (–nomodel –shift 100 –ext 200) to account for Tn5 targeting during MACS2 peak calling. Raw and processed data files were deposited in GEO under accession number GSE296888.

### 5.36 Statistical Analysis

For all immunofluorescence, flow cytometry, and biochemical analyses, a two‐tailed unpaired Student's t‐test was used when comparing between 2 groups, and one‐way ANOVA was used for multi‐group comparisons. Brown‐Forsythe test was performed to determine the homogeneity of group variance. Ordinary ANOVA with Tukey post‐hoc testing was used on data with equal variance, while Welch ANOVA with Dunnett T3 post‐hoc testing was used on data with unequal variance. Graphs were plotted using GraphPad Prism 10.

## Author Contributions

A.O.L. and H.H.C. conceived the project and designed the research studies. A.O.L. and C.L. performed the experiments and analyzed the data. X.H., Y.C., J.L., H.L., X.C., F.Z., and R.N. assisted in the preparation of reagents and protein interaction studies. Z.F. generated the recombinant proteins. K.T.L. and B.H. provided assistance with project design and methodology. A.O.L. and H.H.C. drafted the manuscript. All authors approved the manuscript for submission.

## Conflicts of Interest

The authors declare no conflicts of interest.

## Supporting information




**Supporting File 1**: advs78524‐sup‐0001‐table1.pdf.

**Supporting File 2**: advs78524‐sup‐0002‐figures 3.pdf.

## Data Availability

The data that support the findings of this study are openly available in NCBI BioProject at https://www.ncbi.nlm.nih.gov/bioproject/?term = PRJNA1258612, reference number PRJNA1258612.

## References

[1] D.‐M. Popescu , R. A. Botting , E. Stephenson , et al., “Decoding Human Fetal Liver Haematopoiesis,” Nature 574, no. 7778 (2019): 365–371, 10.1038/s41586-019-1652-y. PMC6861135 31597962

[2] I. L. Weissman and J. A. Shizuru , “The Origins of the Identification and Isolation of Hematopoietic Stem Cells, and Their Capability to Induce Donor‐specific Transplantation Tolerance and Treat Autoimmune Diseases,” Blood 112, no. 9 (2008): 3543–3553, 10.1182/blood-2008-08-078220. PMC2574516 18948588

[3] C. Ni and M. Buszczak , “Ribosome Biogenesis and Function in Development and Disease,” Development 150, no. 5 (2023), 10.1242/dev.201187. PMC10108708 36897354

[4] L. Da Costa , T. Leblanc , and N. Mohandas , “Diamond‐Blackfan Anemia,” Blood 136, no. 11 (2020): 1262–1273. 10.1182/blood.2019000947 PMC7483438 32702755

[5] J. C. Ulirsch , J. M. Verboon , S. Kazerounian , et al., “The Genetic Landscape of Diamond‐Blackfan Anemia,” The American Journal of Human Genetics 103, no. 6 (2018): 930–947, 10.1016/j.ajhg.2018.10.027. PMC6288280 30503522

[6] D. Iskander , A. Karadimitris , and I. Roberts , “Harnessing Single‐Cell Technologies in the Search for New Therapies for Diamond–Blackfan Anemia Syndrome,” Experimental Hematology 135 (2024): 104235, 10.1016/j.exphem.2024.104235. 38740323

[7] K. Dörner , C. Ruggeri , I. Zemp , and U. Kutay , “Ribosome biogenesis factors—From names to functions,” The EMBO Journal 42, no. 7 (2023): 112699, 10.15252/embj.2022112699. PMC10068337 36762427

[8] J. A. Magee and R. A. J. Signer , “Developmental Stage‐Specific Changes in Protein Synthesis Differentially Sensitize Hematopoietic Stem Cells and Erythroid Progenitors to Impaired Ribosome Biogenesis,” Stem Cell Reports 16, no. 1 (2021): 20–28, 10.1016/j.stemcr.2020.11.017. PMC7815942 33440178

[9] J. A. Saba , K. Liakath‐Ali , R. Green , and F. M. Watt , “Translational Control of Stem Cell Function,” Nature Reviews Molecular Cell Biology 22, no. 10 (2021): 671–690, 10.1038/s41580-021-00386-2. 34272502

[10] K. Lv , C. Gong , C. Antony , et al., “HectD1 controls Hematopoietic Stem Cell Regeneration by Coordinating Ribosome Assembly and Protein Synthesis,” Cell Stem Cell 28, no. 7 (2021): 1275–1290.e9, 10.1016/j.stem.2021.02.008. PMC8254759 33711283

[11] Z. Zheng , S. Yang , F. Gou , et al., “The ATF4‐RPS19BP1 Axis Modulates Ribosome Biogenesis to Promote Erythropoiesis,” Blood 144, no. 7 (2024): 742–756, 10.1182/blood.2023021901. 38657191

[12] T. M. Chlon , E. Stepanchick , C. E. Hershberger , et al., “Germline DDX41 Mutations Cause Ineffective Hematopoiesis and Myelodysplasia,” Cell Stem Cell 28, no. 11 (2021): 1966–1981, 10.1016/j.stem.2021.08.004. PMC8571055 34473945

[13] H. Bi , K. Ren , P. Wang , et al., “DDX41 resolves G‐quadruplexes to Maintain Erythroid Genome Integrity and Prevent cGAS‐mediated Cell Death,” Nature Communications 16, no. 1 (2025): 7195, 10.1038/s41467-025-62307-7. PMC12325982 40764304

[14] E. Calo , R. A. Flynn , L. Martin , R. C. Spitale , H. Y. Chang , and J. Wysocka , “RNA Helicase DDX21 Coordinates Transcription and Ribosomal RNA Processing,” Nature 518, no. 7538 (2015): 249–253, 10.1038/nature13923. PMC4827702 25470060

[15] D. Henning , R. B. So , R. Jin , L. F. Lau , and B. C. Valdez , “Silencing of RNA Helicase II/Guα Inhibits Mammalian Ribosomal RNA Production,” Journal of Biological Chemistry 278, no. 52 (2003): 52307–52314, 10.1074/jbc.M310846200. 14559904

[16] K. E. Sloan , M. S. Leisegang , C. Doebele , et al., “The Association of Late‐acting snoRNPs With human Pre‐ribosomal Complexes Requires the RNA Helicase DDX21,” Nucleic Acids Research 43, no. 1 (2015): 553–564, 10.1093/nar/gku1291. PMC4288182 25477391

[17] P. Bora , L. Gahurova , A. Hauserova , et al., “DDX21 is a p38‐MAPK‐sensitive Nucleolar Protein Necessary for Mouse Preimplantation Embryo Development and Cell‐fate Specification,” Open Biology 11, no. 7 (2021): 210092, 10.1098/rsob.210092. PMC8277471 34255976

[18] E. Calo , B. Gu , M. E. Bowen , et al., “Tissue‐selective Effects of Nucleolar Stress and rDNA Damage in Developmental Disorders,” Nature 554, no. 7690 (2018): 112–117, 10.1038/nature25449. PMC5927778 29364875

[19] K. Koltowska , K. S. Okuda , M. Gloger , et al., “The RNA Helicase Ddx21 Controls Vegfc‐driven Developmental Lymphangiogenesis by Balancing Endothelial Cell Ribosome Biogenesis and p53 Function,” Nature Cell Biology 23, no. 11 (2021): 1136–1147, 10.1038/s41556-021-00784-w. 34750583

[20] W. Miao , D. F. Porter , V. Lopez‐Pajares , et al., “Glucose Dissociates DDX21 Dimers to Regulate mRNA Splicing and Tissue Differentiation,” Cell 186, no. 1 (2023): 80–97, 10.1016/j.cell.2022.12.004. PMC10171372 36608661

[21] Y. Xiao , J. Pan , J. Fan , et al., “RNA Helicase DDX21 Sustains Hematopoietic Stem Cell Homeostasis by Preventing Nucleolar Stress,” Cell Investigation 2, no. 1 (2026): 100060, 10.1016/j.clnves.2025.100060.

[22] M. J. Kiel , Ö. H. Yilmaz , T. Iwashita , O. H. Yilmaz , C. Terhorst , and S. J. Morrison , “SLAM family Receptors Distinguish Hematopoietic Stem and Progenitor Cells and Reveal Endothelial Niches for Stem Cells,” Cell 121, no. 7 (2005): 1109–1121, 10.1016/j.cell.2005.05.026. 15989959

[23] K. Chen , J. Liu , S. Heck , J. A. Chasis , X. An , and N. Mohandas , “Resolving the Distinct Stages in Erythroid Differentiation Based on Dynamic Changes in Membrane Protein Expression During Erythropoiesis,” Proceedings of the National Academy of Sciences 106, no. 41 (2009): 17413–17418, 10.1073/pnas.0909296106. PMC2762680 19805084

[24] V. Rebel , C. Miller , C. Eaves , and P. Lansdorp , “The Repopulation Potential of Fetal Liver Hematopoietic Stem Cells in Mice Exceeds That of Their Liver Adult Bone Marrow Counterparts,” Blood 87, no. 8 (1996): 3500–3507, 10.1182/blood.V87.8.3500.bloodjournal8783500. 8605370

[25] E. K. S. McRae , S. J. Dupas , E. P. Booy , R. S. Piragasam , R. P. Fahlman , and S. A. McKenna , “An RNA Guanine Quadruplex Regulated Pathway to TRAIL‐sensitization by DDX21,” Rna 26, no. 1 (2020): 44–57, 10.1261/rna.072199.119. PMC6913123 31653714

[26] W. Assaily , D. A. Rubinger , K. Wheaton , et al., “ROS‐mediated p53 Induction of Lpin1 Regulates Fatty Acid Oxidation in Response to Nutritional Stress,” Molecular Cell 44, no. 3 (2011): 491–501, 10.1016/j.molcel.2011.08.038. 22055193

[27] Y.‐H. Xing , R.‐W. Yao , Y. Zhang , et al., “SLERT Regulates DDX21 Rings Associated With Pol I Transcription,” Cell 169, no. 4 (2017): 664–678, 10.1016/j.cell.2017.04.011. 28475895

[28] J. Badhai , A.‐S. Fröjmark , E. J. Davey , J. Schuster , and N. Dahl , “Ribosomal protein S19 and S24 insufficiency cause distinct cell cycle defects in Diamond–Blackfan anemia,” Biochimica et Biophysica Acta (BBA)—Molecular Basis of Disease 1792, no. 10 (2009): 1036–1042, 10.1016/j.bbadis.2009.08.002. PMC2759502 19689926

[29] V. Munugalavadla , L. C. Dore , B. L. Tan , et al., “Repression of c‐kit and Its Downstream Substrates by GATA‐1 Inhibits Cell Proliferation During Erythroid Maturation,” Molecular and Cellular Biology 25, no. 15 (2005): 6747–6759, 10.1128/MCB.25.15.6747-6759.2005. PMC1190349 16024808

[30] F. Lessard , S. Igelmann , C. Trahan , et al., “Senescence‐associated Ribosome Biogenesis Defects Contributes to Cell Cycle Arrest Through the Rb Pathway,” Nature Cell Biology 20, no. 7 (2018): 789–799, 10.1038/s41556-018-0127-y. 29941930

[31] J. Abramson , J. Adler , J. Dunger , et al., “Accurate Structure Prediction of Biomolecular Interactions With AlphaFold 3,” Nature 630, no. 8016 (2024): 493–500, 10.1038/s41586-024-07487-w. PMC11168924 38718835

[32] G. G. Wang , J. Song , Z. Wang , et al., “Haematopoietic Malignancies Caused by Dysregulation of a Chromatin‐binding PHD Finger,” Nature 459, no. 7248 (2009): 847–851, 10.1038/nature08036. PMC2697266 19430464

[33] T. H. Holmström , A. Mialon , M. Kallio , et al., “c‐Jun Supports Ribosomal RNA Processing and Nucleolar Localization of RNA Helicase DDX21,” Journal of Biological Chemistry 283, no. 11 (2008): 7046–7053, 10.1074/jbc.M709613200. 18180292

[34] R. Liefke , F. Oswald , C. Alvarado , et al., “Histone demethylase KDM5A is an integral part of the core Notch–RBP‐J repressor complex,” Genes & Development 24, no. 6 (2010): 590–601, 10.1101/gad.563210. PMC2841336 20231316

[35] I. O. Torres , K. M. Kuchenbecker , C. I. Nnadi , R. J. Fletterick , M. J. S. Kelly , and D. G. Fujimori , “Histone Demethylase KDM5A Is Regulated by Its Reader Domain Through a Positive‐feedback Mechanism,” Nature Communications 6, no. 1 (2015): 6204, 10.1038/ncomms7204. PMC5080983 25686748

[36] L. Wu , J. Cao , W. L. Cai , et al., “KDM5 histone Demethylases Repress Immune Response via Suppression of STING,” PLoS Biology 16, no. 8 (2018): 2006134, 10.1371/journal.pbio.2006134. PMC6095604 30080846

[37] N. Lopez‐Bigas , T. A. Kisiel , D. C. DeWaal , et al., “Genome‐wide Analysis of the H3K4 Histone Demethylase RBP2 Reveals a Transcriptional Program Controlling Differentiation,” Molecular Cell 31, no. 4 (2008): 520–530, 10.1016/j.molcel.2008.08.004. PMC3003864 18722178

[38] A.‐S. B. Brier , A. Loft , J. G. S. Madsen , et al., “The KDM5 family Is Required for Activation of Pro‐proliferative Cell Cycle Genes During Adipocyte Differentiation,” Nucleic Acids Research 45, no. 4 (2017): 1743–1759, 10.1093/nar/gkw1156. PMC5389521 27899593

[39] E. V. Benevolenskaya , H. L. Murray , P. Branton , R. A. Young , and W. G. Kaelin , “Binding of pRB to the PHD Protein RBP2 Promotes Cellular Differentiation,” Molecular Cell 18, no. 6 (2005): 623–635, 10.1016/j.molcel.2005.05.012. 15949438

[40] M. Wu , G. Xu , C. Han , et al., “lncRNA SLERT Controls Phase Separation of FC/DFCs to Facilitate Pol I Transcription,” Science 373, no. 6554 (2021): 547–555, 10.1126/science.abf6582. 34326237

[41] Y. Tanaka , K. Okamoto , K. Teye , et al., “JmjC Enzyme KDM2A Is a Regulator of rRNA Transcription in Response to Starvation,” The EMBO Journal 29, no. 9 (2010): 1510–1522, 10.1038/emboj.2010.56. PMC2876952 20379134

[42] D. Frescas , D. Guardavaccaro , F. Bassermann , R. Koyama‐Nasu , and M. Pagano , “JHDM1B/FBXL10 is a Nucleolar Protein That Represses Transcription of Ribosomal RNA Genes,” Nature 450, no. 7167 (2007): 309–313, 10.1038/nature06255. 17994099

[43] S. Kadauke , M. I. Udugama , J. M. Pawlicki , et al., “Tissue‐specific Mitotic Bookmarking by Hematopoietic Transcription Factor GATA1,” Cell 150, no. 4 (2012): 725–737, 10.1016/j.cell.2012.06.038. PMC3425057 22901805

[44] Y. Tang , T. Ling , R. Mehmood , et al., “RPS19 and RPL5 Haploinsufficient Models Reveal Divergent Ribosomal Subunit Controls of Fetal Hematopoiesis,” Nature Communications 17, no. 1 (2026): 4984, 10.1038/s41467-026-71727-y. PMC13237010 41951665

[45] D. Iskander , G. Wang , E. F. Heuston , et al., “Single‐cell Profiling of human Bone Marrow Progenitors Reveals Mechanisms of Failing Erythropoiesis in Diamond‐Blackfan Anemia,” Science Translational Medicine 13, no. 610 (2021): abf0113, 10.1126/scitranslmed.abf0113. 34516827

[46] E. M. Payne , N. Bolli , J. Rhodes , et al., “Ddx18 is Essential for Cell‐cycle Progression in Zebrafish Hematopoietic Cells and is Mutated in human AML,” Blood 118, no. 4 (2011): 903–915, 10.1182/blood-2010-11-318022. PMC3148170 21653321

[47] Y. Zhang , G. W. Wolf , K. Bhat , et al., “Ribosomal Protein L11 Negatively Regulates Oncoprotein MDM2 and Mediates a p53‐dependent Ribosomal‐stress Checkpoint Pathway,” Molecular and Cellular Biology 23, no. 23 (2003): 8902–8912, 10.1128/MCB.23.23.8902-8912.2003. PMC262682 14612427

[48] S. Hubackova , E. Davidova , S. Boukalova , et al., “Replication and Ribosomal Stress Induced by Targeting Pyrimidine Synthesis and Cellular Checkpoints Suppress p53‐deficient Tumors,” Cell Death & Disease 11, no. 2 (2020): 110, 10.1038/s41419-020-2224-7. PMC7007433 32034120

[49] Y. Boglev , A. P. Badrock , A. J. Trotter , et al., “Autophagy Induction Is a Tor‐ and Tp53‐independent Cell Survival Response in a Zebrafish Model of Disrupted Ribosome Biogenesis,” PLoS Genetics 9, no. 2 (2013): 1003279, 10.1371/journal.pgen.1003279. PMC3567153 23408911

[50] G. V. Yadav , A. Chakraborty , T. Uechi , and N. Kenmochi , “Ribosomal Protein Deficiency Causes Tp53‐independent Erythropoiesis Failure in Zebrafish,” The International Journal of Biochemistry & Cell Biology 49 (2014): 1–7, 10.1016/j.biocel.2014.01.006. 24417973

[51] Z. Macecková , A. Kubícková , J. De Sanctis , and M. Hajdúch , “Effect of Glucocorticosteroids in Diamond‐Blackfan Anaemia: Maybe Not as Elusive as It Seems,” International Journal of Molecular Sciences 23, no. 3 (2022): 1886, 10.3390/ijms23031886. PMC8837118 35163808

[52] A. Vlachos and E. Muir , “How I Treat Diamond‐Blackfan Anemia,” Blood 116, no. 19 (2010): 3715–3723, 10.1182/blood-2010-02-251090. PMC2981532 20651069

[53] G. Tiscornia , O. Singer , and I. M. Verma , “Production and Purification of Lentiviral Vectors,” Nature Protocols 1, no. 1 (2006): 241–245, 10.1038/nprot.2006.37. 17406239

[54] Y. Hu and G. K. Smyth , “ELDA: Extreme Limiting Dilution Analysis for Comparing Depleted and Enriched Populations in Stem Cell and Other Assays,” Journal of Immunological Methods 347, no. 1‐2 (2009): 70–78, 10.1016/j.jim.2009.06.008. 19567251

[55] M. Beltran , C. M. Yates , L. Skalska , et al., “The Interaction of PRC2 With RNA or Chromatin Is Mutually Antagonistic,” Genome Research 26, no. 7 (2016): 896–907, 10.1101/gr.197632.115. PMC4937559 27197219

[56] M. Sobhany and R. E. Stanley , “Polysome Profiling Without Gradient Makers or Fractionation Systems,” Journal of Visualized Experiments 1, no. 172 (2021): e62680, 10.3791/62680. PMC8296745 34152326

[57] K. J. Roux , D. I. Kim , B. Burke , and D. G. May , “BioID: A Screen for Protein‐Protein Interactions,” Current Protocols in Protein Science 91, no. 1 (2018): 15, 10.1002/cpps.51. PMC6028010 29516480

[58] C. Antony , S. S. George , J. Blum , et al., “Control of Ribosomal RNA Synthesis by Hematopoietic Transcription Factors,” Molecular Cell 82, no. 20 (2022): 3826–3839, 10.1016/j.molcel.2022.08.027. PMC9588704 36113481

[59] A. Barral and J. Dejardin , “The Chromatin Signatures of Enhancers and Their Dynamic Regulation,” Nucleus 14, no. 1 (2023): 2160551, 10.1080/19491034.2022.2160551. PMC9828845 36602897

