# Supplementary material for: Non‐Canonical Function of DDX21 in Fetal Hematopoiesis Antagonizes KDM5A‐Mediated Repression of Ribosomal and Hematopoietic Genes
Source: Adv Sci (Weinh). 2026 Nov 9:e78524. Online ahead of print. doi: 10.1002/advs.78524 (PMC13748770; doi:10.1002/advs.78524)
Supplement: Supplementary file 1 [file ADVS-9999-e78524-s001.pdf]

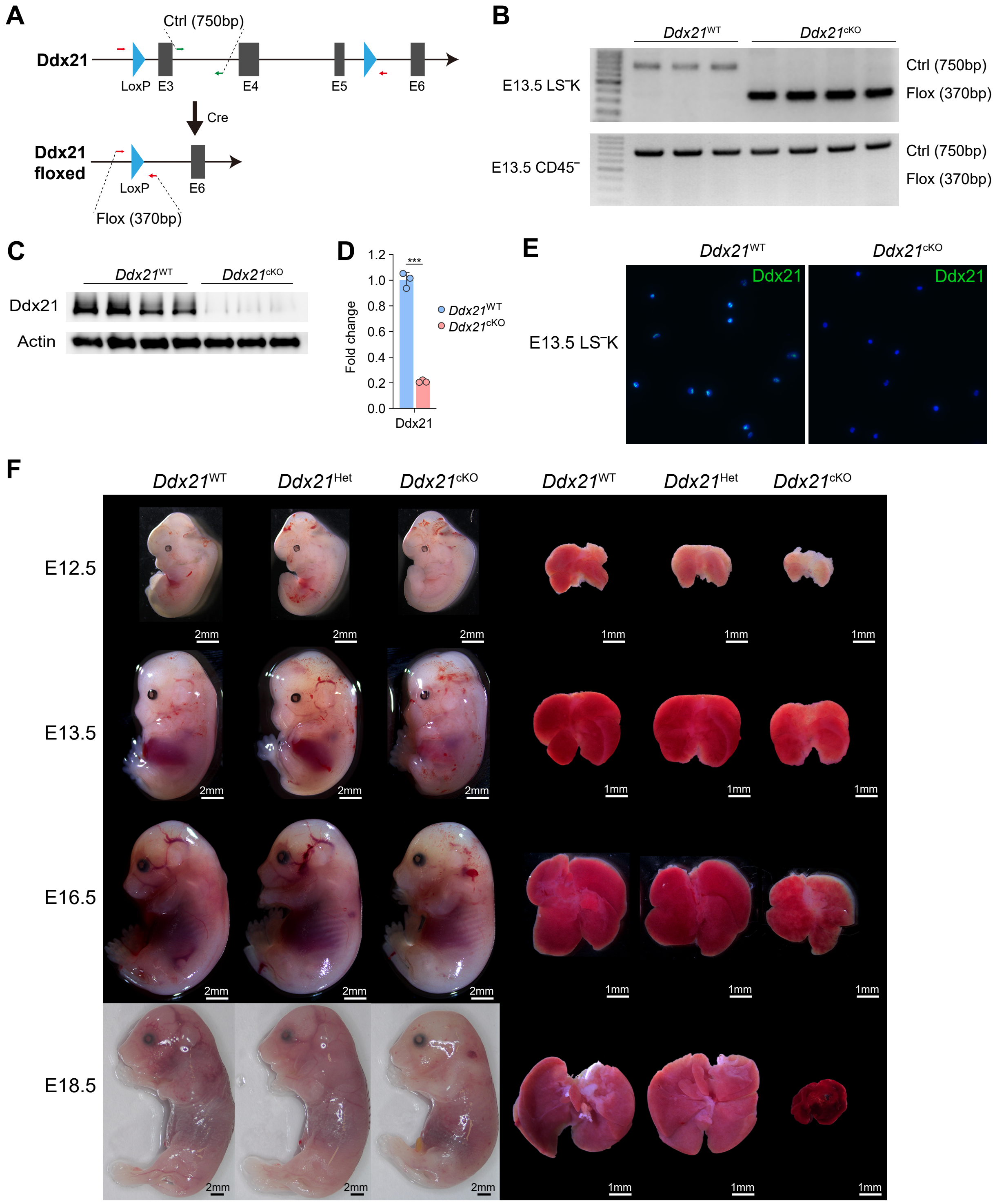

Supplementary figure 1

A, Illustration of *Ddx21*^{flox} knockout allele after Cre recombination. **B**, Genomic Ddx21 knockout efficiency by genomic PCR, demonstrating complete and specific knockout of Ddx21 in fetal liver LS⁻K cells with no deletion in non-hematopoietic cells (CD45⁻) fetal liver cells of E13.5 *Ddx21*^{cKO} embryos (n=3-4). **C**, Ddx21 depletion in E13.5 *Ddx21*^{cKO} fetal liver LS⁻K cells by western blot (n=3-4). **D**, Ddx21 RNA reduction in E13.5 *Ddx21*^{cKO} fetal liver LS⁻K cells by RT-qPCR (n=3). **E**, Ddx21 immunofluorescence staining of sorted fetal liver LK cells of E13.5 *Ddx21*^{WT} and *Ddx21*^{cKO} embryos. **F**, Gross embryo and fetal liver morphology of E12.5, E13.5, E16.5 and E18.5 *Ddx21*^{WT}, *Ddx21*^{Het} and *Ddx21*^{cKO} embryos (n=3-6).
*:<0.05 **:<0.01 ***: <0.001

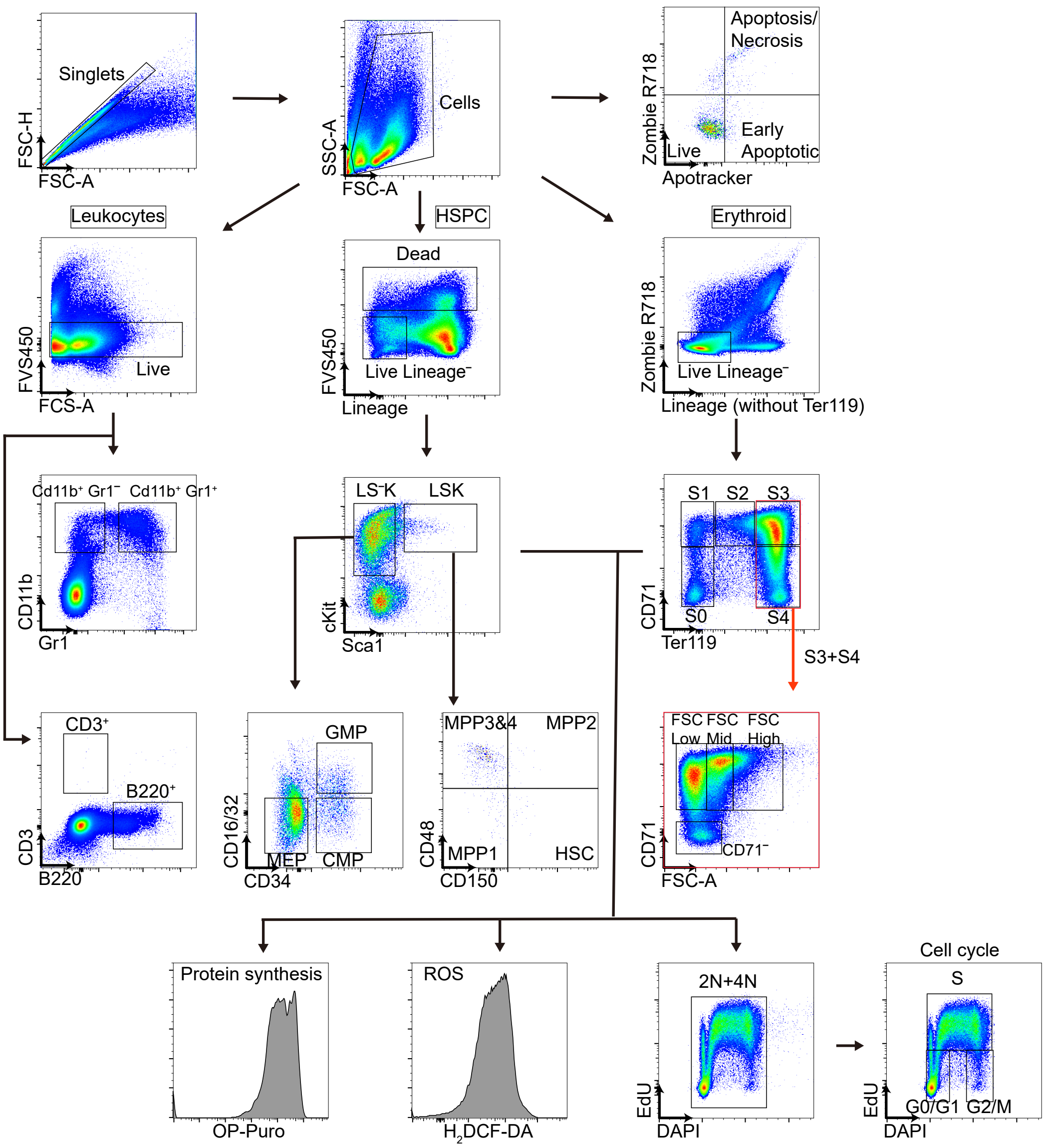

Supplementary figure 2

Gating strategies for HSPC, erythroids and leukocyte population analysis in fetal liver, along with click-iT alexa fluor 647 detection of 2′,7′-Dichlorodihydrofluorescein diacetate (H_2DCF-DA) to measure reactive oxygen species (ROS), O-Propargyl-Puromycin (OP-Puro) to measure nascent protein synthesis and 5-ethynyl-2′-deoxyuridine (EdU) for cell cycle analysis after subsequent staining with 4′,6-diamidino-2-phenylindole (DAPI).

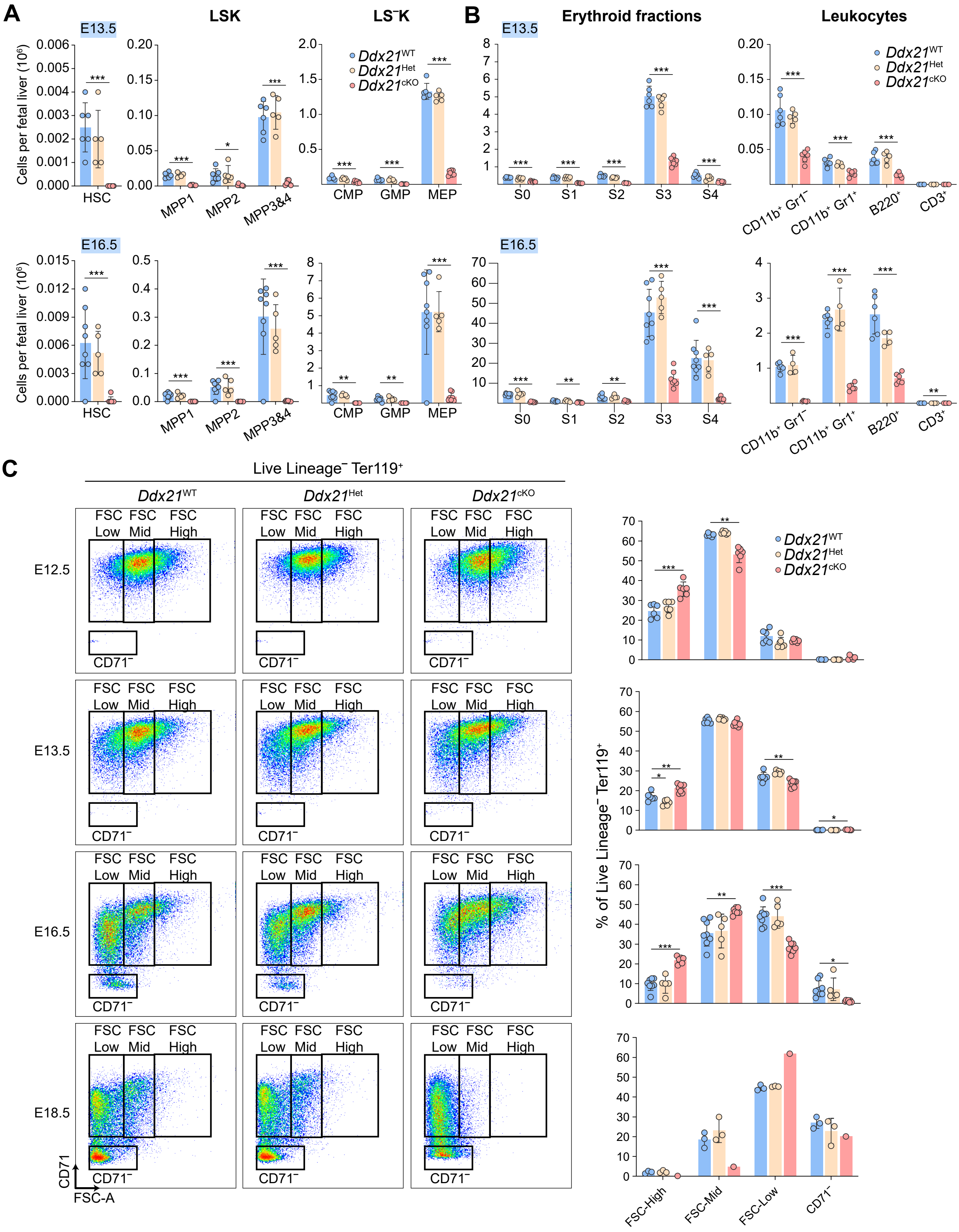

Supplementary figure 3

A, Absolute number of hematopoietic stem and progenitor populations of E13.5 and E16.5 *Ddx21*^{WT}, *Ddx21*^{Het} and *Ddx21*^{cKO} fetal liver by flow cytometry. **B**, Absolute number of erythroid fractions and leuckotyes of E13.5 and E16.5 *Ddx21*^{WT}, *Ddx21*^{Het} and *Ddx21*^{cKO} fetal liver by flow cytometry. **C**, CD71 and FSC-A flow cytomery analysis of fetal liver terminal erythroids (Live Lineage$^-$ Ter119$^+$) differentiation of E12.5 to E18.5 *Ddx21*^{WT}, *Ddx21*^{Het} and *Ddx21*^{cKO} embryos (n=1-8). Representative flow plots are shown. *:<0.05 **:<0.01 ***: <0.001

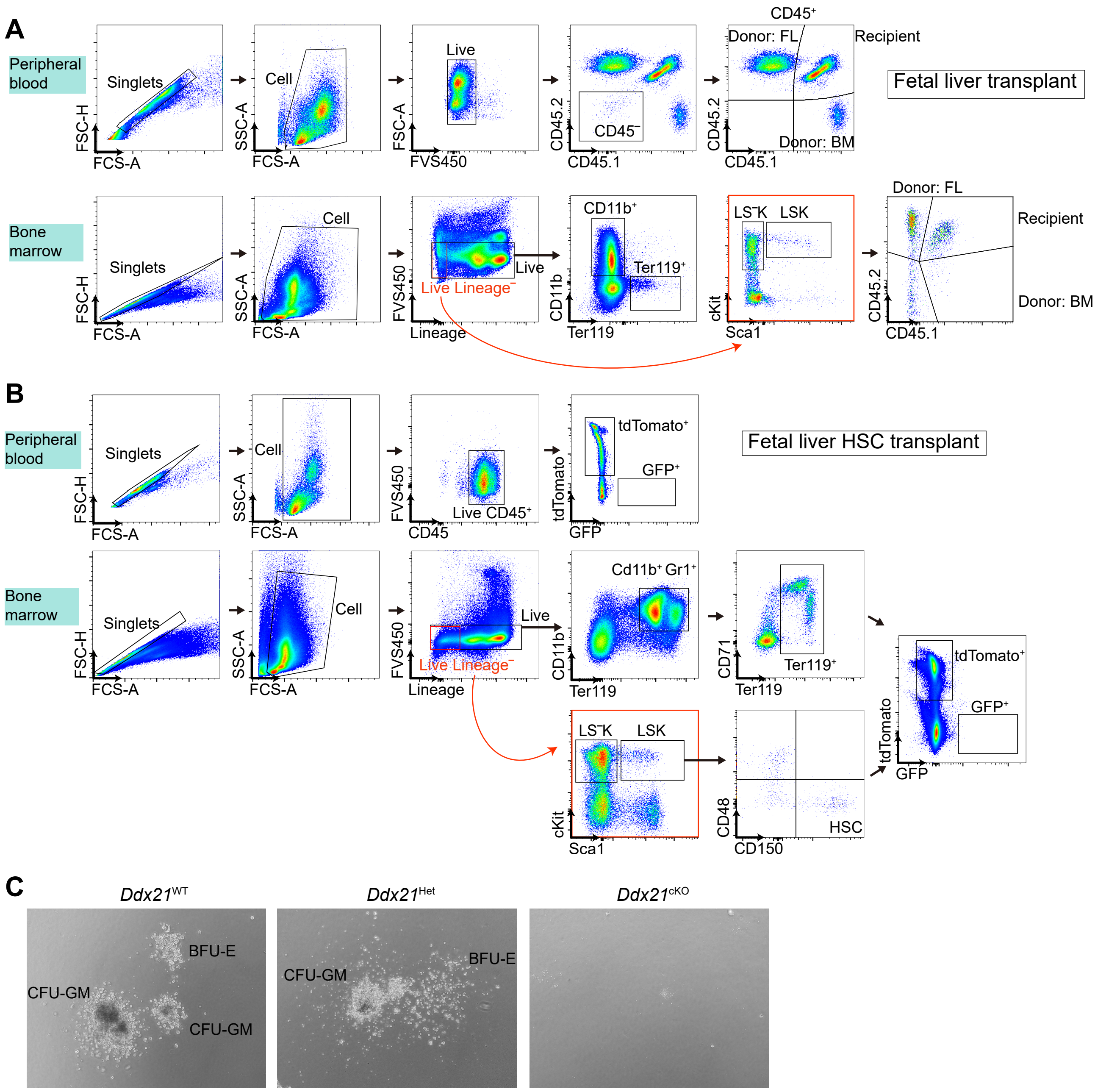

Supplementary figure 4

A, Gating strategies of peripheral blood and bone marrow analysis of mice transplanted with whole fetal liver cells. **B**, Gating strategies of peripheral blood and bone marrow analysis of mice transplanted with mTmG$^{f/+}$ fetal liver HSC. **C**, Representative images of colonies generated by CFU assay (methocult M3434) from E13.5 fetal liver cells.

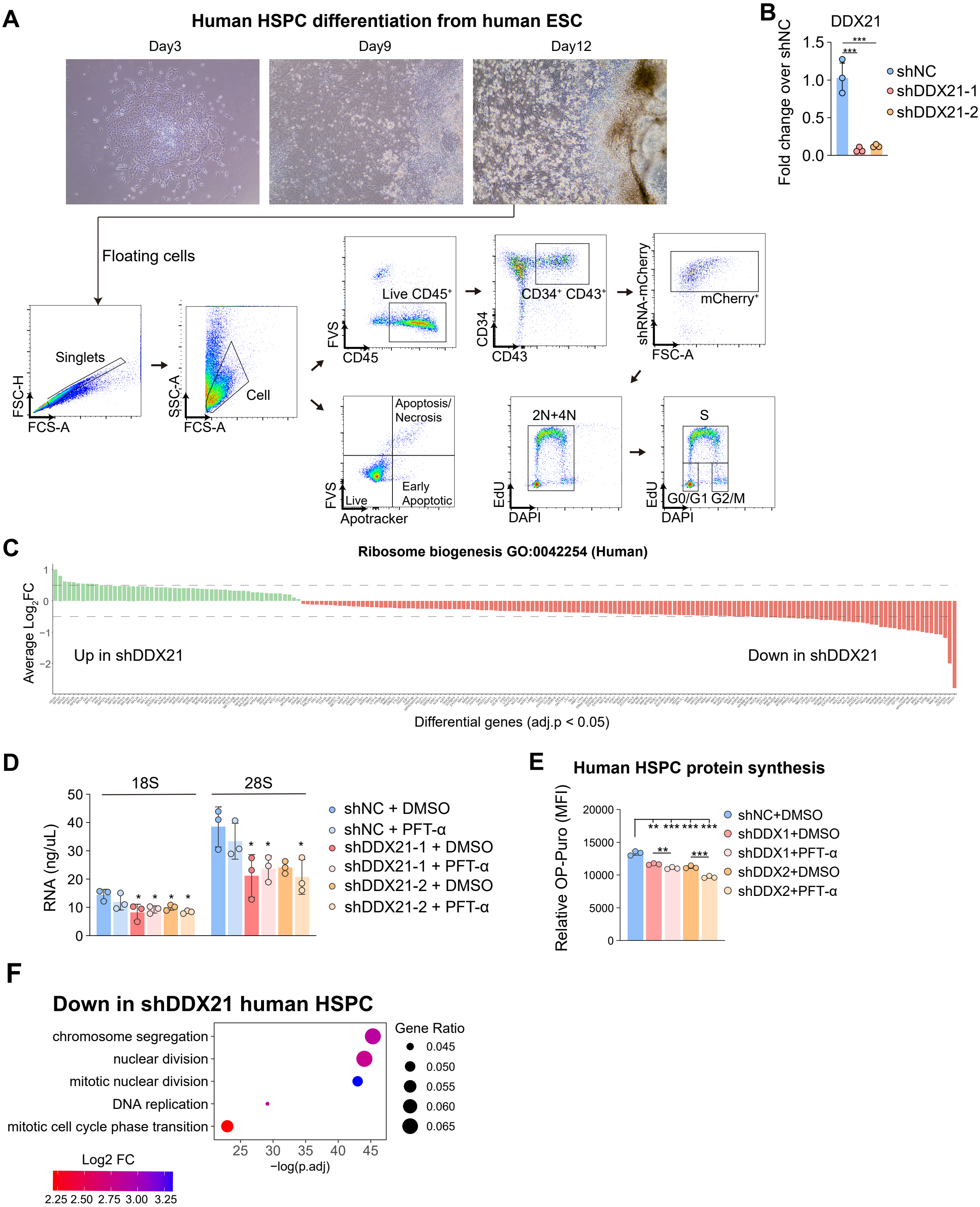

Supplementary figure 5

A, Brightfield images and gating strategies of human HSPC differentiation from human ESC. **B**, RT-qPCR knockdown efficiency of DDX21 in human HSPC. **C**, Average Log_2 fold change of significant differential genes (adj.p <0.05) between shNC and shDDX21 human HSPCs in ribosome biogenesis GO term (0042254). **D**, RNA tapestation analysis of human HSPC after 3 days of shDDX21 lentirivirus transduction and 15μM Pifithrin-α (PFT-α) incubation. **E**, OP-Puro geometric mean fluorescence of human HSPC after 2 days of knockdown and culture in 15μM Pifithrin-α (PFT-α). **F**, GO enrichment analysis of cell cycle related terms in human HSPC transcriptome comparing between shNC and shDDX21.
*:<0.05 **:<0.01 ***: <0.001

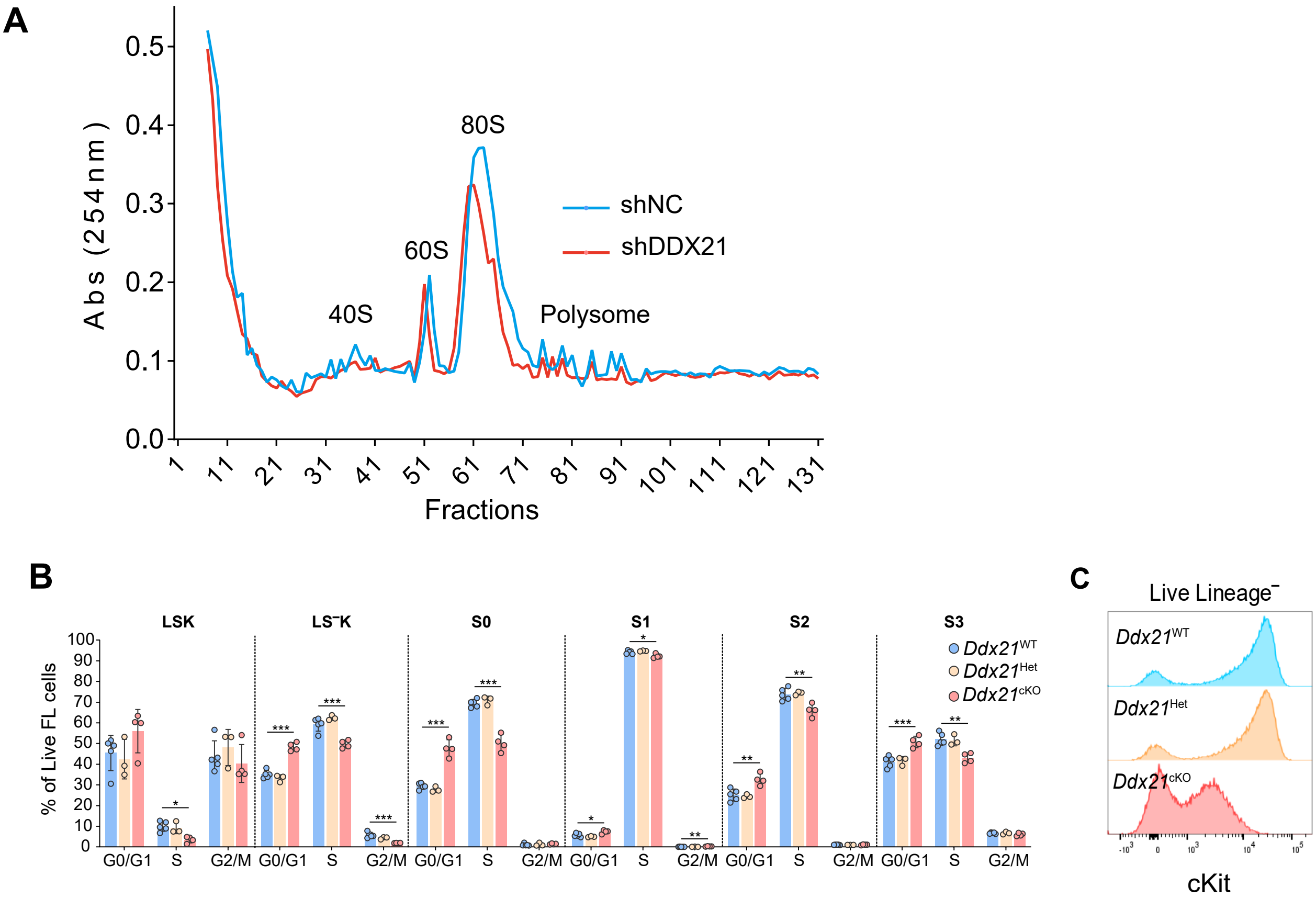

Supplementary figure 6

A, Polysome profiling of shNC and shDDX21 K562 cells. **B**, EdU/DAPI cell cycle analysis of E13.5 fetal liver cells 0.5hr post-EdU injection into pregnant mice. Statistical test compared against *Ddx21*$^{WT.}$ **C**, Flow plot of cKit fluorescence in Live Lineage^{-} cells of E13.5 fetal liver.
*:<0.05 **:<0.01 ***: <0.001

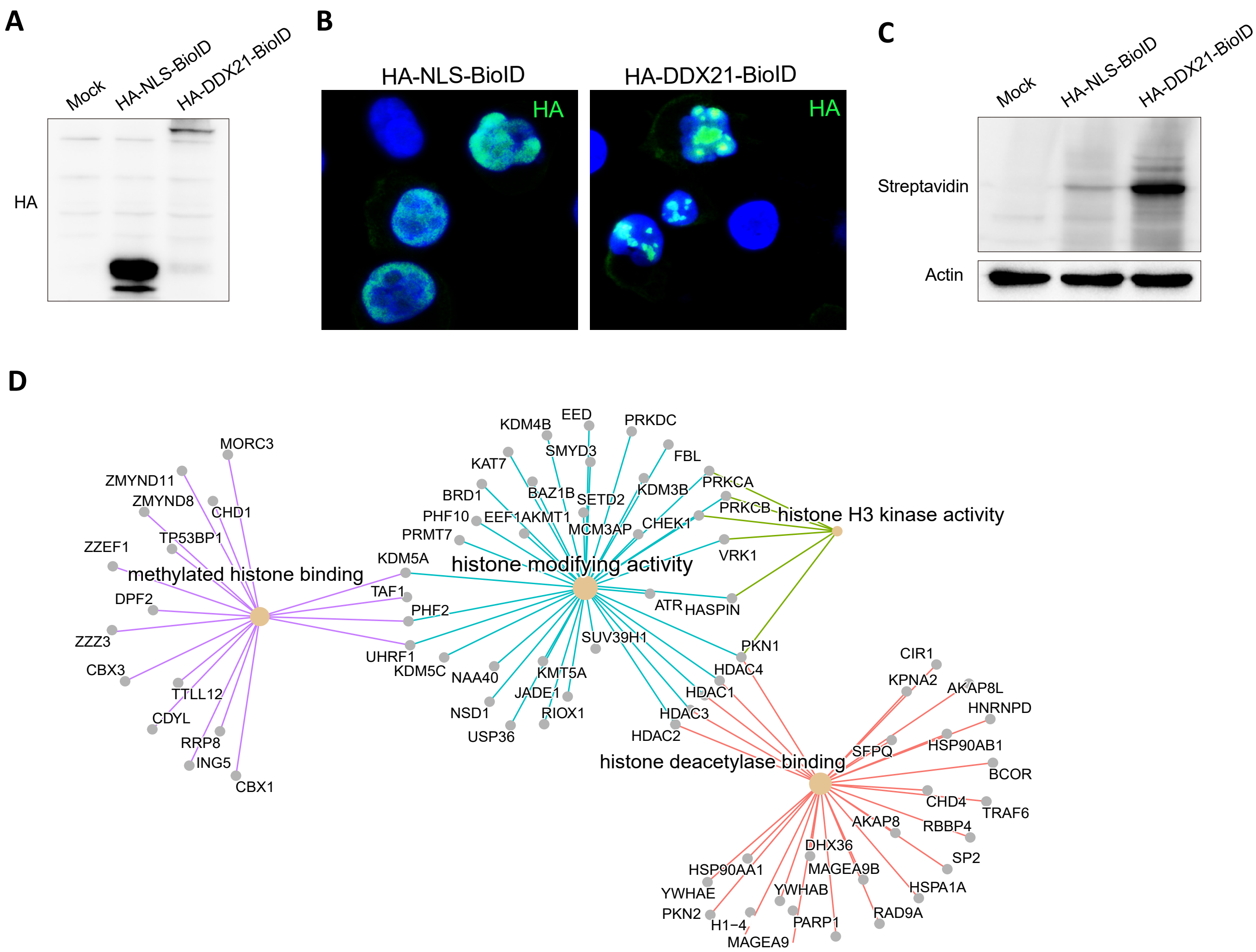

Supplementary figure 7

A-D, DDX21-BioID2 interactome analysis. K562 cells were transducted with either HA-NLS-BioID or HA-DDX21-BioID lentivirus. Cells were expanded for 5 days before biotin was added. Biotinylated protein were subjected to LC-MS/MS (4D-DIA). **A**, HA western blot showing expression of BioID fusion proteins. **B**, HA immunofluorescence showing nuclear localization of BioID fusion proteins. **C,** Streptavidin western blot of whole cell lysate after 1 day of biotin incubation. **D**, Molecular function GO terms related to histone modifications were enriched in the DDX21 interactome.

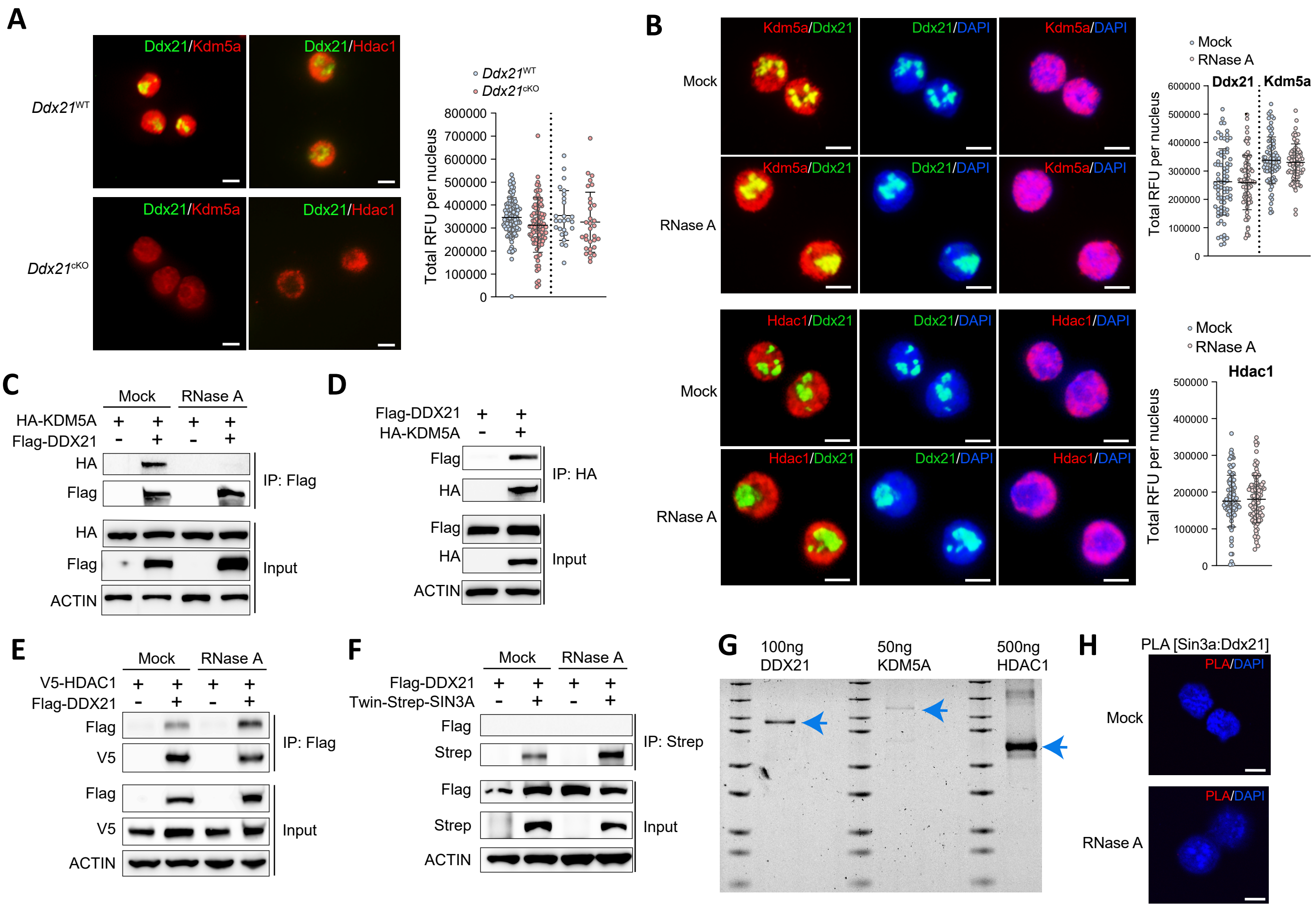

Supplementary figure 8

A, Ddx21, Kdm5a and Hdac1 immunofluorescence in *Ddx21*^{WT} and *Ddx21*^{cKO} E13.5 fetal liver LK cells. Total relative fluorescence within the nucleus was quantified (n=89-108). Scale bar = 5μm. **B**, Immunofluorescence of Ddx21, Kdm5a and Hdac1 in Mock and RNase A treated E13.5 fetal liver LK cells. Total relative fluorescence within the nucleus was quantified (n=80-106). Scale bar = 5μm. **C-F**, Co-immunoprecipitation (Co-IP) blots in which negative control indicates transfection with a mock (empty) plasmid carrying respective tags. **c**, Co-IP of HA-KDM5A with Flag-DDX21 in 293F with or without RNase A treatment. **D**, Co-IP of Flag-DDX21 with HA-KDM5A in 293F. **E**, Co-IP of V5-HDAC1 with Flag-DDX21 in 293F with or without RNase A treatment. **F**, Co-IP of Flag-DDX21 with twin-strep-SIN3A in 293F with or without RNase A treatment. **G**, Coomassie blue staining of purified DDX21, KDM5A and HDAC1 proteins. **H**, Proximity ligation assay (PLA) staining between Ddx21 and Sin3a in mock or RNase A-treated E13.5 fetal liver LK cells (n=31-53). Scale bar = 5μm.

*:<0.05 **:<0.01 ***: <0.001

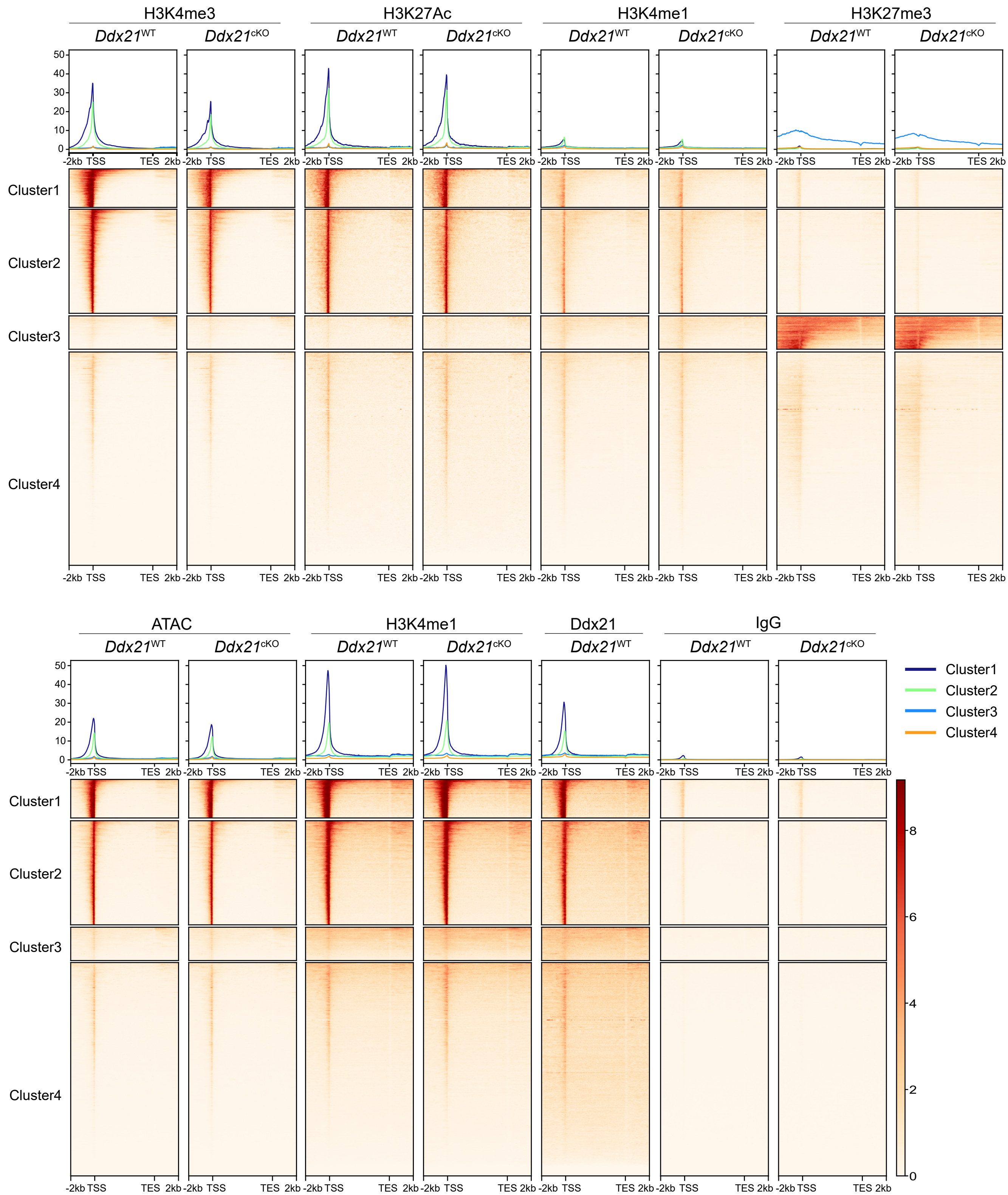

Supplementary figure 9

Profile and heatmap plot of TMM-background bin normalized and per-million scaled CUT&Tag signals of H3K4me3, H3K4me1, H3K27me3, H3K27Ac, Kdm5a, Ddx21,IgG and ATAC-seq of all genebodies in E13.5 *Ddx21*^{WT} and *Ddx21*^{cKO} fetal liver LK cells. TSS: Transcription start site. TES: Transcription end site.

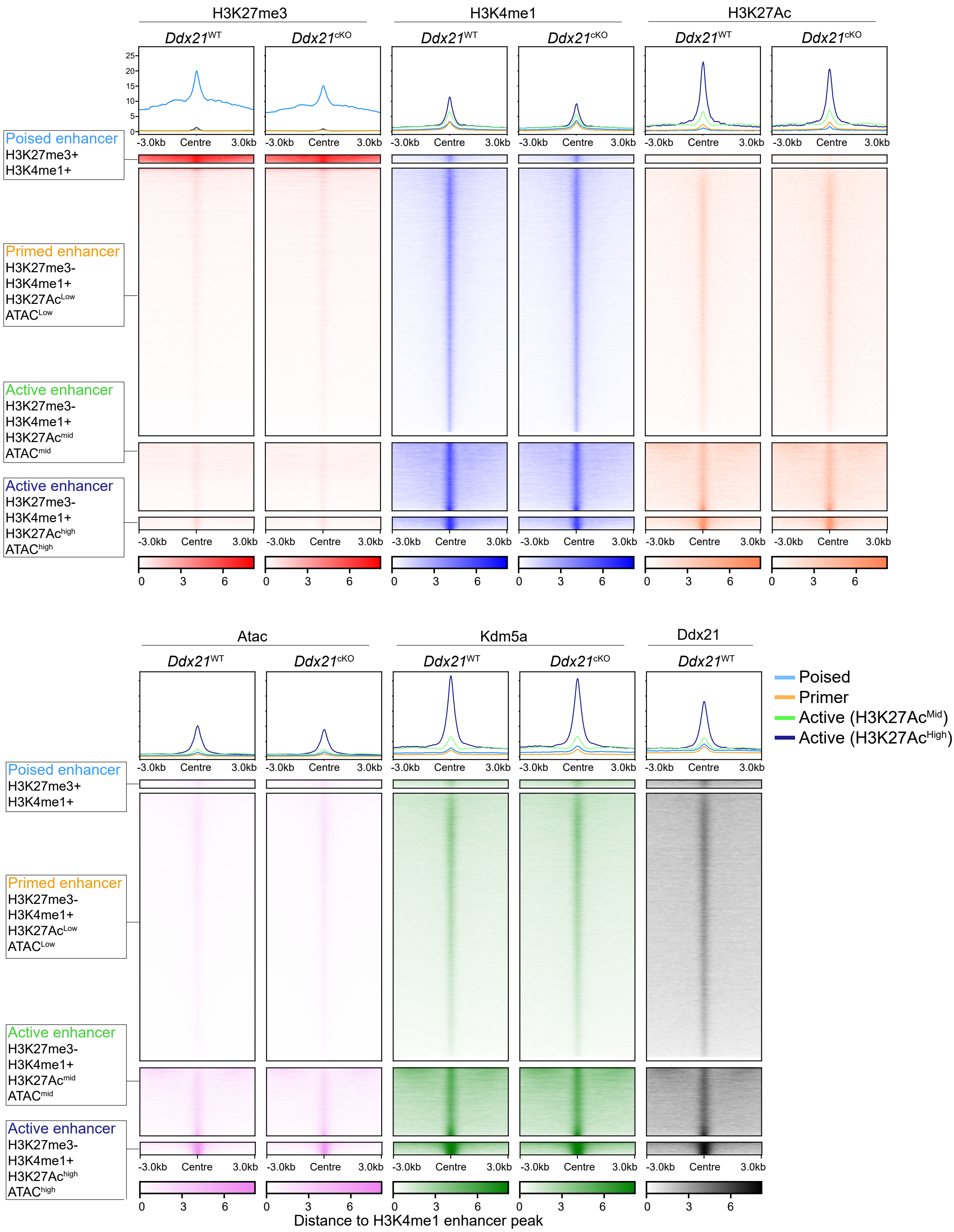

Supplementary figure 10

Profile and heatmap plot of TMM-background bin normalized and per-million scaled CUT&Tag and ATAC signals in H3K4me1+ enhancer peaks (H3K4me1+ peaks in promoter were excluded). Enhancers were grouped to poised, primed and active enhancers based on combinatorial histone and ATAC binding (Barral A et al., 2023).

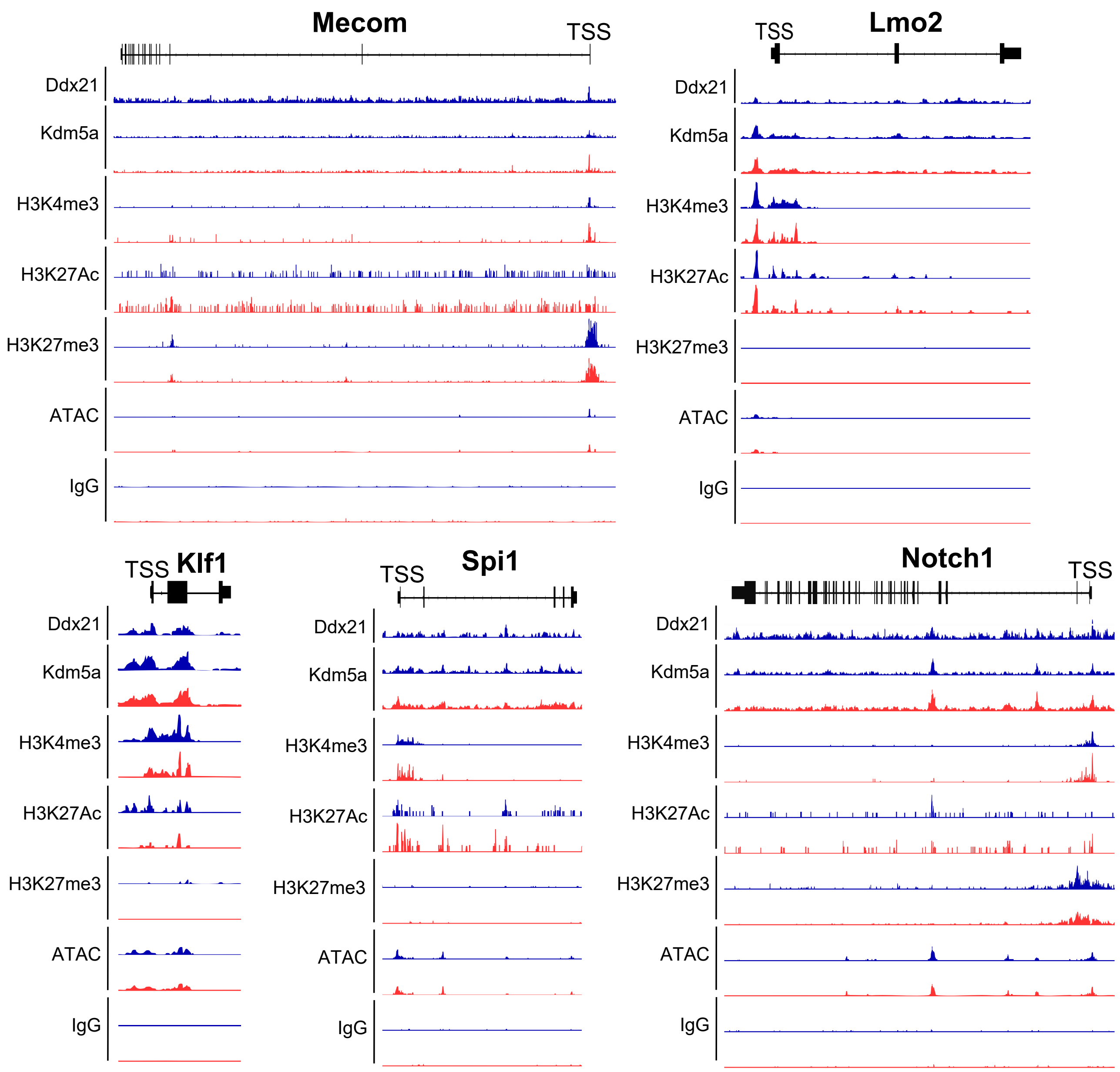

Supplementary figure 11
Epigenetic tracks of example stemness, progenitor, myeloid and lymhoid tracks of H3K4me3, H3K27me3, H3K27Ac, Ddx21 and Kdm5a by CUT&Tag-seq, and open chromatin by ATAC-seq in E13.5 fetal liver Lineage–cKit+ (LK) cells of *Ddx21*^{WT} and *Ddx21*^{cKO} embryos (n=2, biological replicates). Signals of the same target were normalized, scaled to per-million and merged between replicates.

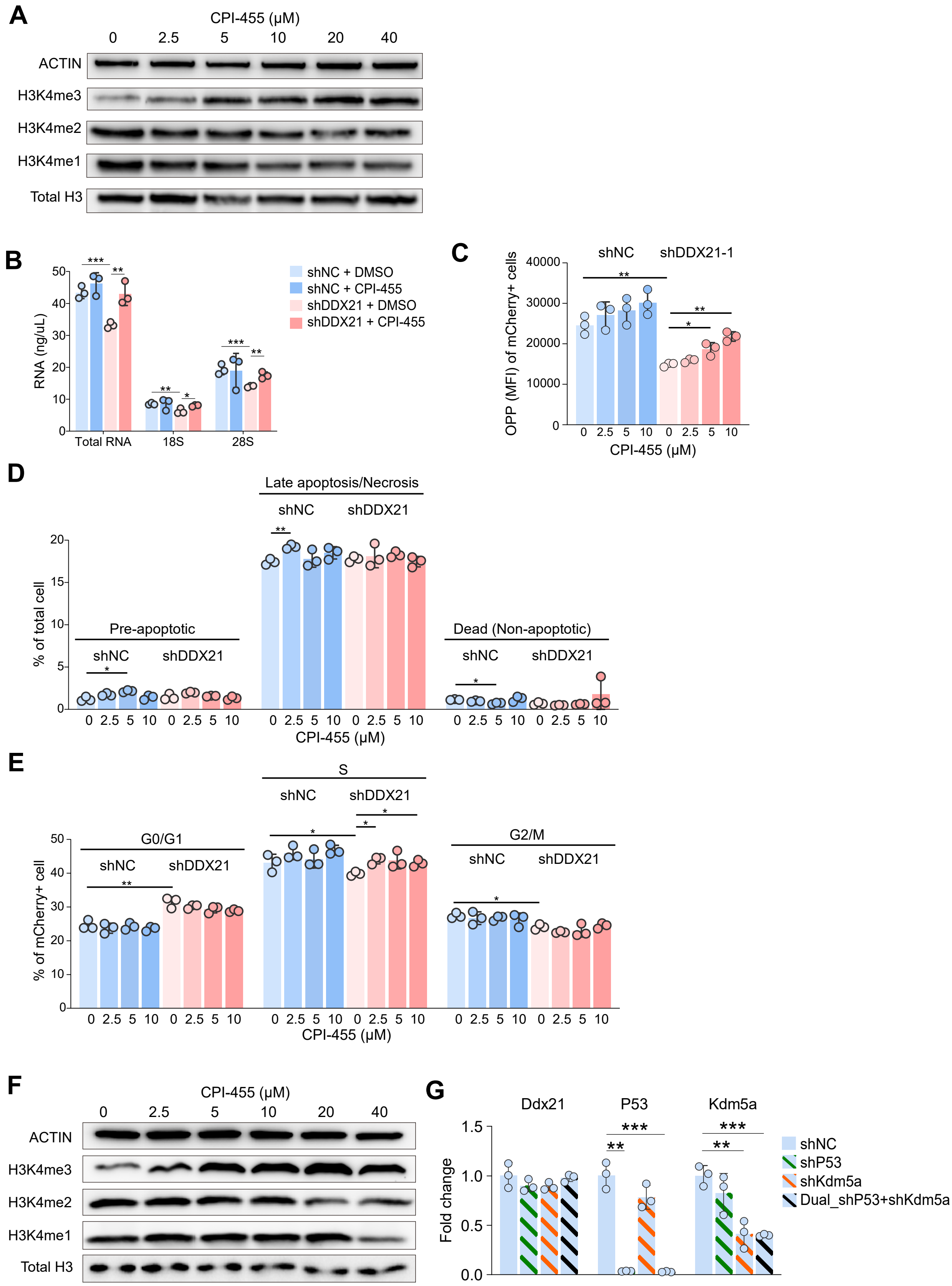

Supplementary figure 12

A, Western blot of H3K4me3, H3k4me2, H3K4me1, total H3 and β-Actin after 1 day of CPI-455 treatment in K562 cells. **B**, TapeStation rRNA analysis of K562 cells 2 days post transduction with shRNA lentivirus and culture in 10μM CPI-455/DMSO (n=3). **C-E**, Flow cytometry analysis of K562 cells 2 days post lentiviral transduction and culture with increasing dose of CPI455/DMSO (n=3). **C**, OP-Puro nascent protein synthesis analysis. **D,** apoptosis/cell death analysis. **E,** EdU/DAPI cell cycle analysis. **F**, Western blot of H3K4me3, H3k4me2, H3K4me1, total H3 and β-Actin after 1 day of CPI-455 treatment in cultured E13.5 LK cells. **G**, RT-qPCR analysis of shRNA knockdown efficiency in cultured E13.5 fetal liver LK cells after 3 days of lentiviral transduction carrying either single or double U6-shRNA construct (n=3).
*:<0.05 **:<0.01 ***: <0.001

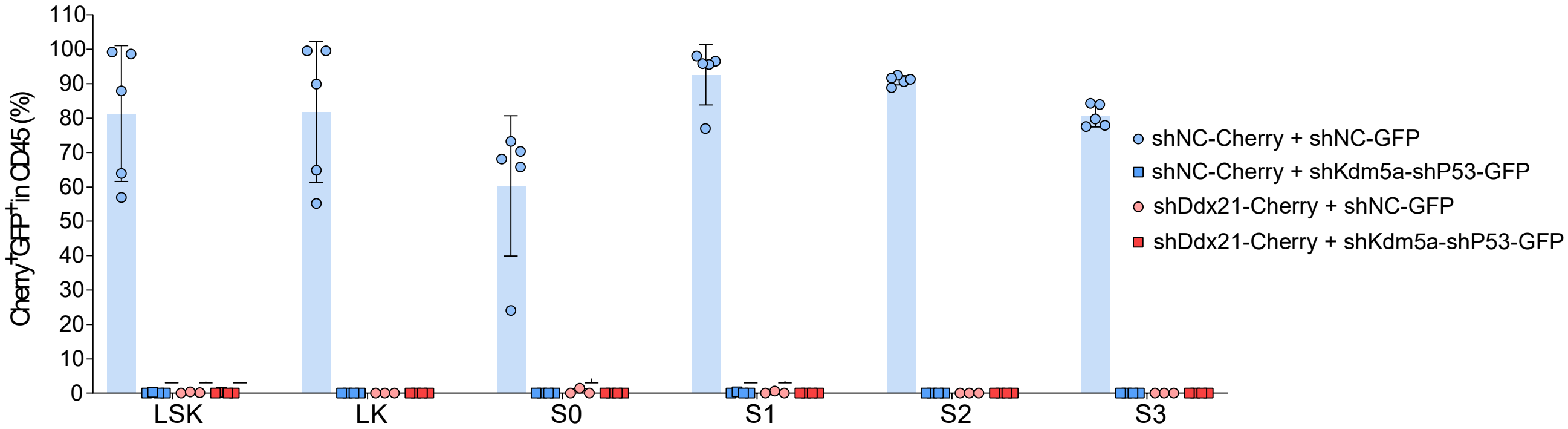

Supplementary figure 13

In vivo rescue of transplanted shDdx21 fetal liver cells after lentivirus transfection. Flow cytometry analysis of BM HSPC and erythroid cells at 12 weeks post-transplantation.
