# Supplementary material for: Non‐Canonical Function of DDX21 in Fetal Hematopoiesis Antagonizes KDM5A‐Mediated Repression of Ribosomal and Hematopoietic Genes
Source: Adv Sci (Weinh). 2026 Nov 9:e78524. Online ahead of print. doi: 10.1002/advs.78524 (PMC13748770; doi:10.1002/advs.78524)
Supplement: Supplementary file 2 [file ADVS-9999-e78524-s002.pdf]

	Cells per fetal liver (10^6)					
	E12.5			E13.5		
	**WT** (n=6)	**Het** (n=6)	**cKO** (n=6)	**WT** (n=6)	**Het** (n=6)	**cKO** (n=6)
LSK	0.013 ± 0.001	0.019 ± 0.003	0.018 ± 0.002	0.13 ± 0.01	0.14 ± 0.01	0.007 ± 0.002
HSC	0.000 ± 0.00005	0.000 ± 0.0001	0.001 ± 0.0001	0.002 ± 0.0004	0.002 ± 0.0004	0.000 ± 0.0001
MPP1	0.003 ± 0.0005	0.003 ± 0.0003	0.003 ± 0.0005	0.015 ± 0.0015	0.016 ± 0.0014	0.001 ± 0.0002
MPP2	0.001 ± 0.0002	0.001 ± 0.0002	0.003 ± 0.0004	0.016 ± 0.004	0.017 ± 0.005	0.001 ± 0.0005
MPP3&4	0.008 ± 0.001	0.014 ± 0.003	0.012 ± 0.002	0.098 ± 0.009	0.104 ± 0.011	0.004 ± 0.001
LKS^{-}	0.667 ± 0.049	0.721 ± 0.045	0.616 ± 0.043	1.92 ± 0.06	1.81 ± 0.04	0.22 ± 0.02
CMP	0.077 ± 0.006	0.091 ± 0.007	0.127 ± 0.013	0.094 ± 0.009	0.079 ± 0.007	0.033 ± 0.006
GMP	0.031 ± 0.003	0.048 ± 0.004	0.030 ± 0.002	0.069 ± 0.007	0.064 ± 0.007	0.005 ± 0.001
MEP	0.442 ± 0.037	0.439 ± 0.029	0.381 ± 0.033	1.33 ± 0.05	1.26 ± 0.03	0.17 ± 0.01
S0	0.178 ± 0.034	0.206 ± 0.036	0.213 ± 0.041	0.39 ± 0.01	0.33 ± 0.02	0.16 ± 0.01
S1	0.247 ± 0.079	0.139 ± 0.019	0.121 ± 0.023	0.38 ± 0.02	0.38 ± 0.02	0.068 ± 0.005
S2	0.365 ± 0.100	0.308 ± 0.035	0.152 ± 0.035	0.49 ± 0.03	0.38 ± 0.01	0.067 ± 0.006
S3	1.481 ± 0.039	1.507 ± 0.107	0.993 ± 0.218	5.02 ± 0.24	4.73 ± 0.15	1.32 ± 0.08
S4	0.029 ± 0.015	0.043 ± 0.003	0.026 ± 0.005	0.51 ± 0.06	0.36 ± 0.05	0.14 ± 0.02
	E16.5			E18.5		
	**WT** (n=8)	**Het** (n=5)	**cKO** (n=7)	**WT** (n=3)	**Het** (n=3)	**cKO** (n=3)
LSK	0.38 ± 0.06	0.34 ± 0.05	0.000 ± 0.0001	0.338 ± 0.018	0.440 ± 0.048	0.000 ± 0.000
HSC	0.006 ± 0.001	0.005 ± 0.001	0.000 ± 0.00002	0.011 ± 0.005	0.007 ± 0.001	0.000 ± 0.000
MPP1	0.023 ± 0.004	0.022 ± 0.004	0.000 ± 0.00002	0.022 ± 0.009	0.033 ± 0.003	0.000 ± 0.000
MPP2	0.051 ± 0.009	0.054 ± 0.013	0.000 ± 0.00003	0.085 ± 0.047	0.027 ± 0.009	0.000 ± 0.000
MPP3&4	0.30 ± 0.05	0.26 ± 0.04	0.000 ± 0.0001	0.22 ± 0.05	0.37 ± 0.04	0.000 ± 0.000
LKS^{-}	7.08 ± 1.14	7.18 ± 0.76	0.40 ± 0.10	3.47 ± 0.41	5.34 ± 0.27	0.00 ± 0.00
CMP	0.46 ± 0.09	0.46 ± 0.03	0.002 ± 0.0004	0.33 ± 0.08	0.36 ± 0.03	0.000 ± 0.000
GMP	0.23 ± 0.04	0.25 ± 0.05	0.00 ± 0.00	0.11 ± 0.02	0.11 ± 0.00	0.00 ± 0.00
MEP	5.21 ± 0.85	5.23 ± 0.51	0.36 ± 0.09	2.53 ± 0.24	4.27 ± 0.20	0.00 ± 0.00
S0	4.43 ± 0.23	4.71 ± 0.57	0.63 ± 0.10	2.18 ± 0.35	2.08 ± 0.22	0.04 ± 0.01
S1	1.07 ± 0.12	1.12 ± 0.20	0.40 ± 0.09	0.26 ± 0.04	0.30 ± 0.01	0.01 ± 0.00
S2	2.98 ± 0.38	3.22 ± 0.41	1.03 ± 0.14	1.55 ± 0.39	1.33 ± 0.08	0.03 ± 0.01
S3	45.20 ± 4.19	52.89 ± 3.60	12.33 ± 1.57	29.75 ± 3.81	33.92 ± 3.00	3.17 ± 0.54
S4	22.64 ± 3.12	21.37 ± 2.53	2.15 ± 0.34	65.01 ± 6.91	62.10 ± 3.08	8.81 ± 0.59

Supplementary table S1

Hematopoietic cell number per fetal liver in $\textit{Ddx21}^{WT}$ (WT), $\textit{Ddx21}^{Het}$ (Het) and $\textit{Ddx21}^{cKO}$ (cKO) embryos. Data are presented as mean ± s.e.m.

	% of live fetal liver cells					
	E12.5			E13.5		
	**WT** (n=6)	**Het** (n=6)	**cKO** (n=6)	**WT** (n=6)	**Het** (n=6)	**cKO** (n=6)
LSK	0.56 ± 0.07	0.77 ± 0.09	1.32 ± 0.07	1.65 ± 0.15	1.91 ± 0.21	0.31 ± 0.08
HSC	0.013 ± 0.002	0.017 ± 0.003	0.044 ± 0.004	0.031 ± 0.005	0.028 ± 0.006	0.009 ± 0.002
MPP1	0.14 ± 0.03	0.11 ± 0.01	0.19 ± 0.02	0.194 ± 0.020	0.219 ± 0.013	0.050 ± 0.009
MPP2	0.055 ± 0.007	0.045 ± 0.006	0.243 ± 0.032	0.200 ± 0.050	0.239 ± 0.074	0.051 ± 0.021
MPP3&4	0.35 ± 0.05	0.59 ± 0.08	0.84 ± 0.10	1.22± 0.11	1.43 ± 0.15	0.20 ± 0.06
LKS¯	28.0 ± 0.95	30.6 ± 0.7	45.0 ± 0.9	1.65 ± 0.95	24.7 ± 0.40	10.7 ± 0.94
CMP	3.24 ± 0.17	3.89 ± 0.31	9.32 ± 0.79	1.19 ± 0.17	1.07 ± 0.08	1.65 ± 0.32
GMP	1.32 ± 0.12	2.07 ± 0.19	2.25 ± 0.21	0.87± 0.12	0.87 ± 0.08	0.26 ± 0.05
MEP	18.5 ± 0.90	18.6 ± 0.38	27.7 ± 0.90	16.7 ± 0.90	17.2 ± 0.52	7.93 ± 0.46
S0	7.21 ± 0.41	8.01 ± 0.57	22.9 ± 2.06	4.86 ± 0.16	4.52 ± 0.24	7.32 ± 0.41
S1	9.55 ± 1.30	6.31 ± 0.31	8.69 ± 1.01	4.78 ± 0.14	5.16 ± 0.24	3.24 ± 0.20
S2	14.2 ± 1.52	12.1 ± 0.64	8.82 ± 0.60	6.15 ± 0.31	5.24 ± 0.15	3.17 ± 0.19
S3	62.4 ± 2.65	66.8 ± 1.40	51.2 ± 3.76	62.9 ± 1.33	64.7 ± 0.78	62.2 ± 0.58
S4	1.39 ± 0.41	1.76 ± 0.20	1.98 ± 0.20	6.44 ± 0.76	4.92 ± 0.62	6.49 ± 0.69
	E16.5			E18.5		
	**WT** (n=8)	**Het** (n=5)	**cKO** (n=7)	**WT** (n=3)	**Het** (n=3)	**cKO** (n=3)
LSK	0.490 ± 0.020	0.352 ± 0.051	0.002 ± 0.0006	0.329 ± 0.041	0.414 ± 0.037	0.000 ± 0.000
HSC	0.009 ± 0.001	0.005 ± 0.001	0.000 ± 0.0001	0.010 ± 0.005	0.007 ± 0.001	0.000 ± 0.000
MPP1	0.031 ± 0.003	0.023 ± 0.004	0.000 ± 0.0001	0.023 ± 0.011	0.032 ± 0.004	0.000 ± 0.000
MPP2	0.068 ± 0.010	0.055 ± 0.012	0.000 ± 0.0001	0.074 ± 0.040	0.025 ± 0.008	0.000 ± 0.000
MPP3&4	0.38 ± 0.009	0.27 ± 0.03	0.002 ± 0.0006	0.223 ± 0.074	0.351 ± 0.032	0.000 ± 0.000
LKS¯	9.21 ± 0.41	7.54 ± 0.74	2.36 ± 0.61	3.29 ± 0.08	5.04 ± 0.23	0.00 ± 0.00
CMP	0.58 ± 0.05	0.48 ± 0.02	0.009 ± 0.002	0.300 ± 0.054	0.336 ± 0.033	0.000 ± 0.000
GMP	0.32 ± 0.04	0.26 ± 0.05	0.007 ± 0.001	0.100 ± 0.015	0.105 ± 0.005	0.000 ± 0.000
MEP	6.70 ± 0.32	5.49 ± 0.51	2.16 ± 0.58	2.42 ± 0.16	4.04 ± 0.15	0.00 ± 0.00
S0	5.05 ± 0.23	4.86 ± 0.38	3.31 ± 0.19	2.04 ± 0.15	1.96 ± 0.18	0.27 ± 0.05
S1	1.25 ± 0.16	1.20 ± 0.24	2.42 ± 0.60	0.26 ± 0.05	0.29 ± 0.01	0.04 ± 0.01
S2	3.32 ± 0.28	3.42 ± 0.52	5.53 ± 0.19	1.55 ± 0.48	1.25 ± 0.05	0.21 ± 0.03
S3	50.87 ± 2.98	55.15 ± 1.79	66.50 ± 2.66	28.13 ± 0.87	32.00 ± 2.32	22.40 ± 1.17
S4	25.38 ± 2.60	22.15 ± 2.03	12.19 ± 2.05	61.77 ± 0.22	58.73 ± 3.00	64.03 ± 4.29

Supplementary table S2

Percentage of hematopoietic cells per fetal liver in *Ddx21*^{WT} (WT), *Ddx21*^{Het} (Het) and *Ddx21*^{cKO} (cKO) embryos. Data are presented as mean ± s.e.m.

Purpose	F	R	Tm	Size (bp)
Vav1-Cre genotyping	GGTGTTGTAGTTGTCCCCACT	CAGGTTTTGGTGCACAGTCA	60	Cre: 390<br>WT: 212
Ddx21^{flox} genotyping	GAACTCAGAGATCTGCCTCTCAAAT	CCGAGTGTTCTCTTGTGCTGTA	60	flox: 201<br>WT: 144
mTmG^{flox} genotyping	AGGGAGCTGCAGTGGAGTAG<br>TAGAGCTTGCGGAACCCTTC	CTTTAAGCCTGCCCAGAAGA	60	flox: 128<br>WT: 212
Ddx21^{flox} KO efficiency	GAACTCAGAGATCTGCCTCTCAAAT	CATAAGAAAGTGAGAGGGCACAGA	63	Delta: 370
	GTATCAGGGAGCAAACCCCA	TTGTGCGGTTTGAGGTCAGT	63	Flox: 750

Supplementary table S3

Primers used for mouse genotyping and Ddx21^{flox} KO efficiency test

Name	Function	Stem	Loop
shNC	shRNA control	CCTAAGGTTAAGTCGCCCTCG	CCTGACCCA
shDdx21	Mouse Ddx21 KD	GTCTCCAGACAAGGGTATATA	CCTGACCCA
shP53	Mouse P53 KD	GTACATGTGTAATAGCTCC	CCTGACCCA
shKdm5a	Mouse Kdm5a KD	CGAGGAAATAATGAGGATAAA	CCTGACCCA
shDDX21-1	Human DDX21 KD	GCCCATATCTGAAGAAACTATT	CCTGACCCA
shDDX21-2	Human DDX21 KD	GCATGAGGAATGGGATTG	ATACTCGAGTAT
shDDX21-3 (3'-UTR)	Human DDX21 KD	GGTGGCTCCATAGCTTTATT	CCTGACCCA
shHuP53	Human P53 KD	GACTCCAGTGGTAATCTAC	CCTGACCCA

Supplementary table S4

Stem and loop sequences of shRNAs

**Lineage-biotin panel for fetal liver population analysis**<br>For erythroid fraction analysis, Ter119-biotin is omitted and zombie-R718 is used				
**Target**	**Conjugate**	**Cat no.**	**Manufacturer**	**Dilution**
CD3*	Biotin	100303	Biolegend	1:125
B220*	Biotin	103203	Biolegend	1:125
Gr1*	Biotin	108403	Biolegend	1:125
CD11b*	Biotin	101203	Biolegend	1:125
Ter119*	Biotin	116203	Biolegend	1:125
Viability* (HSC/leukocyte)	FVS450	562247	BD	1:250
Viability (erythroid)	Zombie-R718	423115	Biolegend	1:250

**Fetal liver HSPC surface panel**				
**Target**	**Conjugate**	**Cat no.**	**Manufacturer**	**Dilution**
Biotin*	Streptavidin-APC/Cy7	405208	Biolegend	1:125
cKit*	APC	135107	Biolegend	1:125
Sca1*	BV605	108133	Biolegend	1:125
CD48*	PE	103405	Biolegend	1:125
CD150*	BV510	115929	Biolegend	1:125
CD16/32	PerCP/Cy5.5	101323	Biolegend	1:125
CD34	FITC	11-0341-81	Life Technologies	1:125

**Fetal liver erythroid fraction surface panel**				
**Target**	**Conjugate / dye**	**Cat no.**	**Manufacturer**	**Dilution**
Biotin (lineage)	Streptavidin-APC/Cy7	405208	Biolegend	1:125
CD71	BV510	113823	Biolegend	1:125
Ter119	PE/Cy7	116221	Biolegend	1:125
DNA	Hoechst33342	H1399	Life Technologies	1ug/ml
ROS	H_2DCF-DA	D399	Life Technologies	10μM

**Fetal liver leukocytes surface panel**				
**Target**	**Conjugate**	**Cat no.**	**Manufacturer**	**Dilution**
CD3	PE	100307	Biolegend	1:125
B220	APC/Cy7	103223	Biolegend	1:125
Gr1	APC	108411	Biolegend	1:125
CD11b	FITC	101205	Biolegend	1:125

**Apoptosis**				
**Target**	**Conjugate**	**Cat no.**	**Manufacturer**	**Dilution**
Apoptotic cell	Apotracker Green	427402	Biolegend	1:125
Dead	FVS450	562247	BD	1:250

**Cell cycle Click-iT (After surface stain, fix, permeabilize)**				
**Target**	**Conjugate**	**Cat no.**	**Manufacturer**	**Dilution**
EdU	Alexa fluor 647	C10419	Life Technologies	As in manual
DNA	DAPI	D1306	Life Technologies	0.1ug/ml

**Nascent protein synthesis Click-iT (After surface stain, fix, permeabilize)**				
**Target**	**Conjugate**	**Cat no.**	**Manufacturer**	**Dilution**
OP-Puro	Alexa fluor 647	C10458	Life Technologies	As in manual

Supplementary table S5

All antibodies, reagents and dyes used in flow cytometry and sorting for fetal liver population analysis, apoptosis and cell cycle. Antibodies used for FACS sorting of live LK cells and HSC were marked (*).

CD45.1/CD45.2 transplantation analysis and HSC sorting for transplantation				
**Target**	**Conjugate**	**Cat no.**	**Manufacturer**	**Dilution**
**Common**				
CD45.1	PE/Cy7	110729	Biolegend	1:125
CD45.2	FITC	109805	Biolegend	1:125
Dead	FVS450	562247	BD	1:250
**Peripheral blood**				
CD3	PerCP	100325	Biolegend	1:125
B220	APC/Cy7	103223	Biolegend	1:125
CD11b	Spark UV387	101291	Biolegend	1:125
**Bone marrow HSPC + Ter119 + Cd11b (Stained with lineage-biotin panel first)**				
cKit	APC	135107	Biolegend	1:125
Sca1	BV605	108133	Biolegend	1:125
CD48	PE	103405	Biolegend	1:125
CD150	BV510	115929	Biolegend	1:125
Ter119	PerCP	116225	Biolegend	1:125
CD11b	SparkUV387	101291	Biolegend	1:125
Biotin (lineage)	Streptavidin-APC/Cy7	405208	Biolegend	1:125

mTmG^{flox} transplantation analysis				
**Target**	**Conjugate**	**Cat no.**	**Manufacturer**	**Dilution**
**Common**				
	GFP			
	Tdtomato			
Dead	FVS450	562247	BD	1:250
**Peripheral blood**				
CD45	APC	103111	Biolegend	1:125
CD3	PerCP	100325	Biolegend	1:125
B220	APC/Cy7	103223	Biolegend	1:125
CD11b	Spark UV387	101291	Biolegend	1:125
**Bone marrow HSPC (Stained with lineage-biotin panel first)**				
cKit	APC	135107	Biolegend	1:125
Sca1	BV605	108133	Biolegend	1:125
CD16/32	PerCP/Cy5.5	101323	Biolegend	1:125
CD34	AF700	560518	BD	1:125
CD48	PE/Cy7	103423	Biolegend	1:125
CD150	BV510	115929	Biolegend	1:125
Biotin (lineage)	Streptavidin-APC/Cy7	405208	Biolegend	1:125
**Bone marrow erythroid fractions and myeloids (Stained with lineage-biotin panel first)**				
CD71	BV510	113823	Biolegend	1:125
Ter119	PE/Cy7	116221	Biolegend	1:125
Gr1	APC	108411	Biolegend	1:125
CD11b	SparkUV387	101291	Biolegend	1:125
Biotin	Streptavidin-APC/Cy7	405208	Biolegend	1:125

Human HSPC panel				
**Target**	**Conjugate**	**Cat no.**	**Manufacturer**	**Dilution**
	shRNA-mCherry			
CD34	APC-Cy7	343513	Biolegend	1:125
CD45	PE	304007	Biolegend	1:125
CD43	PE-Cy7	343207	Biolegend	1:125
Dead	FVS450	562247	BD	1:250

Supplementary table S6

All antibodies, reagents and fluorochromes used in flow cytometry analysis of mouse transplantation assays and human HSPCs differentiated from H1 cells.

Antibody/Beads/Dyes	Cat no.	Manufacturer	Purpose
**Primary Ab**			
Rb anti-Ddx21	Nil (raised)	ABclonal	IF, IP, CUT&Tag, PLA
Rb anti-DDX21	10528-1-AP	Proteintech	IP, WB, ChIP
Ms anti-DDX21	sc376953	Santa Cruz	WB
Ms anti-Sin3a/SIN3A	5299	Santa Cruz	IF, IP, PLA, WB
Ms anti-Hdac1/HDAC1	sc81598	Santa Cruz	IF, IP, PLA, WB
Ms anti-Hdac2/HDAC2	5133	Cell Signaling Technologies	IF, IP, WB
Rb anti-Kdm5a/KDM5A	3876	Cell Signaling Technologies	CUT&Tag, IP, WB, ChIP
Ms anti-Kdm5a/KDM5A	GTX00688	Genetex	IF, IP, PLA, WB
Ms anti p53	CST2527	Cell Signaling Technologies	IF
Ms anti p-p53 (Ser15)	CST9284	Cell Signaling Technologies	IF
Rb anti-H3K27Ac	ab4729	Abcam	CUT&Tag
Rb anti-H3K27me3	9733	Cell Signaling Technologies	CUT&Tag
Rb anti-IgG	12-370	Milipore	CUT&Tag, IP, ChIP
Ms anti-IgG	sc2025	Santa Cruz	ChIP
Rb anti-H3K4me3	9751	Cell Signaling Technologies	CUT&Tag, WB, ChIP
Rb anti-H3K4me2	9725	Cell Signaling Technologies	WB
Rb anti-H3K4me1	ab8895	Abcam	CUT&Tag, WB
Rb anti-Total H3	A2348	ABclonal	WB
Rb anti-β-Actin/ACTIN	4970	Cell Signaling Technologies	WB
Ms anti-Flag	F1804	Sigma	WB, ChIP
Rb anti-Flag	PA1-984B	Life Technologies	WB
Rb anti-HA	71-5500	Life Technologies	IF, WB
Rb anti-V5	14440-1-AP	Proteintech	IP, WB
**Secondary Ab**			
Goat anti-mouse IgG AF488	A32723	Life Technologies	IF
Goat anti-rabbit IgG AF647	A32733	Life Technologies	IF
Goat anti-mouse IgG HRP	1706516	Bio-rad	WB
Goat anti-rabbit IgG HRP	1706515	Bio-rad	WB
**Beads**			
Anti-Flag magnetic beads	M8823	Sigma	IP
Anti-HA magnetic beads	88836	Life Technologies	IP
Anti-Twin Strep-tag Streptactin-XT magnetic beads	KTSM1350	AlpaLifeBio	IP
Anti-Ig Dynabeads Protein G	10004D	Life Technologies	IP
**Dyes**			
HRP dye for Twin Strep-tag detection	KTSM1550	AlpaLifeBio	WB
Streptavidin-HRP dye for biotin detection	S2438	Sigma	WB

Supplementary table S7

Antibodies, beads and dyes used in immunofluorescence (IF), immunoprecipitation (IP), CUT&Tag and western blot (WB), proximity ligation assay staining (PLA), and chromatin immunoprecipitation (ChIP).

Gene	Organism	Function	F	R
Pre-rRNA	Mouse	RT-qPCR	TTTTGGGGAGGTGGAGAGTC	AGAGAACTCCGGAGCACCAC
18S rRNA	Mouse	RT-qPCR	AGTCCCTGCCCTTTGTACACA	CGATCCGAGGGCCTCACTA
28S rRNA	Mouse	RT-qPCR	TGGGTTTTAAGCAGGAGGTG	GTGAATTCTGCTTCACAATG
Ddx21	Mouse	RT-qPCR	GACTTAATTGCCCAGGCACG	TGCAAGGACTAGCACCTGA
P21	Mouse	RT-qPCR	AACATCTCAGGGCCGAAAA	TGCGCTTGGAGTGATAGAAA
Mdm2	Mouse	RT-qPCR	GAAAAGCCTGAGGCTGGTAGAA	AACATAGGCAACCACCAGGAA
Bax	Mouse	RT-qPCR	GGAGCAGCTTGGGAGCG	AAAAGGCCCCTGTCTTCATGA
Gapdh	Mouse	RT-qPCR	TGGCCTTCCGTGTTCCTAC	GAGTTGCTGTTGAAGTCGCA
cKit	Mouse	RT-qPCR	TGGTCAAAGGAAATGCACGA	GCTCCCAGAGGAAAATCCCAT
Gata1	Mouse	RT-qPCR	TGGGGACCTCAGAACCCTTG	GGCTGCATTTGGGGAAGTG
Rps29	Mouse	RT-qPCR	GTCTGATCCGCAAATACGGG	AGCCTATGTCCTTCGCGTACT
Rps15	Mouse	RT-qPCR	GTCATTTACAGGATTCGTGTCCG	CAGGCTTGCCGTAAGTTGC
GATA1	Human	RT-qPCR	CTGTCCCCAATAGTGCTTATGG	GAATAGGCTGCTGAATTGAGGG
Pre-rRNA	Human	RT-qPCR	GGCGGTTTGAGTGAGACGAGA	ACGTGCGCTCACCGAGAGCAG
GATA1	Human	CHIP-qPCR	GGGTACAGCCACATCCCTTC	AAGCCCTTAATTTGTGCGGG
rDNA	Human	CHIP-qPCR	TTTGAGATCGTGCTCTCGGC	CTCTGCCTAGCTCACAGCG

Supplementary table S8

Primers used in RT-qPCR and CHIP-qPCR.
